# Green Oxidation of Aromatic Hydrazide Derivatives Using an Oxoammonium Salt

**DOI:** 10.1021/acs.joc.3c02752

**Published:** 2024-04-03

**Authors:** Nidheesh Phadnis, Jessica A. Molen, Shannon M. Stephens, Shayne M. Weierbach, Kyle M. Lambert, John A. Milligan

**Affiliations:** † Department of Biological and Chemical Sciences, College of Life Sciences, Thomas Jefferson University, 4201 Henry Ave, Philadelphia, Pennsylvania 19144, United States; ‡ Department of Chemistry and Biochemistry, Old Dominion University, 4501 Elkhorn Ave, Norfolk, Virginia 23529, United States

## Abstract

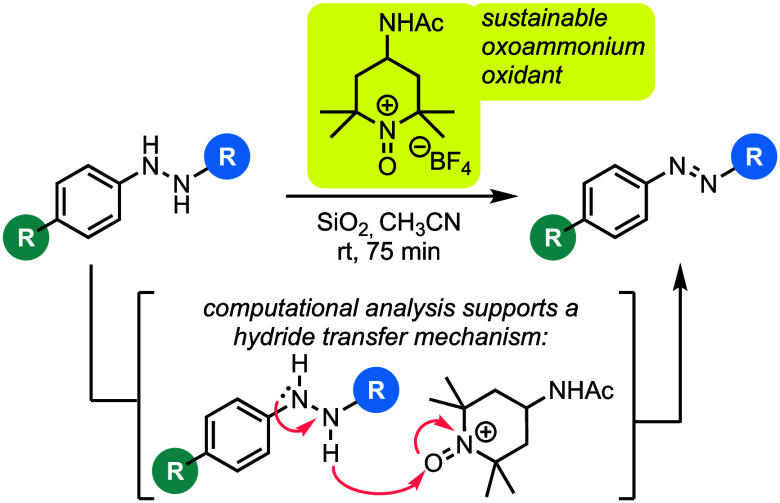

Aromatic diazenes are often prepared by oxidation of the corresponding hydrazides using stoichiometric quantities of nonrecyclable oxidants. We developed a convenient alternative protocol for the oxidation of aromatic hydrazides using Bobbitt’s salt (**1**), a metal-free, recyclable, and commercially available oxoammonium reagent. A variety of aryl hydrazides were oxidized within 75 min at room temperature using the developed protocol. Computational insight suggests that this oxidation occurs by a polar hydride transfer mechanism.

Wide-ranging efforts to advance the agenda of green chemistry continue to drive innovations in organic chemistry. Oxidation reactions present a particularly compelling opportunity for sustainable reaction development, as many of the most reliable and widely used oxidation protocols use stoichiometric, nonrecyclable reagents, including some that contain heavy metals.^1^ Oxoammonium salts and related nitroxides are among the most promising candidates to supplant classical oxidizing agents in organic synthesis.^2^

The bench-stable oxoammonium salt 4-acetamido-2,2,6,6-tetramethylpiperidine-1-oxoammonium tetrafluoroborate (**1**), also known as Bobbitt’s salt, is readily prepared on multimole scale in aqueous media and is isolated as a bright yellow solid.^3^ Reactions using **1** tend to be colorimetric in nature (the yellow color fades as the oxidation proceeds) and the resulting spent oxidant can be regenerated.^4^ Bobbitt’s salt is capable of a wide range of oxidations,^2,5^ including chemoselective transformations that require subtle discrimination of oxidizable functional groups.^6^ Protocols using **1** are operationally simple and robust, thereby allowing integration into the undergraduate laboratory curriculum.^7^

Our research group recently encountered the need to oxidatively prepare azocarboxylate derivatives as part of our efforts to develop strategies for heterocycle synthesis. Azocarboxylates, azobenzenes, and related diazenes have wide utility in a variety of applications, including use as photoswitches and dyes.^8^ They are also useful synthetic reagents in their own right: for example, azocarboxylates such as **3** have recently been shown to be effective aryl radical precursors,^9^ dienophiles/dipolarophiles,^10^ and Mitsunobu reagents.^11^ Consequently, various approaches to oxidatively synthesize diazenes from the corresponding hydrazides have been developed, the majority of which use stoichiometric oxidants such as Selectfluor, H_2_O_2_, K_3_Fe(CN)_6_, MnO_2_, or *N*-bromosuccinimide, among others.^12^ Several innovative catalytic approaches have recently been developed to improve the sustainability of these oxidations. For example, visible light-enabled^13^ as well as iron,^14^ copper^15^ and TEMPO^16^ -based catalyst systems can facilitate aerobic oxidations of hydrazides or diaryl hydrazines. However, the need for effective light or air penetration could potentially limit the scalability of these methods. Thus, we sought to apply Bobbitt’s salt (**1**) as a convenient, recyclable, and viable alternative to access a wide range of diazenes from aryl hydrazides and to study the underlying mechanism of this oxidation (Figure 1).

**Figure 1:**
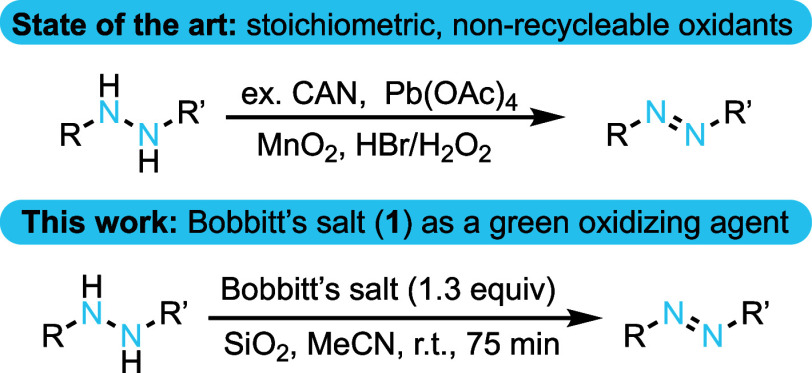
Known methods for oxidizing aromatic hydrazines and the context of this work.

Our starting point for reaction development was derived from our protocol for the oxidation of cinnamyl alcohol using **1**.^7^ Preliminary studies indicated that simply adding the oxoammonium salt to a solution of hydrazide at room temperature induced rapid oxidation of aryl hydrazides, as evidenced by a color change from yellow (due to the oxidant) to red (due to the highly colored diazene product). Silica gel was added as a solid support to accelerate the rate of oxidation (a phenomenon that was previously described by Bobbitt) and to simplify the workup procedure.^3b^ A control experiment without SiO_2_ revealed a modest decrease in yield if SiO_2_ gel is omitted (e.g., oxidation of **2** → **3**, 39% vs 52%, Table 1).

**Table 1:**
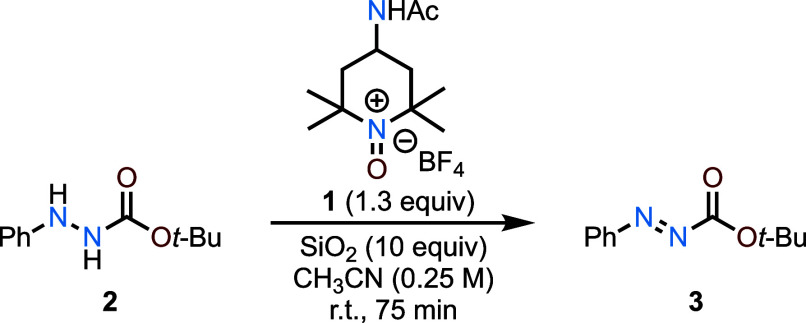
Optimization of the Reaction Parameters

entry	deviation from standard conditions	yield^a^ (%)
1	none	84
2	0.5 M concentration	80
3	0.1 M concentration	79
4	CH_2_Cl_2_ as solvent	52
5	CH_2_Cl_2_ as solvent; no SiO_2_; 60 min	39
6	CH_2_Cl_2_ as solvent; 1 equiv **1**; 60 min	31
7	CH_2_Cl_2_ as solvent; 2 equiv **1**; 60 min	27
8	toluene as solvent	29
9	DCE as solvent	52

a Yield determined by HPLC analysis with caffeine as an internal standard.

A screening of solvents revealed acetonitrile to be the optimal choice, although the reaction was moderately effective in halogenated solvents (Table 1). Hydrocarbon solvents such as toluene were less effective, as **1** is not readily soluble in these solvents. The nature of the oxidizing agent precluded the use of most protic or other oxidizable solvents for the reaction, apart from methanol which is not as readily oxidized. The reaction was rather insensitive to concentration and required at least 1 mol equiv of oxidant.

The oxidation of several aryl hydrazide derivatives was accomplished using the standardized conditions that employed 1.3 equiv of **1** in a SiO_2_/acetonitrile slurry at 23 °C for a period of 75 min (Scheme 1). A series of carbamate derivatives reacted smoothly (e.g., **3**–**5**). The reaction was easily scalable, as demonstrated by the 85% yield of **3** obtained when the reaction was conducted on a 1 g (4.7 mmol) scale. Both *N*-carbamoyl and *N*-tosyl substituted hydrazides are oxidized in comparably good yield (54–94%) under the reaction conditions, a feature that distinguishes this approach from related hydrazide oxidations that found substantially lower reactivity of tosyl hydrazides as compared to carbamate-substituted derivatives.^12,13^ Diaryl hydrazides could also be oxidized to the corresponding azobenzenes (**7** and **8**). Acyl derivatives were much less effective due to off-target oxidation reactivity in MeCN that resulted in an overall low mass recovery (e.g., **25**).^17^ However, phosphonyl-protected diazenes, which are strikingly under-represented in the literature, can be prepared (e.g., **9**).

**Scheme 1:**
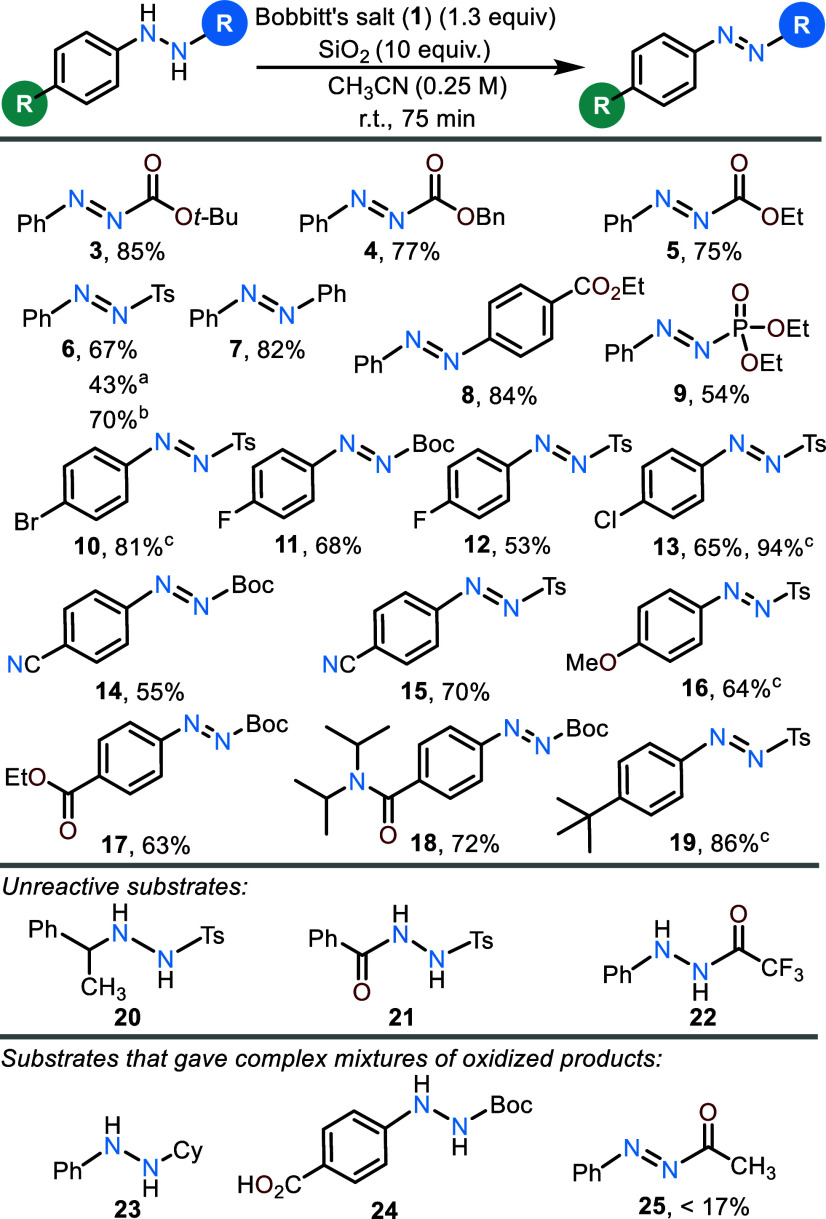
Scope of the Oxidation of Hydrazides and Hydrazines to Diazenes with **1** Isolated yield obtained from using recovered **1** on 1 g scale. Conducted in MeOH. Conducted without SiO_2,_ and the reaction time was 15 min.

Various aryl substituents (i.e., halogens, nitriles, nitro groups, esters, and amides; **10**–**19**) were tolerated in the reaction, while benzoyl hydrazide **21** was found to be nonreactive under our conditions. Hydrazides that do not benefit from conjugative stabilization by an aryl ring also fail to react, as demonstrated by **20**. The reaction does not appear to tolerate free carboxylic acids, as noted by the lack of successful oxidation of **24**. Electron-rich aryl hydrazide **16** gave a complex reaction profile under the standard conditions, suggesting undesired oxidative decomposition of the diazene product. Further investigation revealed that the oxidation of electron-rich systems was particularly facile and these systems did not require the use of SiO_2_. The resulting diazene products were found to be more susceptible to decomposition under extended reaction times. Thus, by removal of the silica gel and reducing the reaction time from 75 to 15 min, **16** was accessible in 64% isolated yield. This modification could also be used to access tosyl hydrazides **10**, **13**, and **19** in 81%, 94%, and 86% isolated yields, respectively.

Bobbitt’s salt (**1**) can accomplish chemoselective oxidations.^6^ We sought to explore whether a similar chemoselectivity could be observed in the oxidation of **26**, which contains both an aryl hydrazide and a primary alcohol. Exposure of **26** to one molar equivalent of **1** under the standard reaction conditions afforded only the alcohol-containing diazene **27** with no evidence of oxidation to the corresponding aldehyde (e.g., **27a**) (Scheme 2). This affirms that hydrazide oxidation with **1** is rather facile, allowing for oxidation in the presence of free aliphatic alcohols. The high degree of chemoselectivity of the reaction permits the use of methanol as a “greener” solvent without a decrease in isolated yields as demonstrated by its use to access **6** in 70% yield (Scheme 1). The spent oxidant can also be recovered and recycled for subsequent use in oxidations by using the protocol advanced by Bobbitt for recovery of the oxidant on multigram scale.^3b^ This protocol was demonstrated on a smaller 1 g scale by recovering the spent oxidant from an oxidation that gave **6** in a 67% yield. The regeneration of **1** for a second use afforded **6** in a slightly reduced yield of 43% (Scheme 1), thus the oxidant can be recovered to further enhance the sustainability of the process on larger scales.

**Scheme 2:**
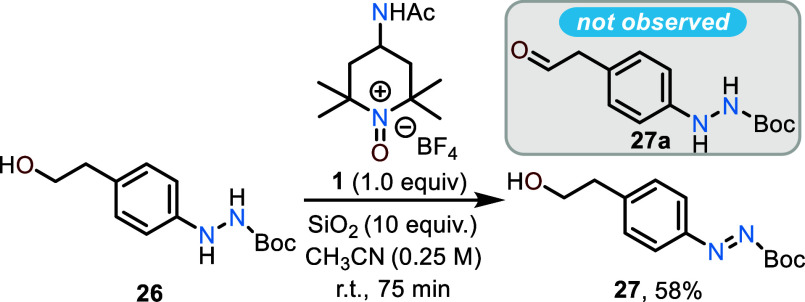
Chemoselective Oxidation of Hydrazide **26** to Diazene **27** Using **1**

Provided with the experimental observation that electron-rich aryl hydrazides generally oxidized faster than electron-deficient substrates, we surmised that the reaction may proceed via a hydride-removal mechanism.^7,18^ This mechanistic possibility was studied computationally at the B3LYP/6-311+G** level in the gas phase and at 298 K using 1,2-diphenylhydrazine **28** as a model substrate for oxidation by **1**. Our investigations reveal that the oxidation likely proceeds via a facile hydride transfer from the nitrogen of **28** to the oxygen of **1** with the formation of a N=N bond. The transition state for this process had one imaginary frequency (−1131 cm^−1^) and a small activation free energy of Δ*G*^‡^ = 4.7 kcal/mol relative to the reactants. The initial oxidation products (protonated azobenzene **29** and hydroxylamine **30**) were more stable than the reactants by 12.9 kcal/mol. Subsequently, **30** was found to abstract a proton from **29** in a deprotonation step that had one imaginary frequency (−618 cm^–1^) and a near barrierless activation free energy of Δ*G*^‡^ = 0.9 kcal/mol relative to the reactants, which aligns with the fact that no external base is necessary. This leads to the final oxidation product azobenzene (**7**) and the reduced form of **1** as protonated hydroxylamine **31**, which are both observed experimentally (Figure 2). Relevant Hirshfeld atomic charges for the starting materials and transition states were computed in the gas phase and show the transfer of charge, q (e)_,_ from N_a_ (−0.099 in **28** and −0.013 in TS1) to N_c_ (0.234 in **1** and 0.049 in TS1) through the loss of a hydride (Figure 2).

**Figure 2:**
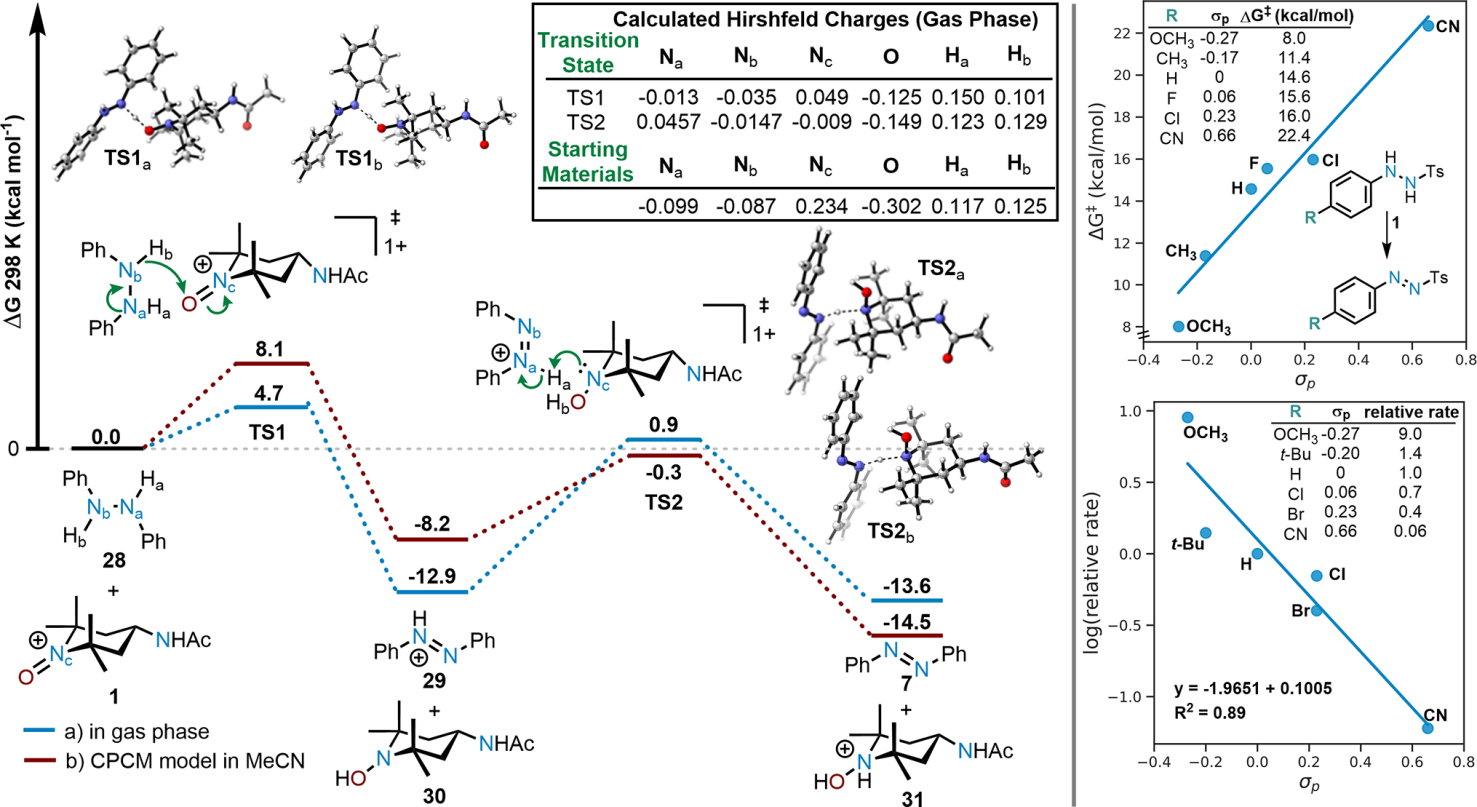
Left: Reaction coordinate diagram for the oxidation of diphenyl hydrazine (**28**) by (**1**) to azobenzene (**7**) calculated at 298 K in a) the gas phase at the B3LYP/6-311+G** level and b) using a CPCM solvation model for MeCN at the B3LYP-D3/6-311+G** level. Right: Plot of calculated activation free energies for the oxidation of *N*-aryl-*N*′-tosylhydrazines with **1** at the B3LYP/6-311+G** level in the gas phase versus σ_p_ values for corresponding aryl substituents (top), and Hammett plot of the relative rates of oxidation of *N*-aryl-*N*′-tosylhydrazines with **1** versus σ_p_ values for corresponding aryl substituents (bottom).

The influence of the acetonitrile solvent was studied computationally at the B3LYP-D3/6-311+G** level using the conductor-like polarizable continuum model (CPCM) at 298 K with **28** as a model substrate for oxidation by **1**. These results are shown in Figure 2 and demonstrate that, in comparison to the gas phase calculations, the inclusion of dispersion along with the solvent increase the energy of the first transition state (Δ*G*^‡^ = 8.1 kcal/mol) and slightly stabilize the second transition state (Δ*G*^‡^ = −0.3 kcal/mol). Both methods indicate a low activation barrier.

The effect of aryl substituents on the oxoammonium salt-mediated oxidation of several *N*-aryl-*N*′-tosylhydrazines containing electronically varied *para*-substituents (OCH_3_, CH_3_, H, Cl, F, and CN) was computationally studied (Figure 2, top right). Comparison of the computed activation free energies (Δ*G*^‡^) of these oxidations to the relevant σ-para values^19^ reveals a clear linear correlation (R^2^ = 0.95), as would be expected for a hydride abstraction mechanism. Overall, the computed activation free energies for the oxidation of *N*-aryl-*N*′-tosylhydrazines were higher than diarylhydrazines; Δ*G*^‡^ = 14.6 kcal/mol for *N*-phenyl-*N*′-tosylhydrazine versus Δ*G*^‡^ = 4.7 kcal/mol for **28**. This aligns with our experimental observation that azobenzenes **7** and **8** formed faster than the other diazenes that we prepared.

An experimental Hammett study was conducted using six different *N*-aryl-*N*′-tosylhydrazines containing electronically varied *para*-substituents (OCH_3_, *t*-Bu, H, Cl, Br, and CN). The relative rate of oxidation was measured in CD_3_CN by adding an equimolar amount of each *N*-aryl-*N*′-tosylhydrazine, *N*-phenyl-*N*′-tosylhydrazine and **1**_**,**_ then upon completion of the oxidation (10 min), the ^1^H NMR spectra were recorded. The relative rate of oxidation of each of the hydrazines (*k*) by **1** versus *N*-phenyl-*N*′-tosylhydrazine (*k*_0_) was determined using the molar ratios obtained by the ^1^H NMR integration of nonoverlapping signals of the two resulting diazene products. The logarithm of the experimentally determined relative rates (log k/*k*_0_) were then plotted versus the known σ_p_ values for the substituents (Figure 2, bottom right). The experimentally measured rho-value of −1.97 is indicative of positive charge buildup in the rate-limiting step as would be expected for a hydride transfer-type mechanism.

In summary, the oxidation of hydrazides and hydrazines using Bobbitt’s salt (**1**) provides an experimentally straightforward and cost-effective method for the synthesis of aryl diazenes. Computational analysis of the oxidation suggests that the rate limiting step is hydride transfer from the nitrogen of the hydrazide derivative to the oxygen of the oxoammonium salt. Advancements in the understanding of the scope, limitations, and mechanistic underpinnings of the reaction between oxoammonium salts and hydrazines, as well as hydrazides described in this work, will allow further development of green oxidation strategies for other nitrogen-containing functional groups in the future.

## Data Availability

The data underlying this study are available in the published article and its Supporting Information

## Supporting Information Available

Detailed experimental procedures, computational methods, atomic coordinates, and NMR spectra of synthesized products (PDF)
