# Supplementary material for: Green Oxidation of Aromatic Hydrazide Derivatives Using an Oxoammonium Salt
Source: J Org Chem. 2024 Apr 3;89(8):5841–5. doi: 10.1021/acs.joc.3c02752 (PMC11040711; doi:10.1021/acs.joc.3c02752)

Supporting Information for

"Green Oxidation of Aromatic Hydrazide Derivatives Using an Oxoammonium Salt"

Nidheesh Phadnis[1], Jessica A. Molen[1], Shannon M. Stephens[2], Shayne M. Weierbach[2], Kyle M. Lambert[2]* and John A. Milligan[1]*

[1]Department of Biological and Chemical Sciences, College of Life Sciences, Thomas Jefferson University, 4201 Henry Ave, Philadelphia, Pennsylvania 19144, United States

[2] Department of Chemistry and Biochemistry, Old Dominion University, 4501 Elkhorn Ave, Norfolk, Virginia 23529, United States

<u>Table of contents</u>

General chemistry methods S2
Synthesis hydrazide precursors S3
Oxidation of hydrazides........ S6
Experimental details for Hammett study........ S13
Computational details S13
References........ S65
NMR spectra of new compounds S66

General chemistry methods

NMR spectra (^{1}H, ^{13}C, and ^{31}P) were obtained at 298 K. ^{1}H NMR spectra were referenced to residual, non-deuterated chloroform (δ 7.26) in $CDCl_3$. ^{13}C NMR spectra were referenced to $CDCl_3$ (δ 77.3). Reactions were monitored by ^{1}H NMR and/or TLC on silica gel plates (60 Å porosity, 250 µm thickness). TLC analysis was performed using hexanes/EtOAc as the eluant and visualized using permanganate stain, *p*-anisaldehyde stain, and/or UV light. Flash chromatography was carried out using standard column chromatography on silica gel (60 Å porosity, 32-63 µm). High Resolution mass spectra (HRMS) were obtained at Old Dominion University on a Thermo Scientific LTQ Orbitrap XL Hybrid Ion Trap spectrometer using electrospray ionization operating the positive mode ($^{+}$ESI), collected using the Orbitrap mass analyzer, and are reported for the molecular ion ($[M+H]^{+}$ or $[M+Na]^{+}$ respectively).

Synthesis of hydrazide precursors

The synthesis of the following substituted hydrazides has previously been described in the corresponding references:

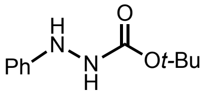

2[1]

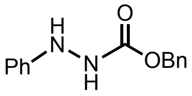

S4[2]

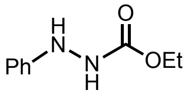

S5[2]

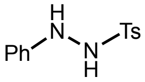

S6[1]

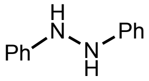

S7[3]

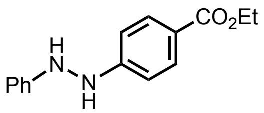

S8[3]

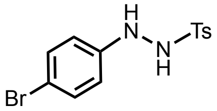

S10[5]

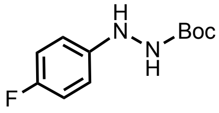

S11[4]

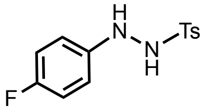

S12[5]

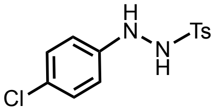

S13[5]

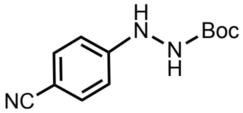

S14[6]

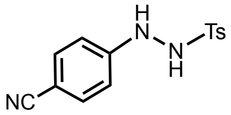

S15[5]

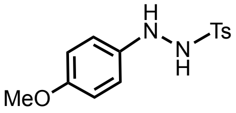

S16[5]

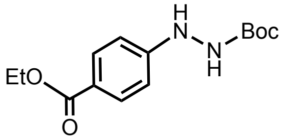

S17[7]

Synthesis of new hydrazide precursors

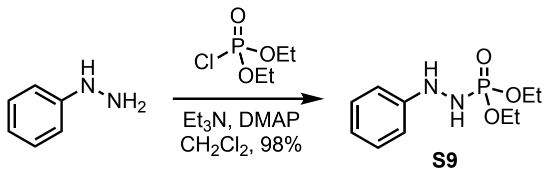

S9

Phenylhydrazine (0.49 mL, 5.0 mmol, 1.0 equiv), 4-dimethylaminopyridine (61 mg, 0.50 mmol, 0.10 equiv) and Et_3N (0.70 mL, 5.0 mmol, 1.0 equiv) were dissolved in CH_2Cl_2 (20 mL) at 0 °C. After the reaction mixture was stirred for 10 min at 0 °C, diethylphosphinyl chloride (0.72 mL, 5.0 mmol, 1.0 equiv) was added. The reaction mixture was stirred for 4 h before being quenched with saturated aqueous sodium chloride. The mixture was diluted with ethyl acetate and washed with saturated aq $NaHCO_3$. The organic layer was dried over anhydrous Na_2SO_4, filtered, and concentrated under reduced pressure to give **S9** (1.29 g, 98%) as a colorless solid: Melting point: 73–76 °C; ^{1}H NMR (400 MHz, $CDCl_3$) δ 7.24 (t, 2 H, J = 8.0 Hz), 6.94 (d, 2 H, J = 7.6 Hz), 6.89 (t, 1 H, J = 7.2 Hz), 5.21-5.02 (br, 2 H), 4.23-4.03 (m, 4 H), 1.24 (t, 6 H, J = 7.2 Hz); ^{31}P{^{1}H} NMR (161 MHz, $CDCl_3$) δ 5.9 (s, 1 P); ^{13}C{^{1}H} NMR (100 MHz, $CDCl_3$) δ 148.8, 128.9, 120.2, 113.2, 106.5, 63.4 (d, $^3J_{CP}$ = 6 Hz), 16.1 (d, $^4J_{CP}$ = 6 Hz); IR (ATR) 3297 (w), 2980 (w), 1648 (m), 1603 (w), 1497 (w), 1214 (m), 1026 (s), 970 (m), 748 (m); HRMS (ESI$^+$-Orbitrap) m/z calc'd for $C_{10}H_{17}N_2O_3PNa$ [M+Na$^+$]: 267.0874, found 267.0872.

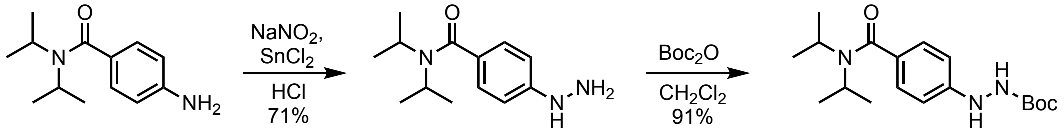

S18

A solution of amide[8] (0.403 g, 1.83 mmol) was dissolved in concentrated HCl (2 mL) and cooled to 0 °C. A solution of $NaNO_2$ (0.1 g, 1.1 eq.) in 2 mL of deionized water was added over 2 minutes. The solution was stirred for further 2 minutes at 0 °C, after which a solution of $SnCl_2$ (0.761 g, 4.03 mmol, 2.20 eq.) in concentrated HCl (2 mL) was added dropwise, which caused formation of a precipitate. The reaction was stirred for 3 hours at 0 °C. The reaction was basified with 8 M NaOH and extracted with CH_2Cl_2 (3 x 30 mL). The organic layer was dried with brine (30 mL), dried (Na_2SO_4), and concentrated to afford the desired hydrazine (0.306 g, 71%) as a red/brown solid.

A solution of the hydrazine (0.195 g, 0.83 mmol) in CH_2Cl_2 (3 mL) was treated with Boc_2O (0.181 g, 0.83 mmol, 1.00 equiv). The reaction was heated to reflux (oil bath) for 3 h, then was cooled to rt and partitioned between water and CH_2Cl_2 (30 mL each). The organic layer was dried and concentrated. Purification by chromatography on SiO_2 afforded **S18** (0.252 g, 91%) as a buff solid: Melting point: 179-181 °C ; ^{1}H NMR (400 MHz, $CDCl_3$) δ 7.16 (d, 2 H, J = 8.4 Hz), 6.72 (d, 2 H, J = 8.4 Hz), 4.03-3.44 (br, 2 H), 1.51 (s, 9 H), 1.44-1.08 (br, 12 H); ^{13}C {^{1}H} NMR (100 MHz, $CDCl_3$) δ 171.5, 156.4, 149.0, 130.4, 127.1, 125.8, 112.2, 81.0, 28.3, 20.9; IR (ATR) 3326 (w), 3192 (w), 2970 (w), 2932 (w), 1701 (m), 1609 (s), 1442 (m), 1345 (m), 1249 (m), 1161 (s), 1036 (w), 825 (m) 701 (w); HRMS (ESI$^+$-Orbitrap) m/z calc'd for $C_{18}H_{30}N_3O_3$ [M+H]: 336.2287, found 336.2285.

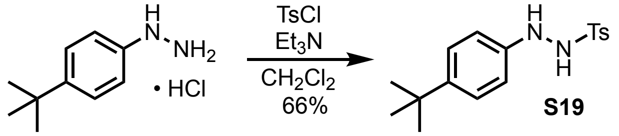

A slurry of 4-*tert*-butylphenylhydrazine hydrochloride (0.200 g, 1.00 mmol) in CH_2Cl_2 (5 mL) was cooled to 0 °C in an ice/water bath and treated with triethylamine (0.278 mL, 0.202 g, 2.00 mmol) followed by tosyl chloride (0.096 g, 0.5 mmol). The reaction mixture was stirred overnight for 12 h, at which point the stir bar was removed and 0.500 g of silica gel was added. The mixture was then concentrated, and the resulting silica gel mixture was dry-loaded onto 12 g of SiO_2 and the crude material was purified by column chromatography (0 to 30% EtOAC/hexanes) to afford **S19** (0.105 g, 66%) as a colorless crystalline solid: ^{1}H NMR (400 MHz, $CDCl_3$) δ 7.74 (d, 2 H, *J* = 8.4 Hz), 7.23 (d, 2 H, *J* = 8.3 Hz), 7.12 (d, 2 H, *J* = 8.8 Hz), 6.67 (d, 2 H, *J* = 8.8 Hz), 6.31 (br s, 2H, exchanges), 2.39 (s, 3 H), 1.23 (s, 9 H); ^{13}C {^{1}H} NMR (100 MHz, $CDCl_3$) δ 144.21, 144.19, 143.74, 135.0, 129.6, 128.3, 125.8, 113.5, 34.0, 31.4, 21.6; HRMS (ESI$^+$-Orbitrap) calc'd for $C_{17}H_{23}N_2O_2S$ [M+H$^+$]: 319.1475, found 319.1491, and calc'd for $C_{17}H_{22}N_2O_2SNa$ [M+Na$^+$]: 341.1294, found 341.1310.

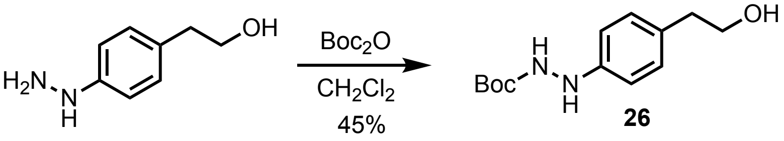

A solution of hydrazine[8] (0.486 g, 3.20 mmol) in CH_2Cl_2 (8 mL) was treated with Boc_2O (0.698 g, 3.20 mmol, 1.00 equiv). The reaction was heated (oil bath) to reflux for 2 h, then was stirred at rt for 16 h more. The material was concentrated. Purification by chromatography on SiO_2 afforded **26** (0.482 g, 45%) as a brown-tinted oil: ^{1}H NMR (400 MHz, $CDCl_3$) δ 7.08 (d, 2 H, *J* = 8.4 Hz), 6.78 (d, 2 H, *J* = 8.4 Hz), 3.79 (t, 2 H, *J* = 6.4 Hz), 2.78 (t, 2 H, *J* = 6.4 Hz), 1.47 (s, 9 H); ^{13}C {^{1}H} NMR (100 MHz, $CDCl_3$) δ 156.5, 146.9, 130.6, 129.9, 129.7, 128.9, 123.6, 113.2, 81.2, 63.7, 38.3, 28.4; IR (ATR) 3364 (w), 2932 (s), 1748 (m), 1508 (w), 1395 (w), 1254 (s), 1138 (s), 1042 (m), 837 (w); HRMS (ESI$^+$-Orbitrap) calc'd for $C_{13}H_{20}N_2O_3Na$ [M+Na$^+$]: 275.1372, found 275.1370.

Oxidation of hydrazides

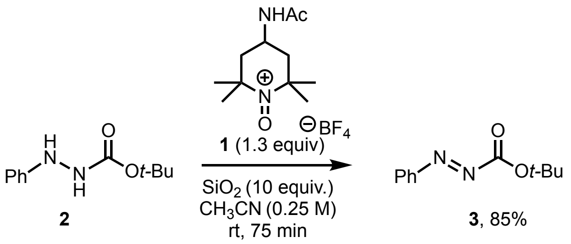

General Procedure for hydrazide oxidation: A 100 mL round bottom flask as charged with hydrazide **2** (0.980 g, 4.70 mmol, 1.00 equiv) and MeCN (20 mL, 0.25 M). Silica gel (2.82 g, 47.0 mmol, 10.0 equiv.) and Bobbitt's Salt (**1**) (1.84 g, 6.11 mmol, 1.30 equiv.) were added to the flask. The reaction was stirred for 75 min open to the air at room temperature. The mixture was filtered through a frit containing a bed of silica gel and rinsed forward with CH_2Cl_2. The filtrate was concentrated *in vacuo*. Purification by chromatography on SiO_2 (10-25% EtOAc/hexanes) afforded the product **3** (0.822 g, 85%) as a red-tinted oil: ^{1}H NMR (400 MHz, $CDCl_3$) δ 7.90 (d, 2 H, *J* = 7.6 Hz), 7.58-7.42 (m, 3 H), 1.66 (s, 9 H). The spectroscopic data of **3** were in accord with previous reports.[1]

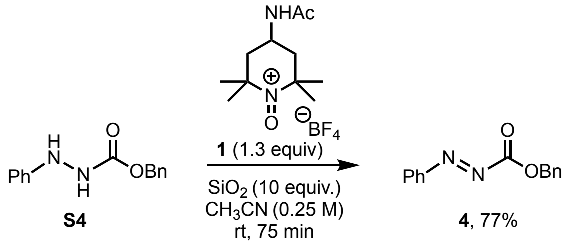

Prepared according to the general procedure from hydrazide **S4** (0.242 g, 1.00 mmol, 1.00 equiv) and MeCN (4 mL), silica gel (0.601 g) and Bobbitt's Salt (**1**) (0.390 g, 1.30 mmol, 1.30 equiv.). Purification by chromatography on SiO_2 (10-25% EtOAc/hexanes) afforded the product **4** (0.185 g, 77%) as a red-tinted oil: ^{1}H NMR (400 MHz, $CDCl_3$) δ 7.94 (d, 2 H, *J* = 8.0 Hz), 7.57-7.42 (m, 5 H), 7.41-7.35 (m, 3 H), 5.49 (s, 3 H). The spectroscopic data of **4** were in accord with previous reports.[2]

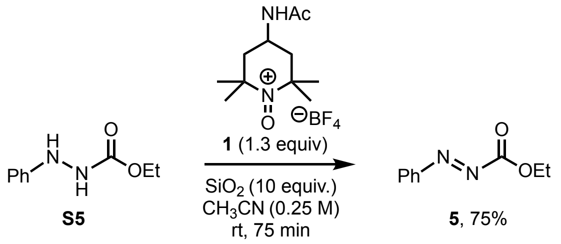

Prepared according to the general procedure from hydrazide **S5** (0.180 g, 1.00 mmol, 1.00 equiv) and MeCN (4 mL), silica gel (0.601 g) and Bobbitt's Salt (**1**) (0.390 g, 1.30 mmol, 1.30 equiv.). Purification by chromatography on SiO_2 (10-25% EtOAc/hexanes) afforded the product **5** (0.134 g, 75%) as a red-tinted oil: ^{1}H NMR (400 MHz, $CDCl_3$) δ 7.89 (d, 2 H, *J* = 8.0 Hz), 7.58-7.40 (m, 3 H), 4.47 (q, 2 H, *J* = 6.8 Hz), 1.41 (t, 3 H, *J* = 7.2 Hz). The spectroscopic data of **5** were in accord with previous reports.[2]

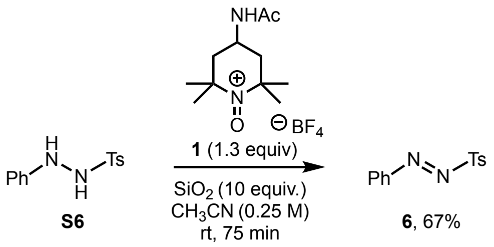

Prepared according to the general procedure from hydrazide **S6** (0.171 g, 0.65 mmol, 1.00 equiv) and MeCN (2.6 mL), silica gel (0.391 g) and Bobbitt's Salt (**1**) (0.254 g, 0.85 mmol, 1.30 equiv.). Purification by chromatography on SiO_2 (10-25% EtOAc/hexanes) afforded the product **6** (0.113 g, 67%) as a red-tinted oil: ^1H NMR (400 MHz, $CDCl_3$) δ 7.86 (d, 2 H, *J* = 6.8 Hz), 7.81-7.77 (m, 1 H), 7.74-7.72 (m, 1 H), 7.60-7.54 (m, 1 H), 7.51-7.44 (m, 2 H), 7.41-7.36 (m, 2 H), 2.46 (s, 3 H). The spectroscopic data of **6** were in accord with previous reports.[1]

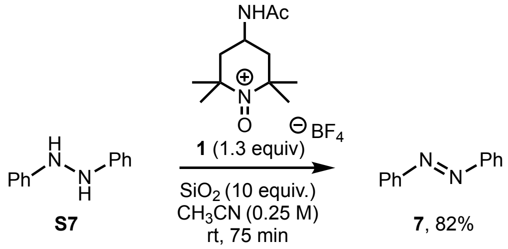

Prepared according to the general procedure from hydrazine **S7** (0.248 g, 1.35 mmol, 1.00 equiv) and MeCN (5.4 mL), silica gel (0.811 g) and Bobbitt's Salt (**1**) (0.527 g, 1.76 mmol, 1.30 equiv.), with the following modification: the reaction was complete after 35 minutes. Purification by chromatography on SiO_2 (10-25% EtOAc/hexanes) afforded azobenzene (**7**) (0.201 g, 82%) as a red/orange solid: ^1H NMR (400 MHz, $CDCl_3$) δ 7.95-7.90 (m, 4 H), 7.55-7.45 (m, 6 H). The spectroscopic data of **7** were in accord with commercial material.

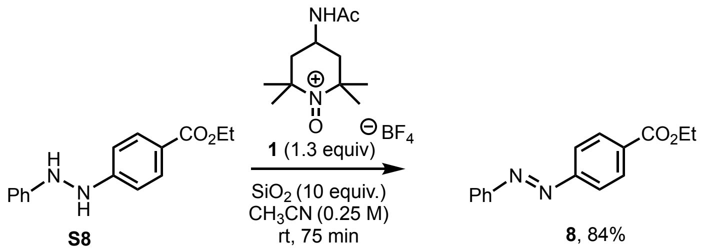

Prepared according to the general procedure from hydrazine **S8** (0.036 g, 0.14 mmol, 1.00 equiv) and MeCN (1.0 mL), silica gel (0.084 g) and Bobbitt's Salt (**1**) (0.055 g, 0.18 mmol, 1.30 equiv.), with the following modification: the reaction was complete after 45 minutes. Purification by chromatography on SiO_2 (10-25% EtOAc/hexanes) afforded **8** (0.031 g, 84%) as a red/orange solid: ^1H NMR (400 MHz, $CDCl_3$) δ 8.10 (d, 2 H, *J* = 8.8 Hz), 7.97-7.93 (m, 4 H), 7.56-7.48 (m, 3 H), 4.42 (q, 2 H, *J* = 7.2 Hz), 1.43 (t, 3 H, *J* = 7.2 Hz). The spectroscopic data of **8** were in accord with previous reports.[3]

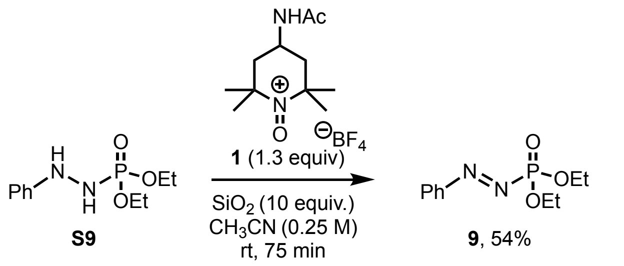

Prepared according to the general procedure from hydrazide **S9** (0.887 g, 3.63 mmol, 1.00 equiv) and MeCN (15 mL), silica gel (2.18 g) and Bobbitt's Salt (**1**) (1.42 g, 4.72 mmol, 1.30 equiv.) Purification by chromatography on SiO_2 (10-25% EtOAc/hexanes) afforded **9** (0.477 g, 54%) as a dark red oil: ^{1}H NMR (400 MHz, $CDCl_3$) δ 7.98-7.91 (m, 2 H), 7.61-7.56 (m, 1 H), 7.56-7.50 (m, 2 H), 4.44-4.30 (m, 4 H), 1.42 (td, 6 H, *J* = 6.8 Hz, 0.8 Hz); $^{13}C\{^1H\}$ NMR (100 MHz, $CDCl_3$) δ 154.2 ($^3J_{CP}$ = 53 Hz), 134.0, 129.2, 123.1 (d, $^4J_{CP}$ = 2 Hz), 64.8 (d, $^3J_{CP}$ = 7 Hz), 16.3 (d, $^4J_{CP}$ = 7 Hz); $^{31}P\{^1H\}$ NMR (162 MHz, $CDCl_3$) δ –0.60 (s, 1 P); IR (ATR) 2984 (w), 1493 (m), 1450 (w), 1265 (m), 1144 (w), 1014 (s), 980 (m), 880 (m), 806 (m), 766 (m), 685 (m) HRMS (ESI) *m/z* calc'd for $C_{10}H_{16}N_2O_3P$ [M+H]: 243.0899, found 243.0897.

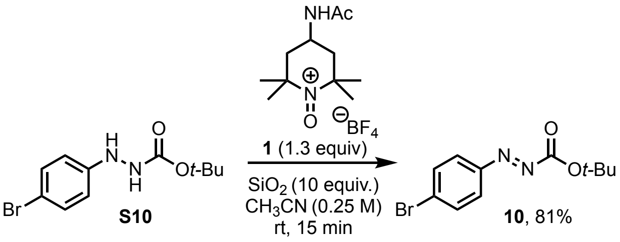

Prepared according to the general procedure with some modifications (no SiO_2 was used, and the reaction was run for 15 minutes) from hydrazide **S10** (0.070 g, 0.21 mmol, 1.00 equiv) using MeCN (2 mL), and Bobbitt's Salt (**1**) (0.080 g, 0.27 mmol, 1.3 equiv.). Purification by chromatography on SiO_2 (0-15% EtOAc/hexanes) afforded the product **10** (0.055 g, 81%) as a yellow crystalline solid: ^{1}H NMR (400 MHz, $CDCl_3$) δ 7.85 (d, 2 H, *J* = 8.3 Hz), 7.61-7.69 (m, 4 H), 7.39 (d, 2 H, *J* = 8.3 Hz), 2.47 (s, 3 H); $^{13}C\{^1H\}$ NMR (100 MHz, $CDCl_3$) δ 148.1, 146.4, 133.1, 130.6, 130.18, 130.15, 130.0, 126.0, 22.0. The spectroscopic data of **10** were in accord with previous reports.[5]

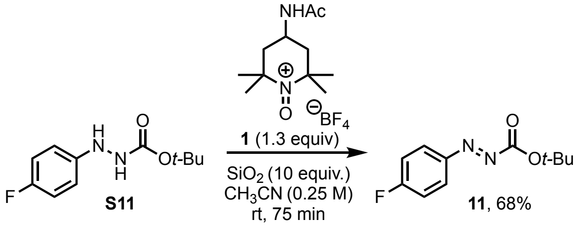

Prepared according to the general procedure from hydrazide **S11** (0.226 g, 1.00 mmol, 1.00 equiv) and MeCN (4 mL), silica gel (0.601 g) and Bobbitt's Salt (**1**) (0.390 g, 1.30 mmol, 1.30 equiv.). Purification by chromatography on SiO_2 (10-25% EtOAc/hexanes) afforded the product **11** (0.152 g, 68%) as a red-tinted oil: ^{1}H NMR (400 MHz, $CDCl_3$) δ 7.96-7.90 (m, 2 H), 7.22-7.15 (m, 2 H), 1.66 (s, 9 H); $^{19}F\{^1H\}$ NMR (376 MHz, $CDCl_3$) δ –105.2. The spectroscopic data of **11** were in accord with previous reports.[4]

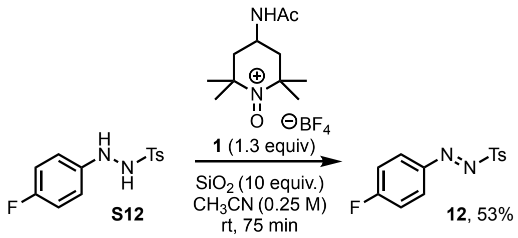

Prepared according to the general procedure from hydrazide **S12** (0.280 g, 1.00 mmol, 1.00 equiv) and MeCN (4 mL), silica gel (0.601 g) and Bobbitt's Salt (**1**) (0.390 g, 1.30 mmol, 1.30 equiv.). Purification by chromatography on SiO_2 (10-25% EtOAc/hexanes) afforded the product **12** (0.146 g, 53%) as a yellow solid: ^{1}H NMR (400 MHz, $CDCl_3$) δ 7.89-7.83 (m, 4 H), 7.41-7.37 (m, 2 H), 7.20-7.14 (m, 2 H), 2.48 (s, 3 H); ^{19}F NMR (376 MHz, $CDCl_3$) δ –102.0.The spectroscopic data of **12** were in accord with previous reports.[9]

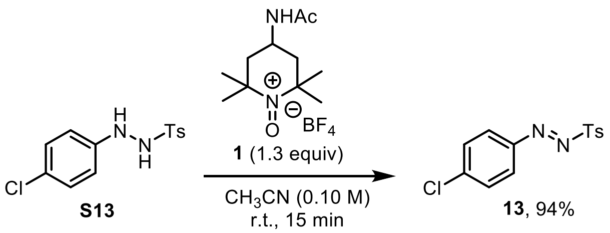

Prepared according to the general procedure with some modifications (no SiO_2 was used, and the reaction was run for 15 minutes) from hydrazide **S13** (0.050 g, 0.17 mmol, 1.00 equiv) using MeCN (2 mL), and Bobbitt's Salt (**1**) (0.066 g, 0.22 mmol, 1.3 equiv.). Purification by chromatography on SiO_2 (0-15% EtOAc/hexanes) afforded the product **13** (0.045 g, 94%) as a yellow crystalline solid: ^{1}H NMR (400 MHz, $CDCl_3$) δ 7.85 (d, 2 H, *J* = 8.3 Hz), 7.76 (apparent d, 2 H, *J* = 8.8 Hz), 7.46 (apparent d, 2 H, *J* = 8.8 Hz), 7.39 (d, 2 H, *J* = 8.3 Hz), 2.46 (s, 3 H). The spectroscopic data of **13** were in accord with previous reports.[9]

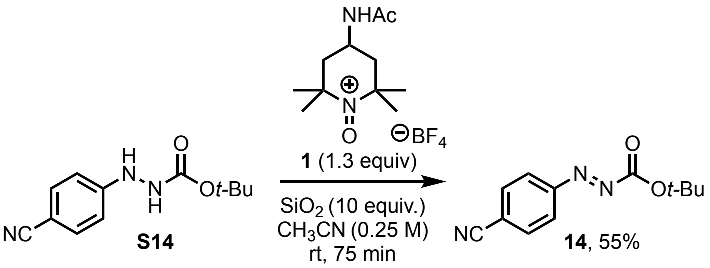

Prepared according to the general procedure from hydrazide **S14** (0.364 g, 1.56 mmol, 1.00 equiv) and MeCN (6.2 mL), silica gel (0.937 g) and Bobbitt's Salt (**1**) (0.608 g, 2.03 mmol, 1.30 equiv.). Purification by chromatography on SiO_2 (10-25% EtOAc/hexanes) afforded the product **14** (0.200 g, 55%) as a yellow solid: ^{1}H NMR (400 MHz, $CDCl_3$) δ 7.99 (d, 2 H, *J* = 8.8 Hz), 7.84 (d, 2 H, *J* = 8.8 Hz), 1.69 (s, 9 H). The spectroscopic data of **14** were in accord with previous reports.[4]

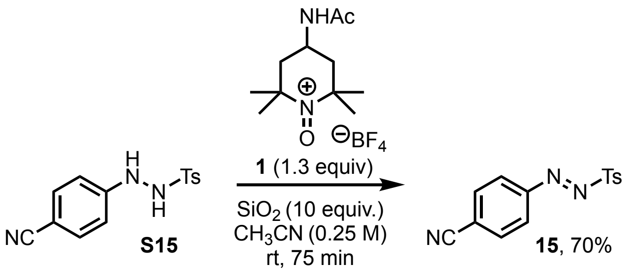

Prepared according to the general procedure from hydrazide **S15** (0.172 g, 0.60 mmol, 1.00 equiv) and MeCN (2.4 mL), silica gel (0.360 g) and Bobbitt's Salt (**1**) (0.234 g, 0.78 mmol, 1.30 equiv.). Purification by chromatography on SiO_2 (10-25% EtOAc/hexanes) afforded the product **15** (0.119 g, 70%) as a yellow/orange solid: ^1H NMR (400 MHz, $CDCl_3$) δ 7.91-7.90 (m, 1 H), 7.89-7.88 (m, 1 H), 7.88-7.86 (m, 1 H), 7.86-7.84 (m, 1 H), 7.82-7.80 (m, 1 H), 7.80-7.78 (m, 1 H), 7.43-7.39 (m, 2 H), 2.49 (s, 3 H). The spectroscopic data of **15** were in accord with previous reports.[4]

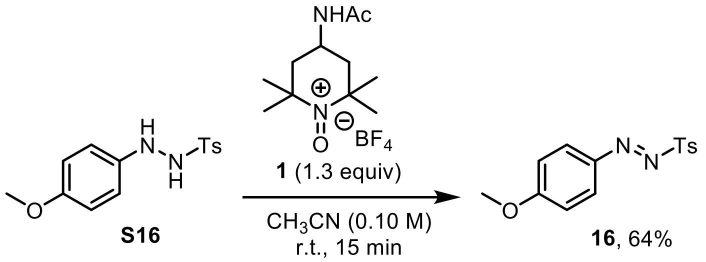

Prepared according to the general procedure with some modifications (no SiO_2 was used, and the reaction was run for 15 minutes) from hydrazide **S16** (0.088 g, 0.30 mmol, 1.00 equiv) using MeCN (3 mL), and Bobbitt's Salt (**1**) (0.120 g, 0.39 mmol, 1.3 equiv.). Purification by chromatography on SiO_2 (0-20% EtOAc/hexanes) afforded the product **16** (0.055 g, 64%) as a yellow crystalline solid: ^1H NMR (400 MHz, $CDCl_3$) δ 7.86 (d, 2 H, J = 8.3 Hz), 7.83 (apparent d, 2 H, J = 9.1 Hz), 7.37 (d, 2 H, J = 8.2 Hz), 6.96 (apparent d, 2 H, J = 9.1 Hz), 3.89 (s, 3 H), 2.46 (s, 3 H).The spectroscopic data of **16** were in accord with previous reports.[9]

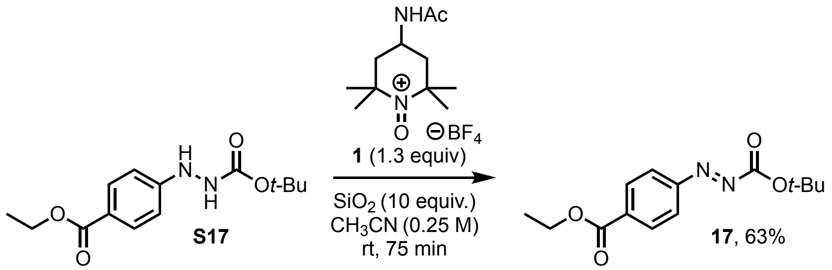

Prepared according to the general procedure from hydrazide **S17** (0.167 g, 0.500 mmol, 1.00 equiv) and MeCN (2 mL), silica gel (0.300 g) and Bobbitt's Salt (**1**) (0.195 g, 0.650 mmol, 1.30 equiv.). Purification by chromatography on SiO_2 (10-25% EtOAc/hexanes) afforded the product **17** (0.104 g, 63%) as a red oil: ^1H NMR (400 MHz, $CDCl_3$) δ 8.19 (d, 2 H, J = 8.0 Hz), 7.93 (d, 2 H, J = 8.4 Hz), 4.42 (q, 2 H, J = 7.6 Hz), 1.67 (s, 9 H), 1.42 (t, 3 H, J = 7.2 Hz); $^{13}C\{^1H\}$ NMR (100 MHz, $CDCl_3$) δ 165.5, 160.9, 153.8, 134.3, 130.6, 123.2, 85.5, 61.5, 27.9, 14.3; IR (ATR) 2981 (w), 1753 (m), 1717 (m), 1513 (w), 1370 (w), 1252 (s), 1141 (s), 1104

(s), 837 (w), 777 (w); HRMS (ESI$^+$-Orbitrap) *m/z* calc'd for $C_{14}H_{18}N_2O_4Na$ [M+Na$^+$]: 301.1164, found 301.1149.

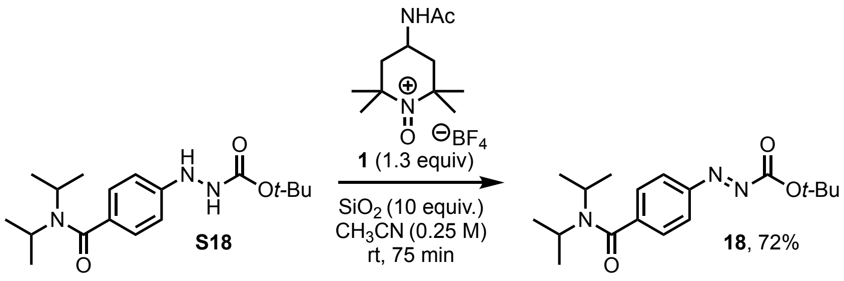

Prepared according to the general procedure from hydrazide **S18** (0.188 g, 0.560 mmol, 1.00 equiv) and MeCN (2.2 mL), silica gel (0.336 g) and Bobbitt's Salt (**1**) (0.218 g, 0.728 mmol, 1.30 equiv.). Purification by chromatography on SiO_2 (10-25% EtOAc/hexanes) afforded the product **18** (0.135 g, 72%) as a red oil: ^{1}H NMR (400 MHz, $CDCl_3$) δ 7.97-7.89 (m, 2 H), 7.50-7.42 (m, 2 H), 4.00-3.39 (br, 2 H), 1.65 (s, 9 H), 1.63-1.04 (br, 12 H); ^{13}C{^{1}H} NMR (100 MHz, $CDCl_3$) δ 169.6, 161.0, 151.2, 143.4, 126.5, 123.8, 85.1, (51.0, 46.0 (broad, methine rotomers)), 27.8, 20.6; IR (ATR): 2973 (w), 1751 (m), 1626 (m), 1441 (w), 1340 (m), 1254 (s), 1139 (s), 1035 (w), 835 (w), 729 (m); HRMS (ESI$^+$-Orbitrap) *m/z* calc'd for $C_{18}H_{28}N_3O_3$ [M+H] 334.2131, found 334.2148.

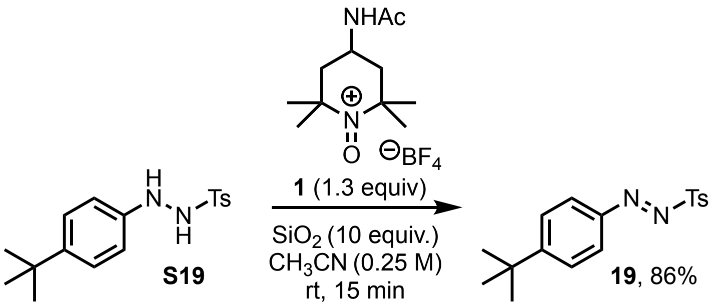

Prepared according to the general procedure with some modifications (no SiO_2 was used, and the reaction was run for 15 minutes) from hydrazide **S19** (0.100 g, 0.31 mmol, 1.00 equiv) using MeCN (3 mL), and Bobbitt's Salt (**1**) (0.121 g, 0.4 mmol, 1.3 equiv.). Purification by chromatography on SiO_2 (0-15% EtOAc/hexanes) afforded the product **19** (0.084 g, 86%) as a light yellow crystalline solid: ^{1}H NMR (400 MHz, $CDCl_3$) δ 7.85 (d, 2 H, *J* = 8.3 Hz), 7.75 (apparent d, 2 H, *J* = 8.8 Hz), 7.49 (apparent d, 2 H, *J* = 8.8 Hz), 7.38 (d, 2 H, *J* = 8.3 Hz), 2.46 (s, 3 H), 1.33 (s, 9 H). The spectroscopic data of **19** were in accord with previous reports.[10]

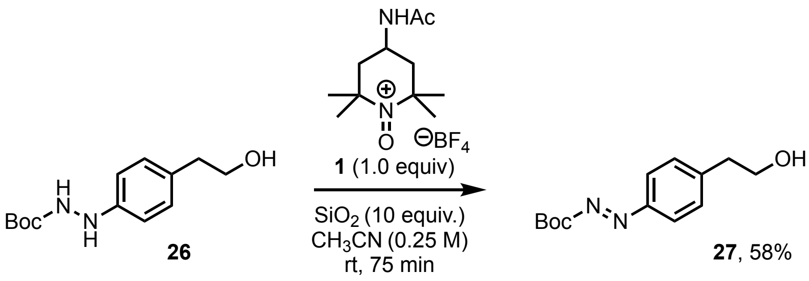

Prepared according to the general procedure from hydrazide **26** (0.076 g, 0.30 mmol, 1.00 equiv) and MeCN (1.2 mL), silica gel (0.180 g) and Bobbitt's Salt (**1**) (0.117 g, 0.390 mmol, 1.30 equiv.). Purification by chromatography on SiO_2 (10-25% EtOAc/hexanes) afforded the product **27** (0.035 g, 58%) as a red oil: ^{1}H NMR (400 MHz, $CDCl_3$) δ 7.86 (d, 2 H, J = 8.8 Hz), 7.37 (d, 2 H, J = 8.4 Hz), 3.92 (t, 2 H, J = 6.4 Hz), 2.95 (t, 2 H, J = 6.4 Hz), 1.66 (s, 9 H); ^{13}C{^{1}H} NMR (100 MHz, $CDCl_3$) δ 161.2, 150.3, 145.1, 129.9, 123.9, 85.0, 63.2, 39.1, 27.9; IR (ATR) 3364 (w), 2933 (w), 1748 (m), 1507 (w), 1395 (m), 1254 (m), 1138 (s), 1042 (w), 837 (w); HRMS (ESI$^+$-Orbitrap) m/z calc'd for $C_{13}H_{18}N_2O_3Na$ [M+Na$^+$]: 273.1215, found 273.1213.

Details of Experimental Hammett Study

To a clean, nitrogen-flushed NMR tube containing a septum was added hydrazide **S6** (0.0131 g, 0.05 mmol) followed by an equimolar amount (0.05 mmol) of one of the 4-substituted *N*-aryl-*N*-tosylhydrazides of interest (0.0170 g - **S10**, or 0.0148 g - **S13**, or 0.0144 g - **S15**, or 0.0146 g - **S16**, or 0.0159 g – **S19**). Then 2 mL of CD_3CN was added and an initial ^1H NMR spectrum of the mixture was recorded. Then enough Bobbitt's salt (**1**) to react with half the mixture (0.0015 g, 0.05 mmol) was added and the tube was capped and shaken for 1-2 minutes, then after 5 minutes and 10 minutes a ^1H NMR spectrum of each of the mixtures was collected. There were no differences observed in the 5- and 10-minute spectra, and a KI starch test of an aliquot verified that no **1** was remaining, thus the reactions were complete. The relative rate of oxidation of each of the hydrazides (k) by **1** versus *N*-phenyl tosyl hydrazide **S6** (k_0) was determined using the molar ratios obtained by the ^1H NMR integration of non-overlapping peaks (the aryl C-H's ortho to the diazene or the tosyl CH_3) of the two resulting diazene products (e.g., **6** versus **10** = 1 : 1.30, **6** versus **13** = 1 : 0.7, **6** versus **15** = 1 : 0.06, **6** versus **16** = 1 : 9.00, , **6** versus **19** = 1 : 0.4, and **6** versus **6** = 1). The logarithm these relative rates (log k/k_0) were then plotted versus the known σ_p values for the subsitutents.[11]

Computational details

All calculations were carried out using Gaussian-09 software at 298 K using the opt=tight and the integral=ultrafine keywords.[12] In the gas phase the B3LYP functional at the 6-311+G** level was used and the conductor-like polarizable continuum model (CPCM) was employed for acetonitrile or dichloromethane using the B3LYP functional with the addition of Grimme's D3 dispersion (B3LYP-D3) also at the 6-311+G** level.[13, 14, 15] All computed transition states were found to have one imaginary frequency and intrinsic reaction coordinate (IRC) analyses were conducted for verification.

Table S1 – B3LYP/6-311+G** Calculated Energies in Gas Phase for Figure 2.

	Energy 298K (hartrees)	Enthalpy 298K (hartrees)	Free Energy 298K (hartrees)	Energy 298K (kcal/mol)	Enthalpy 298K (kcal/mol)	Free Energy 298K (kcal/mol)
**Calculated Total Energies for 1st Oxidation**						
oxoammonium cation of **1**	-691.3411	-691.3401	-691.4027	-433823.4411	-433822.8487	-433862.1158
hydrazine **28**	-573.9020	-573.9010	-573.9520	-360129.2227	-360128.6297	-360160.6496
SM (**1** + **28**)	-1265.2430	-1265.2412	-1265.3548	-793952.6638	-793951.4784	-794022.7654
TS1	-1265.2545	-1265.2535	-1265.3473	-793959.8262	-793959.2332	-794018.0648
TS1 - SM				-7.16	-7.75	4.70
**Calculated Total Energies for 2nd Oxidation**						
protonated diazene **29**	-573.0592	-573.0582	-573.1089	-359600.3478	-359599.7555	-359631.5884
hydroxylamine **30**	-692.2039	-692.2029	-692.2664	-434364.8454	-434364.2531	-434404.0761
**29** + **30**	-1265.2630	-1265.2611	-1265.3753	-793965.1933	-793964.0085	-794035.6645
(**29** + **30**) – SM				-12.53	-12.53	-12.90
TS2	-1265.2622	-1265.2612	-1265.3534	-793964.6762	-793964.0838	-794021.9076
TS2 – SM				-12.01	-12.61	0.86
protonated hydroxylamine **31**	-692.5562	-692.5552	-692.6196	-434585.9329	-434585.3405	-434625.7214
diazene **7**	-572.7073	-572.7064	-572.7568	-359379.5553	-359378.9629	-359410.6189
(**31** + **7**) – SM				-12.82	-12.83	-13.57

Table S2 – B3LYP-D3/6-311+G** Calculated Energies with CPCM solvation model with CH_3CN and dispersion for Figure 2

	Energy 298K (hartrees)	Enthalpy 298K (hartrees)	Free Energy 298K (hartrees)	Energy 298K (kcal/mol)	Enthalpy 298K (kcal/mol)	Free Energy 298K (kcal/mol)
**Calculated Total Energies for 1st Oxidation**						
oxoammonium cation of **1**	-691.4589	-691.4580	-691.5199	-433897.3838	-433896.7914	-433935.6336
hydrazine **28**	-573.9306	-573.9297	-573.9805	-360147.2059	-360146.6135	-360178.5224
SM (**1** + **28**)	-1265.3895	-1265.3877	-1265.5004	-794044.5896	-794043.4049	-794114.1560
TS1	-1265.3968	-1265.3958	-1265.4875	-794049.1353	-794048.5423	-794106.0636
TS1 - SM				-4.55	-5.14	8.09
**Calculated Total Energies for 2nd Oxidation**						
protonated diazene **29**	-573.1435	-573.1426	-573.1929	-359653.2852	-359652.6928	-359684.2861
hydroxylamine **30**	-692.2598	-692.2589	-692.3205	-434399.9565	-434399.3641	-434438.0545
**29** + **30**	-1265.4033	-1265.4014	-1265.5134	-794053.2417	-794052.0570	-794122.3406
(**29** + **30**) – SM				-8.65	-8.65	-8.18
TS2	-1265.4115	-1265.4105	-1265.5009	-794058.3409	-794057.7485	-794114.4880
TS2 – SM				-13.75	-14.34	-0.33
protonated hydroxylamine **31**	-692.6833	-692.6824	-692.7453	-434665.7246	-434665.1322	-434704.5982
diazene **7**	-572.7284	-572.7275	-572.7782	-359392.8259	-359392.2335	-359424.0721
(**31** + **7**) – SM	-1265.4118	-1265.4099	-1265.5235	-794058.5505	-794057.3657	-794128.6703
(**31** + **7**) – SM				-13.96	-13.96	-14.51

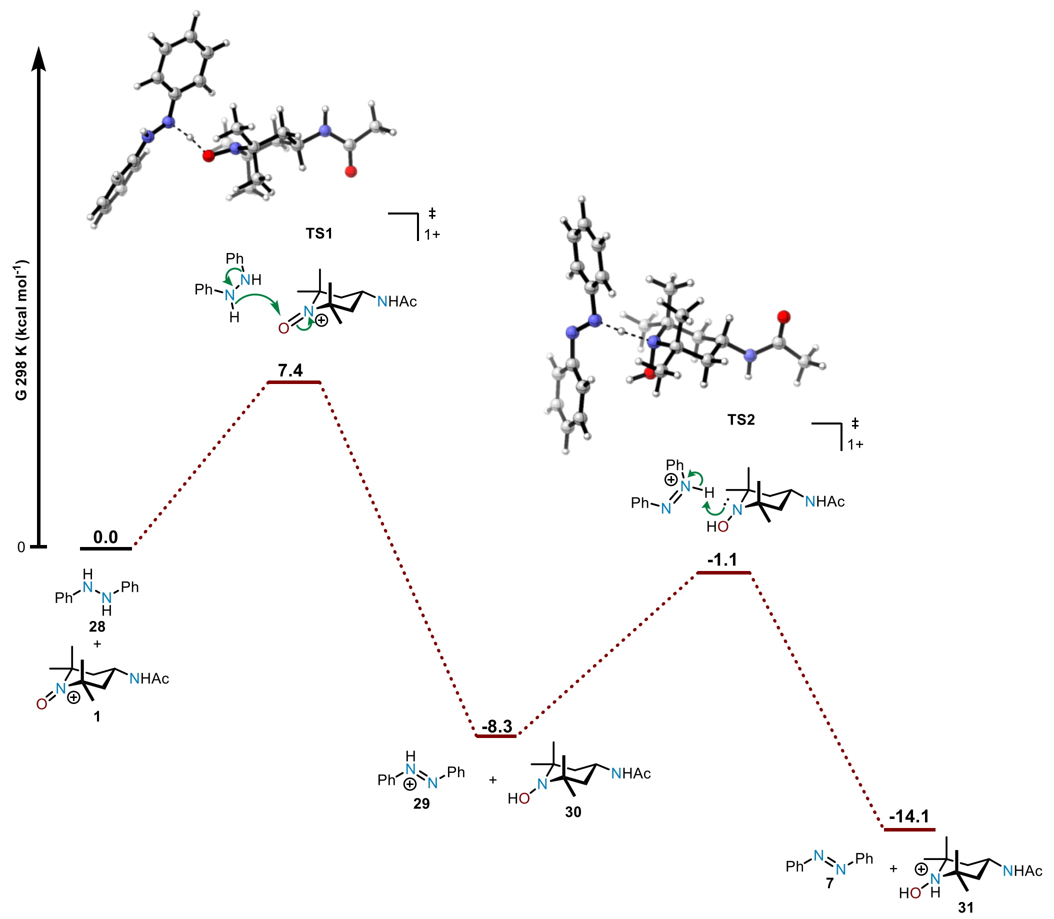

Figure S1. Reaction coordinate diagram for the oxidation of diphenyl hydrazine **28** by **1** to azobenzene **7** calculated at the B3LYP/6-311+G** level with CPCM solvation model in dichloromethane.

Table S3 – B3LYP-D3/6-311+G** Calculated Energies with CPCM solvation model with DCM and dispersion for Figure S1.

	Energy 298K (hartrees)	Enthalpy 298K (hartrees)	Free Energy 298K (hartrees)	Energy 298K (kcal/mol)	Enthalpy 298K (kcal/mol)	Free Energy 298K (kcal/mol)
**Calculated Total Energies for 1st Oxidation**						
oxoammonium cation of **1**	-691.4518	-691.4509	-691.5130	-433892.9454	-433892.3530	-433931.3528
hydrazine **28**	-573.9298	-573.9288	-573.9797	-360146.6681	-360146.0751	-360177.9903
SM (**1** + **28**)	-1265.3816	-1265.3797	-1265.4927	-794039.6135	-794038.4281	-794109.3430
TS1	-1265.3903	-1265.3894	-1265.4809	-794045.0665	-794044.4742	-794101.9378
TS1 - SM				-5.45	-6.05	7.41
**Calculated Total Energies for 2nd Oxidation**						
protonated diazene **29**	-573.1375	-573.1365	-573.1869	-359649.5013	-359648.9083	-359680.5097
hydroxylamine **30**	-692.2584	-692.2574	-692.3191	-434399.0636	-434398.4712	-434437.1365
**29** + **30**	-1265.3959	-1265.3940	-1265.5060	-794048.5649	-794047.3795	-794117.6462
(**29** + **30**) – SM				-8.95	-8.95	-8.30
TS2	-1265.4051	-1265.4042	-1265.4945	-794054.3788	-794053.7864	-794110.4619
TS2 – SM				-14.77	-15.36	-1.12
protonated hydroxylamine **31**	-692.6757	-692.6748	-692.7375	-434660.9392	-434660.3468	-434699.6942
diazene **7**	-572.7279	-572.7269	-572.7777	-359392.4764	-359391.8840	-359423.7069
(**31** + **7**) – SM	-1265.4036	-1265.4017	-1265.5151	-794053.4155	-794052.2308	-794123.4011
(**31** + **7**) – sm				-13.80	-13.80	-14.06

Table **S4** – B3LYP/6-311+G** Calculated Energies in Gas Phase for Figure 2

	Energy 298K (hartrees)	Enthalpy 298K (hartrees)	Free Energy 298K (hartrees)	Energy 298K (kcal/mol)	Enthalpy 298K (kcal/mol)	Free Energy 298K (kcal/mol)
**1**	-691.3411	-691.3401	-691.4027	-433823.4411	-433822.8487	-433862.1158
Hydrazide S**6**	-1161.8269	-1161.8260	-1161.8913	-729058.0219	-729057.4289	-729098.3864
TS of compounds **S6** & **1**	-1853.1635	-1853.1626	-1853.2708	-1162878.6300	-1162878.0370	-1162945.9280
(**1** + **S6**)						-1162960.5023
ΔΔG (TS1 – (**1** + **S6**))						14.57
Hydrazide **S12**	-1261.1013	-1261.1004	-1261.1677	-791353.6950	-791353.1026	-791395.3365
TS of compounds **S12** & **1**	-1952.4362	-1952.4352	-1952.5456	-1225173.2100	-1225172.6170	-1225241.8930
(**1** + **S12**)						-1225257.4517
ΔΔG (TS1 – (**1** + **S12**))						15.56
Hydrazide **S13**	-1621.4574	-1621.4564	-1621.5250	-1017480.7080	-1017480.1150	-1017523.1720
TS of compounds **S13** & **1**	-2312.7916	-2312.7907	-2312.9023	-1451299.8800	-1451299.2880	-1451369.3130
(**1** + **S13**)						-1451385.2880
ΔΔG (TS1 – (**1** + **S13**))						15.97
Hydrazide **S15**	-1254.0947	-1254.0937	-1254.1634	-786956.9445	-786956.3521	-787000.0783
TS of compounds **S15** & **1**	-1945.4187	-1945.4177	-1945.5305	-1220769.6630	-1220769.0700	-1220839.8370
(**1** + **S15**)						-1220862.1941
ΔΔG (TS1 – (**1** + **S15**))						22.36
Hydrazide **S21**	-1201.1249	-1201.1239	-1201.1943	-753717.8615	-753717.2692	-753761.4195
TS of compounds **S21** & **1**	-1892.4660	-1892.4650	-1892.5788	-1187541.3230	-1187540.7300	-1187612.1380
(**1** + **S21**)						-1187623.5353
ΔΔG (TS1 – (**1** + **S21**))						11.40
Hydrazide **S16**	-1276.3461	-1276.3451	-1276.4165	-800919.9255	-800919.3332	-800964.1035
TS of compounds **S16** & **1**	-1967.6932	-1967.6922	-1967.8064	-1234747.1320	-1234746.5390	-1234818.2030
(**1** + **S16**)						-1234826.2193
ΔΔG (TS1 – (**1** + **S16**))						8.02

Intrinsic reaction coordinate studies

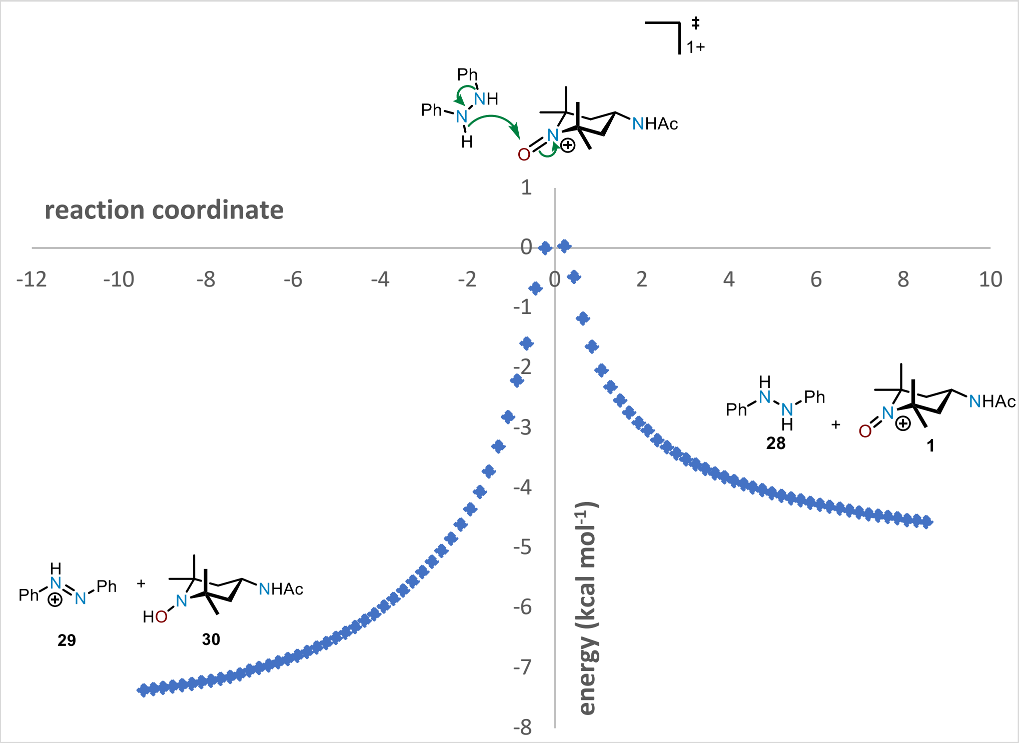

Figure S2. IRC for transition state between 1,2-diphenylhydrazine **28** and cation of Bobbitt's salt **1** in the gas phase.

IRC surface leading to reactants on right, products on left.

reaction coordinate	uncorrected energy (kcal mol^{-1})
-9.43466	-794316.45925
-9.21421	-794316.44139
-8.99369	-794316.42204
-8.77322	-794316.40107
-8.55275	-794316.37839
-8.33228	-794316.35386
-8.1118	-794316.32733
-7.89131	-794316.29864
-7.67083	-794316.26763
-7.45045	-794316.23407
-7.23006	-794316.19780
-7.00956	-794316.13647
-6.78904	-794316.09375
-6.56854	-794316.04744

-6.34803	-794315.99722
-6.12752	-794315.94271
-5.907	-794315.88354
-5.68648	-794315.81929
-5.46596	-794315.74954
-5.24543	-794315.67387
-5.0249	-794315.59183
-4.80437	-794315.50299
-4.58383	-794315.40690
-4.36329	-794315.30313
-4.14275	-794315.19126
-3.92222	-794315.07086
-3.70168	-794314.94145
-3.48114	-794314.80254
-3.26061	-794314.65350
-3.0401	-794314.49348
-2.81959	-794314.32121
-2.59911	-794314.13474
-2.37867	-794313.93094
-2.16005	-794313.70198
-1.94188	-794313.44909
-1.72408	-794313.15920
-1.50565	-794312.81794
-1.28942	-794312.40482
-1.07274	-794311.91559
-0.86265	-794311.30430
-0.64569	-794310.68330
-0.44041	-794309.76648
-0.22059	-794309.09921
0.22059	-794309.06481
0.4403	-794309.57201
0.64328	-794310.26728
0.85671	-794310.73911
1.06691	-794311.13087
1.28233	-794311.40735
1.49337	-794311.63297
1.70488	-794311.83907
1.91852	-794312.00670
2.13304	-794312.13904
2.34535	-794312.29292
2.56578	-794312.41188
2.78623	-794312.51799
3.00668	-794312.61323

3.22715	-794312.69928
3.44761	-794312.77741
3.66806	-794312.84870
3.88842	-794312.91391
4.10862	-794312.97373
4.32831	-794313.02849
4.54729	-794313.08166
4.76779	-794313.13075
4.98829	-794313.17696
5.2088	-794313.22059
5.42931	-794313.26191
5.64982	-794313.30114
5.87033	-794313.33841
6.09084	-794313.37386
6.31135	-794313.40757
6.53185	-794313.43963
6.75236	-794313.47009
6.97289	-794313.49900
7.1934	-794313.52640
7.41391	-794313.55235
7.63441	-794313.57688
7.85491	-794313.60003
8.07541	-794313.62185
8.29591	-794313.64239
8.51641	-794313.66169

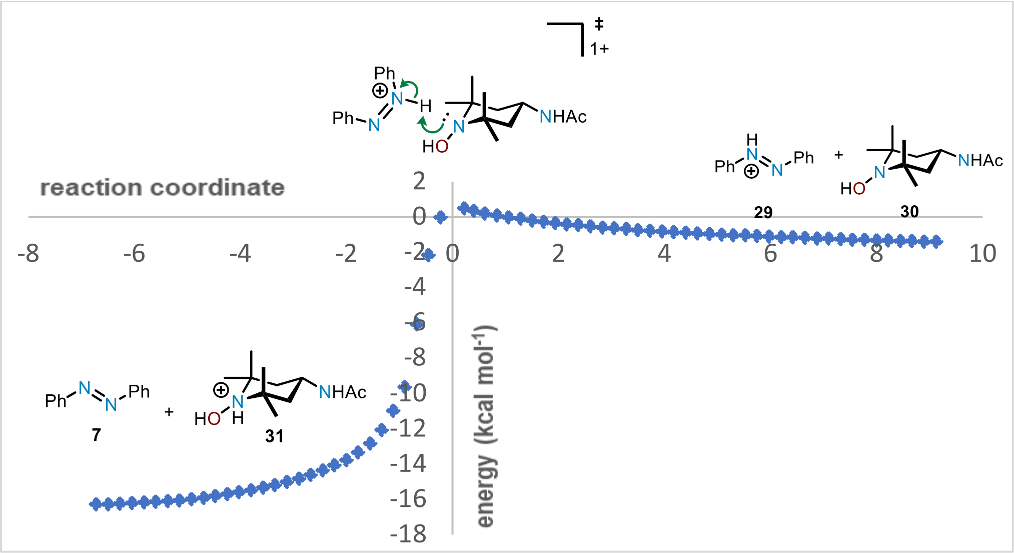

Figure S3. IRC for transition state between azobenzene **29** and hydroxylamine **30** in the gas phase.

IRC surface leading to reactants on right, products on left.

reaction coordinate	uncorrected energy (kcal mol^{-1})
-6.72077	-794331.04635
-6.49613	-794331.02717
-6.27153	-794331.00489
-6.04692	-794330.97837
-5.82226	-794330.94637
-5.59755	-794330.90760
-5.3728	-794330.86091
-5.14803	-794330.80530
-4.92323	-794330.73989
-4.69842	-794330.66388
-4.47361	-794330.57650
-4.2488	-794330.47698
-4.02399	-794330.36450
-3.79918	-794330.23820
-3.57436	-794330.09711
-3.34955	-794329.94015
-3.12475	-794329.76602
-2.89996	-794329.57307
-2.67521	-794329.35882
-2.45052	-794329.11887
-2.22631	-794328.84367

-2.00243	-794328.51932
-1.77827	-794328.11558
-1.55563	-794327.57657
-1.33587	-794326.83630
-1.12208	-794325.72600
-0.89875	-794324.41393
-0.67394	-794320.84926
-0.44916	-794316.91789
-0.22487	-794314.76634
0.22487	-794314.28748
0.40158	-794314.43082
0.61387	-794314.57219
0.83791	-794314.67532
1.05823	-794314.77487
1.28054	-794314.86036
1.4981	-794314.94540
1.71742	-794315.02133
1.93519	-794315.09721
2.15572	-794315.16261
2.37557	-794315.23195
2.60036	-794315.29194
2.82516	-794315.34756
3.04995	-794315.39930
3.27475	-794315.44757
3.49954	-794315.49280
3.72434	-794315.53538
3.94913	-794315.57566
4.17393	-794315.61394
4.39872	-794315.65046
4.62352	-794315.68542
4.84833	-794315.71897
5.07313	-794315.75125
5.29793	-794315.78236
5.52272	-794315.81235
5.74751	-794315.84129
5.97229	-794315.86921
6.19707	-794315.89613
6.42185	-794315.92209
6.64662	-794315.94708
6.87139	-794315.97115
7.09621	-794315.99427
7.32097	-794316.01650
7.54573	-794316.03786

7.77049	-794316.05839
7.99524	-794316.07814
8.21998	-794316.09717
8.44472	-794316.11554
8.66944	-794316.13328
8.89414	-794316.15046
9.11882	-794316.16714

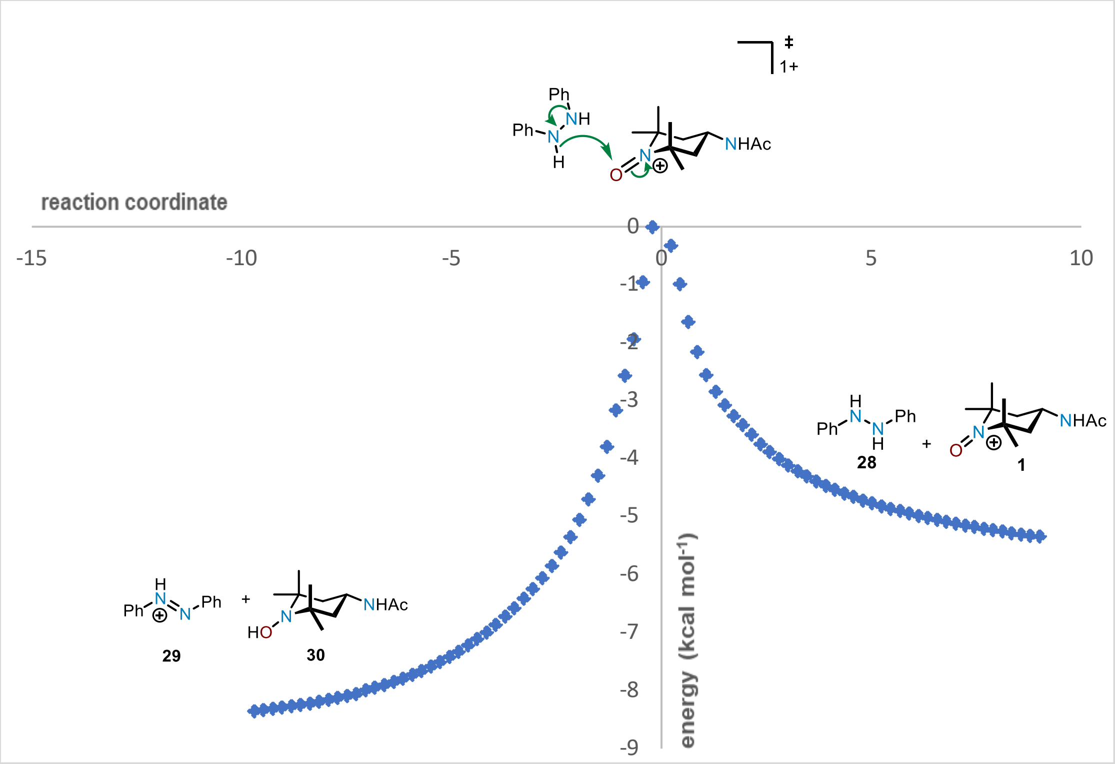

Figure S4. IRC for transition state between 1,2-diphenylhydrazine **28** and cation of Bobbitt's salt **1** using the CPCM solvation model with MeCN.

IRC surface leading to reactants on right, products on left.

reaction coordinate	uncorrected energy (kcal mol^{-1})
-9.70487	-794316.47711
-9.48336	-794316.45887
-9.26184	-794316.43916
-9.04028	-794316.41791
-8.81876	-794316.39502
-8.59724	-794316.37040
-8.37572	-794316.34396
-8.15419	-794316.31557
-7.93266	-794316.28510
-7.71113	-794316.25241
-7.4897	-794316.21725
-7.26826	-794316.17946
-7.04672	-794316.11616
-6.82516	-794316.07184
-6.60362	-794316.02378

-6.38207	-794315.97156
-6.16051	-794315.91471
-5.93896	-794315.85275
-5.71739	-794315.78520
-5.49582	-794315.71160
-5.27425	-794315.63147
-5.05267	-794315.54435
-4.8311	-794315.44980
-4.60951	-794315.34739
-4.38793	-794315.23666
-4.16635	-794315.11722
-3.94477	-794314.98857
-3.72319	-794314.85019
-3.50163	-794314.70139
-3.28007	-794314.54117
-3.05853	-794314.36805
-2.83702	-794314.17970
-2.61553	-794313.97261
-2.39407	-794313.74175
-2.17373	-794313.47831
-1.95288	-794313.18110
-1.73254	-794312.82996
-1.51214	-794312.41963
-1.29281	-794311.92401
-1.08319	-794311.28749
-0.87432	-794310.69616
-0.65317	-794310.06572
-0.43996	-794309.07863
-0.22164	-794308.12643
0.22164	-794308.44979
0.43798	-794309.11530
0.64326	-794309.76879
0.85473	-794310.29018
1.07197	-794310.67789
1.2922	-794310.97460
1.51009	-794311.20226
1.72412	-794311.39234
1.93509	-794311.54031
2.1436	-794311.71447
2.35882	-794311.87536
2.58031	-794312.00892
2.80184	-794312.12938
3.02338	-794312.23879

3.24492	-794312.33861
3.46647	-794312.42994
3.68803	-794312.51367
3.90958	-794312.59053
4.13113	-794312.66117
4.35268	-794312.72621
4.57423	-794312.78623
4.79578	-794312.84178
5.01732	-794312.89342
5.23886	-794312.94163
5.4604	-794312.98685
5.68194	-794313.02948
5.90347	-794313.06983
6.12501	-794313.10816
6.34655	-794313.14465
6.56809	-794313.17947
6.78963	-794313.21274
7.0112	-794313.24451
7.23274	-794313.27486
7.45428	-794313.30381
7.67582	-794313.33140
7.89736	-794313.35765
8.1189	-794313.38260
8.34044	-794313.40627
8.56197	-794313.42871
8.78351	-794313.44995
9.00505	-794313.47004

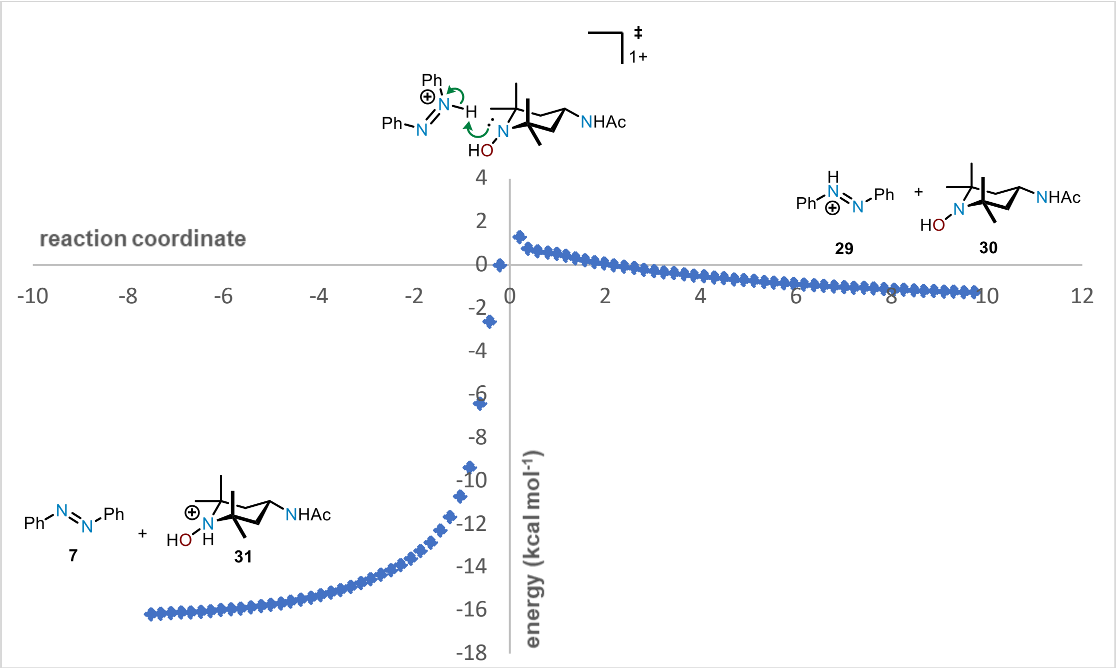

Figure S5. IRC for transition state between azobenzene **29** and hydroxylamine **30** using the CPCM solvation model with MeCN.

IRC surface leading to reactants on right, products on left.

reaction coordinate	uncorrected energy (kcal mol^{-1})
-7.52446	-794330.98642
-7.31498	-794330.96750
-7.10551	-794330.94680
-6.89603	-794330.92410
-6.68656	-794330.89912
-6.47709	-794330.87147
-6.26763	-794330.84072
-6.05816	-794330.80631
-5.84868	-794330.76765
-5.6392	-794330.72409
-5.42971	-794330.67497
-5.22021	-794330.61961
-5.01071	-794330.55731
-4.8012	-794330.48738
-4.59168	-794330.40906
-4.38217	-794330.32160
-4.17265	-794330.22414
-3.96313	-794330.11581

-3.75361	-794329.99559
-3.54409	-794329.86241
-3.33458	-794329.71505
-3.12506	-794329.55212
-2.91556	-794329.37200
-2.70607	-794329.17258
-2.49662	-794328.95071
-2.28721	-794328.70123
-2.07796	-794328.41471
-1.8693	-794328.07574
-1.66056	-794327.66358
-1.45749	-794327.11431
-1.25124	-794326.48488
-1.04592	-794325.53927
-0.83745	-794324.21834
-0.62793	-794321.23811
-0.41845	-794317.44662
-0.20958	-794314.82529
0.20958	-794313.52655
0.38641	-794314.04145
0.58759	-794314.17157
0.79107	-794314.23043
0.98329	-794314.30105
1.17507	-794314.38932
1.36943	-794314.49694
1.57435	-794314.57736
1.77499	-794314.66428
1.98023	-794314.73764
2.18397	-794314.81867
2.39348	-794314.88976
2.60299	-794314.95712
2.8125	-794315.02106
3.02202	-794315.08181
3.23154	-794315.13959
3.44106	-794315.19460
3.65058	-794315.24702
3.8601	-794315.29702
4.06962	-794315.34474
4.27914	-794315.39032
4.48867	-794315.43387
4.69819	-794315.47549
4.90771	-794315.51530
5.11722	-794315.55337

5.32674	-794315.58980
5.53626	-794315.62465
5.74578	-794315.65799
5.95529	-794315.68989
6.16481	-794315.72041
6.37432	-794315.74960
6.58386	-794315.77751
6.79337	-794315.80422
7.00288	-794315.82977
7.21239	-794315.85423
7.4219	-794315.87767
7.63141	-794315.90017
7.84091	-794315.92180
8.05041	-794315.94265
8.25991	-794315.96278
8.46941	-794315.98229
8.67893	-794316.00121
8.88842	-794316.01960
9.0979	-794316.03750
9.30738	-794316.05491
9.51686	-794316.07186
9.72633	-794316.08835

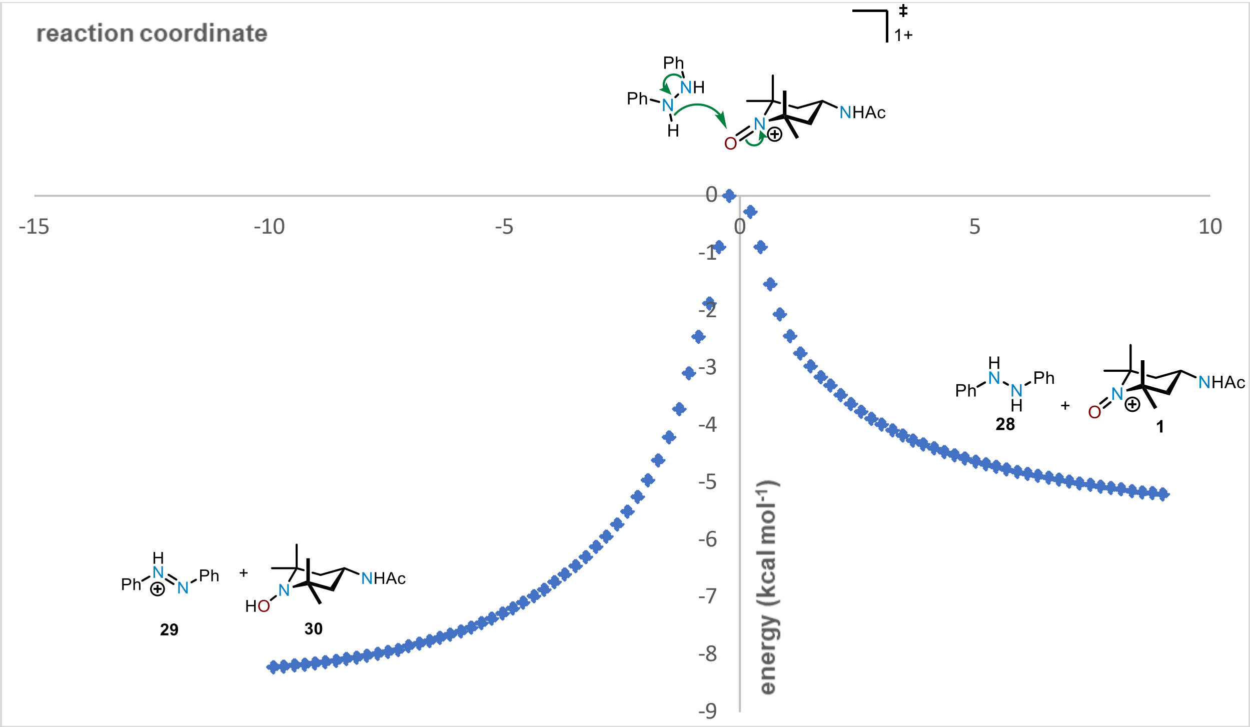

Figure S6. IRC for transition state between 1,2-diphenylhydrazine **28** and cation of Bobbitt's salt **1** using the CPCM solvation model with DCM.

IRC surface leading to reactants on right, products on left.

reaction coordinate	uncorrected energy (kcal mol^{-1})
-9.91709	-794316.50247
-9.69581	-794316.48594
-9.47452	-794316.46805
-9.25322	-794316.44870
-9.03187	-794316.42781
-8.81056	-794316.40526
-8.58925	-794316.38098
-8.36794	-794316.35486
-8.14663	-794316.32678
-7.92531	-794316.29662
-7.70399	-794316.26423
-7.48277	-794316.22938
-7.26154	-794316.19192
-7.04021	-794316.12903
-6.81886	-794316.08512
-6.59753	-794316.03754

-6.37619	-794315.98587
-6.15485	-794315.92967
-5.9335	-794315.86846
-5.71214	-794315.80176
-5.49079	-794315.72911
-5.26942	-794315.65004
-5.04806	-794315.56407
-4.82669	-794315.47077
-4.60532	-794315.36970
-4.38395	-794315.26042
-4.16258	-794315.14249
-3.9412	-794315.01547
-3.71984	-794314.87881
-3.49848	-794314.73187
-3.27713	-794314.57370
-3.0558	-794314.40287
-2.83449	-794314.21719
-2.61321	-794314.01326
-2.39196	-794313.78618
-2.17149	-794313.52779
-1.95066	-794313.23633
-1.73006	-794312.89250
-1.50953	-794312.48709
-1.28973	-794311.99824
-1.07894	-794311.37011
-0.87237	-794310.74076
-0.65164	-794310.15477
-0.43982	-794309.17194
-0.22143	-794308.28493
0.22143	-794308.56389
0.43794	-794309.17866
0.64287	-794309.82600
0.85408	-794310.34373
1.0706	-794310.73050
1.29046	-794311.02438
1.50764	-794311.24832
1.72089	-794311.43791
1.93153	-794311.58687
2.14029	-794311.75650
2.35511	-794311.91571
2.57639	-794312.04696
2.7977	-794312.16520
3.01903	-794312.27246

3.24037	-794312.37025
3.46171	-794312.45967
3.68305	-794312.54165
3.90439	-794312.61693
4.12573	-794312.68617
4.34707	-794312.75000
4.5684	-794312.80902
4.78974	-794312.86375
5.01108	-794312.91473
5.23241	-794312.96243
5.45374	-794313.00727
5.67507	-794313.04960
5.8964	-794313.08971
6.11773	-794313.12783
6.33907	-794313.16414
6.5604	-794313.19878
6.78173	-794313.23187
7.00309	-794313.26345
7.22443	-794313.29359
7.44576	-794313.32233
7.66709	-794313.34970
7.88842	-794313.37574
8.10975	-794313.40048
8.33108	-794313.42396
8.55241	-794313.44623
8.77374	-794313.46732
8.99506	-794313.48729

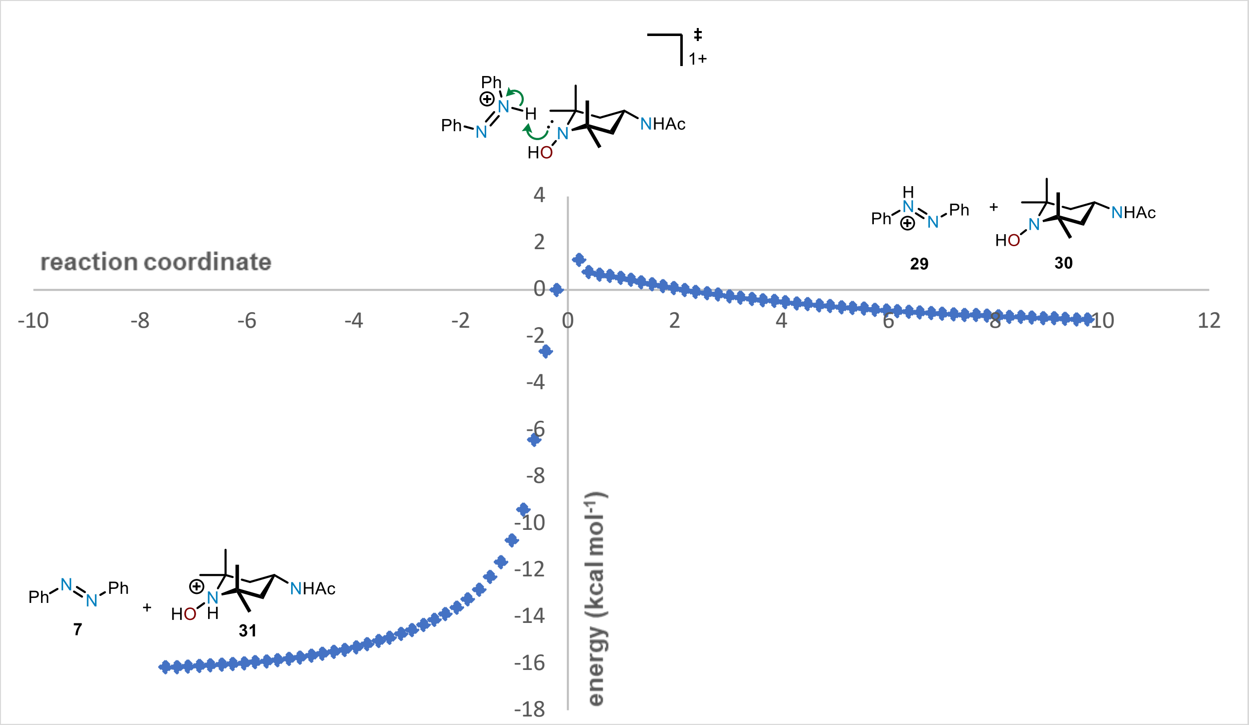

Figure S7. IRC for transition state between azobenzene **29** and hydroxylamine **30** using the CPCM solvation model with DCM.

IRC surface leading to reactants on right, products on left.

reaction coordinate	uncorrected energy (kcal mol^{-1})
-7.52502	-794330.98646
-7.31553	-794330.96754
-7.10603	-794330.94684
-6.89654	-794330.92414
-6.68706	-794330.89916
-6.47757	-794330.87151
-6.26809	-794330.84075
-6.05861	-794330.80635
-5.84912	-794330.76769
-5.63962	-794330.72413
-5.43011	-794330.67501
-5.2206	-794330.61965
-5.01107	-794330.55735
-4.80155	-794330.48741
-4.59202	-794330.40910
-4.38249	-794330.32163

-4.17295	-794330.22417
-3.96342	-794330.11582
-3.75388	-794329.99559
-3.54435	-794329.86240
-3.33482	-794329.71503
-3.12529	-794329.55209
-2.91576	-794329.37195
-2.70626	-794329.17251
-2.49679	-794328.95062
-2.28738	-794328.70110
-2.07811	-794328.41456
-1.86943	-794328.07554
-1.66068	-794327.66334
-1.45761	-794327.11396
-1.25135	-794326.48474
-1.046	-794325.53924
-0.83751	-794324.21814
-0.62798	-794321.23843
-0.41848	-794317.44680
-0.2096	-794314.82511
0.2096	-794313.52681
0.38643	-794314.04158
0.58765	-794314.17181
0.79123	-794314.23079
0.98353	-794314.30040
1.17519	-794314.38892
1.36949	-794314.49669
1.57437	-794314.57708
1.775	-794314.66409
1.98031	-794314.73775
2.18418	-794314.81859
2.39371	-794314.88968
2.60324	-794314.95705
2.81276	-794315.02099
3.0223	-794315.08175
3.23183	-794315.13954
3.44136	-794315.19456
3.6509	-794315.24699
3.86044	-794315.29699
4.06997	-794315.34471
4.27951	-794315.39030
4.48905	-794315.43385
4.69859	-794315.47548

4.90812	-794315.51529
5.11766	-794315.55337
5.32719	-794315.58980
5.53672	-794315.62465
5.74626	-794315.65799
5.95579	-794315.68990
6.16532	-794315.72041
6.37485	-794315.74961
6.5844	-794315.77752
6.79393	-794315.80423
7.00345	-794315.82978
7.21298	-794315.85424
7.4225	-794315.87768
7.63203	-794315.90019
7.84155	-794315.92182
8.05107	-794315.94266
8.26058	-794315.96280
8.47009	-794315.98231
8.67963	-794316.00122
8.88914	-794316.01962
9.09864	-794316.03752
9.30813	-794316.05493
9.51762	-794316.07188
9.72711	-794316.08837

Cartesian Coordinates of Computed Compounds

Oxoammonium cation of **1**

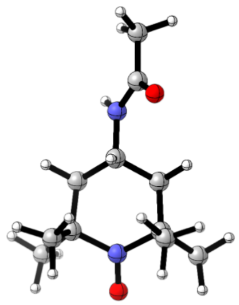

in gas phase

C	0.07749100	-1.48498300	-0.42697100
C	1.51431500	-1.28828400	0.09877800
C	1.01287700	1.39602700	-0.10934900
C	-0.36840100	0.96632600	-0.63408000
C	-0.88131800	-0.35838200	-0.05151400
H	0.11424300	-1.59135100	-1.51721100
H	-0.27472500	-2.43829100	-0.02432000
H	-1.06671700	1.76611900	-0.38016100
H	-0.33461500	0.89277100	-1.72625900
H	-0.95153300	-0.26283800	1.03421900
O	3.10770700	0.36702500	-0.38052500
N	1.95846200	0.17365800	-0.16038200
N	-2.22981300	-0.65500000	-0.50261600
H	-2.37793800	-1.25273000	-1.30160100
C	-3.30488900	-0.03986900	0.10514100
O	-3.12750000	0.74912800	1.01861000
C	-4.67361800	-0.40831000	-0.41615800
H	-4.64995500	-1.11001300	-1.25152800
H	-5.24961000	-0.84677100	0.40140300
H	-5.18945400	0.50275700	-0.72493200
C	1.63585200	2.49297900	-0.97562700
H	2.57274900	2.86613400	-0.56363600
H	1.80812700	2.15084100	-1.99830400
H	0.92519600	3.32049300	-1.01155700
C	0.97602000	1.84646500	1.37435900
H	1.98282800	2.01816000	1.75882700
H	0.43651000	2.79534600	1.39240900
H	0.44626000	1.15502200	2.02499200
C	2.50635300	-2.20244700	-0.62458900
H	3.50714800	-2.14786200	-0.19838600
H	2.14559300	-3.22695100	-0.51631800
H	2.56190500	-1.97453000	-1.69112700
C	1.62146000	-1.49358700	1.63121900
H	1.47345400	-2.55997800	1.81310900
H	2.61404700	-1.22393200	1.99563800
H	0.86742600	-0.94783200	2.19381100

SCRF = (CPCM, solvent=acetonitrile)

C	-0.14018400	-1.25339900	-0.53873000
C	1.29569600	-1.36288000	0.01159100
C	1.29570400	1.36287800	0.01159000
C	-0.14017900	1.25340500	-0.53872800
C	-0.90003300	0.00000500	-0.09153600
H	-0.10196300	-1.28142900	-1.63182100
H	-0.66883600	-2.15108600	-0.21150300
H	-0.66882500	2.15109400	-0.21149700
H	-0.10195900	1.28143900	-1.63181900
H	-1.03396700	0.00000400	0.99026700
O	3.15165800	-0.00000700	-0.44077200
N	1.99895300	-0.00000300	-0.16838500
N	-2.24760400	0.00000700	-0.64107900
H	-2.35274600	0.00000100	-1.64562500
C	-3.37212300	0.00000400	0.12687900
O	-3.33373600	0.00000900	1.35700300
C	-4.68341400	-0.00000500	-0.62840000
H	-4.56072600	0.00000600	-1.71183900
H	-5.25646100	-0.88172300	-0.33394700
H	-5.25648500	0.88169100	-0.33393000
C	2.11073100	2.40378000	-0.75630900
H	3.09242400	2.56328800	-0.31205300
H	2.23018400	2.12405400	-1.80434700
H	1.55786600	3.34315100	-0.71216900
C	1.33030300	1.67504700	1.52819300
H	2.34328700	1.59481800	1.92332300
H	1.00197400	2.71052400	1.63148000
H	0.66136200	1.04963600	2.11348700
C	2.11072000	-2.40378900	-0.75630300
H	3.09241000	-2.56330100	-0.31204300
H	1.55784800	-3.34315600	-0.71216400
H	2.23017900	-2.12406500	-1.80434100
C	1.33028700	-1.67504800	1.52819500
H	1.00195800	-2.71052500	1.63148000
H	2.34327000	-1.59482100	1.92333100
H	0.66134400	-1.04963700	2.11348500

SCRF=(CPCM, solvent=dichloromethane)

C	-0.13898700	-1.25315300	-0.54666900
C	1.29429600	-1.36335400	0.01033800
C	1.29429500	1.36335400	0.01033200
C	-0.13899000	1.25315000	-0.54667000
C	-0.90034600	-0.00000200	-0.10146800
H	-0.09586700	-1.28084700	-1.63979900
H	-0.66970000	-2.15079800	-0.22252400
H	-0.66970400	2.15079400	-0.22252300
H	-0.09587400	1.28084600	-1.63980000
H	-1.03514200	-0.00000200	0.98037700
O	3.15390500	-0.00000100	-0.42611900
N	1.99864200	0.00000000	-0.16402700
N	-2.24815700	-0.00000600	-0.64853200
H	-2.35824800	-0.00001900	-1.65240700
C	-3.36943200	0.00000300	0.12841400
O	-3.31948500	0.00001900	1.35651700
C	-4.68582600	-0.00001000	-0.61750900
H	-4.57136100	-0.00001700	-1.70196900
H	-5.25688100	-0.88141800	-0.31849300
H	-5.25689200	0.88139600	-0.31850600
C	2.11357500	2.40343200	-0.75433500
H	3.09334600	2.56327700	-0.30597500
H	2.23814600	2.12343300	-1.80177900
H	1.56094500	3.34316000	-0.71364000
C	1.32125700	1.67685200	1.52701800
H	2.33241600	1.59813000	1.92735900
H	0.99182400	2.71225000	1.62810300
H	0.64976600	1.05157600	2.10961400
C	2.11357900	-2.40343600	-0.75432100
H	3.09334900	-2.56327700	-0.30595800
H	1.56095100	-3.34316400	-0.71362100
H	2.23815200	-2.12344300	-1.80176700
C	1.32125100	-1.67684500	1.52702500
H	0.99182400	-2.71224400	1.62811300
H	2.33240700	-1.59811400	1.92737400
H	0.64975200	-1.05157100	2.10961400

Hydroxylamine **30**

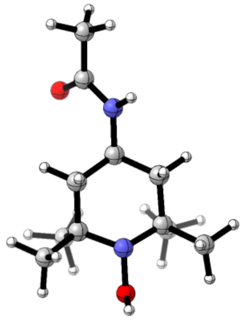

In gas phase

H	3.80626700	-0.00004500	-0.71405100
C	1.27858000	1.29417400	-0.04384600
C	1.27853000	-1.29418200	-0.04388100
C	-0.17903100	-1.24908500	-0.54819400
C	-0.94099200	0.00004000	-0.09741600
C	-0.17898300	1.24914800	-0.54816000
H	-0.17020800	-1.27738900	-1.64504400
H	-0.69792300	-2.14819400	-0.20383700
H	-1.05984600	0.00002500	0.98625400
H	-0.17015800	1.27748200	-1.64500900
H	-0.69783900	2.14826800	-0.20377800
N	1.91564800	-0.00001100	-0.42965300
O	3.26738800	-0.00004400	0.08388100
C	2.02136800	2.40149000	-0.81479800
H	1.49908900	3.35506500	-0.69858200
H	3.03759100	2.52734700	-0.43687200
H	2.06967400	2.15906900	-1.87949300
C	1.33872600	1.63127900	1.46341000
H	2.35067600	1.48215600	1.84233200
H	1.07263900	2.68159300	1.61159500
H	0.65333700	1.03552200	2.06507300
C	2.02127500	-2.40150600	-0.81486300
H	3.03749400	-2.52741200	-0.43694000
H	1.49896000	-3.35506400	-0.69867300
H	2.06959100	-2.15905900	-1.87955200
C	1.33866200	-1.63133000	1.46336500
H	1.07253600	-2.68163900	1.61152200
H	2.35061800	-1.48225600	1.84229300
H	0.65329500	-1.03556500	2.06504400
N	-2.30538800	0.00007800	-0.61984000
C	-3.41965600	0.00000100	0.17280000
C	-4.74760100	0.00004800	-0.56563900
H	-4.64796200	0.00014000	-1.65330300
H	-5.31630400	-0.88029200	-0.25946900
H	-5.31632400	0.88032400	-0.25932200
O	-3.36777000	-0.00009700	1.39368800
H	-2.42923200	0.00014500	-1.62097600

SCRF = (CPCM, solvent=acetonitrile)

H	3.80599100	-0.00000100	-0.73938200
C	1.28051300	1.29521700	-0.04234400
C	1.28051200	-1.29521800	-0.04234400
C	-0.18189500	-1.24980300	-0.53344500
C	-0.93906100	0.00000000	-0.07580400
C	-0.18189500	1.24980400	-0.53344500
H	-0.18671800	-1.27753200	-1.62926400
H	-0.69519600	-2.15011200	-0.18430400
H	-1.04902000	0.00000000	1.00831200
H	-0.18671700	1.27753300	-1.62926400
H	-0.69519500	2.15011300	-0.18430300
N	1.91542700	0.00000000	-0.43159900
O	3.27407300	0.00000000	0.06433000
C	2.01311800	2.40394900	-0.82097400
H	1.49388400	3.35709900	-0.69040100
H	3.03685200	2.52513800	-0.46220600
H	2.04226300	2.16781000	-1.88765600
C	1.35177200	1.63571300	1.46383300
H	2.36925900	1.50372900	1.83474200
H	1.07139700	2.68232000	1.61062800
H	0.68224800	1.03037800	2.07391900
C	2.01311700	-2.40394900	-0.82097400
H	3.03685100	-2.52513900	-0.46220700
H	1.49388200	-3.35709900	-0.69040200
H	2.04226200	-2.16780900	-1.88765700
C	1.35177200	-1.63571400	1.46383200
H	1.07139600	-2.68232000	1.61062700
H	2.36925800	-1.50373000	1.83474100
H	0.68224800	-1.03037900	2.07391900
N	-2.30434500	0.00000100	-0.60084400
C	-3.42297800	0.00000000	0.16389200
C	-4.73814300	0.00000100	-0.58962500
H	-4.61823300	0.00000100	-1.67369800
H	-5.31156300	-0.88157100	-0.29485600
H	-5.31156200	0.88157200	-0.29485500
O	-3.39461800	-0.00000100	1.39811900
H	-2.41779500	0.00000200	-1.60452900

SCRF = (CPCM, solvent= dichloromethane)

H	3.80597000	-0.00000300	-0.73765000
C	1.28037200	1.29509000	-0.04243300
C	1.28036900	-1.29509100	-0.04243600
C	-0.18166200	-1.24975000	-0.53455100
C	-0.93934500	0.00000300	-0.07767800
C	-0.18165900	1.24975400	-0.53454900
H	-0.18530700	-1.27767700	-1.63050700
H	-0.69544200	-2.14988400	-0.18561600
H	-1.05038300	0.00000200	1.00636200
H	-0.18530400	1.27768300	-1.63050400
H	-0.69543600	2.14988900	-0.18561200
N	1.91541500	-0.00000100	-0.43172500
O	3.27350900	-0.00000300	0.06558300
C	2.01383700	2.40379300	-0.82030200
H	1.49431600	3.35696500	-0.69084300
H	3.03690800	2.52538500	-0.45980600
H	2.04470700	2.16723700	-1.88687100
C	1.35084200	1.63502000	1.46387100
H	2.36777700	1.50089100	1.83544100
H	1.07228000	2.68208700	1.61094800
H	0.67956900	1.03083400	2.07312900
C	2.01383100	-2.40379300	-0.82030600
H	3.03690200	-2.52538900	-0.45981000
H	1.49430800	-3.35696500	-0.69085000
H	2.04470200	-2.16723600	-1.88687500
C	1.35083800	-1.63502300	1.46386800
H	1.07227400	-2.68209000	1.61094300
H	2.36777300	-1.50089800	1.83543800
H	0.67956600	-1.03083800	2.07312700
N	-2.30444900	0.00000500	-0.60270700
C	-3.42285800	0.00000000	0.16481000
C	-4.73921300	0.00000400	-0.58745100
H	-4.62115600	0.00000800	-1.67189500
H	-5.31220800	-0.88141000	-0.29153400
H	-5.31220800	0.88141500	-0.29152600
O	-3.39222000	-0.00000700	1.39749200
H	-2.41879900	0.00001000	-1.60612300

Protonated hydroxylamine **31**

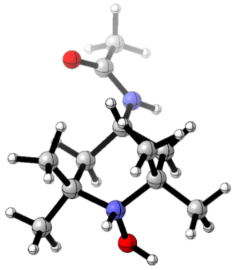

In gas phase

C	0.27347700	1.08478900	-0.54674000
C	-1.09119300	1.41233500	0.07051100
C	-1.45258100	-1.27846200	0.24830200
C	-0.05538000	-1.41433600	-0.37668400
C	0.88053300	-0.24271000	-0.05763300
H	0.18533600	1.06741400	-1.63716400
H	0.95047800	1.90275000	-0.28972000
H	0.37878200	-2.34678400	-0.00695100
H	-0.15821700	-1.51756600	-1.46232700
H	1.05224400	-0.18015100	1.01805900
C	-2.42205300	-2.32576000	-0.32426600
H	-2.11361900	-3.30678700	0.03937700
H	-3.45055100	-2.17406500	0.02155800
H	-2.39887000	-2.38964300	-1.41478100
C	-1.45876500	-1.41033900	1.77864900
H	-2.42604200	-1.13426900	2.20908100
H	-1.29248900	-2.46106800	2.02102000
H	-0.68026700	-0.84068500	2.27504600
C	-1.02212300	1.72852000	1.57214600
H	-0.63207800	2.74256400	1.66965500
H	-2.01071100	1.72477700	2.04090500
H	-0.35766200	1.08230500	2.13737000
C	-1.77554700	2.58526100	-0.64381500
H	-2.77830800	2.77193500	-0.24809900
H	-1.17808100	3.47784700	-0.45023900
H	-1.84063000	2.44501000	-1.71953400
N	-2.01244000	0.13930300	-0.13982500
O	-2.38686500	0.17317800	-1.50726000
H	-2.98857000	-0.57424700	-1.64582000
N	2.20015700	-0.46883600	-0.61832300
C	3.32990200	-0.06312500	0.06550900
C	4.65036200	-0.31034300	-0.62685000
H	4.55143600	-0.78504100	-1.60459600
H	5.17067600	0.64267700	-0.74064200
H	5.26667300	-0.94192900	0.01595900
O	3.24523000	0.45267500	1.16680500
H	2.29123200	-0.80561200	-1.56515800
H	-2.86150000	0.30548700	0.41290200

SCRF = (CPCM, solvent=acetonitrile)

C	0.10668200	1.27048000	-0.43550900
C	-1.30352100	1.35417400	0.16521300
C	-1.29547200	-1.35149400	0.17001900
C	0.11658000	-1.25685700	-0.42533700
C	0.88716900	0.01151000	-0.02691400
H	0.03519100	1.31377100	-1.52638200
H	0.65018100	2.16194700	-0.11468200
H	0.66810000	-2.14116200	-0.09865700
H	0.04696400	-1.30626300	-1.51599300
H	1.07416800	0.01640200	1.04665900
C	-2.09349600	-2.48083700	-0.49543200
H	-1.62020300	-3.42538300	-0.22416000
H	-3.12432800	-2.51990300	-0.13225100
H	-2.08809700	-2.41448200	-1.58240200
C	-1.30749100	-1.57961600	1.68886200
H	-2.29873700	-1.41610000	2.11834700
H	-1.05017700	-2.62557700	1.86083900
H	-0.58590500	-0.97879600	2.23313000
C	-1.30345900	1.57296800	1.68547000
H	-1.03155800	2.61504800	1.85826600
H	-2.29467500	1.42138700	2.11922800
H	-0.58687600	0.96214100	2.22480700
C	-2.12205600	2.47782400	-0.48468900
H	-3.15125100	2.48565100	-0.11639200
H	-1.66197100	3.42573300	-0.20113000
H	-2.13305600	2.40998500	-1.56930800
N	-2.03078500	-0.00217900	-0.16249900
O	-2.32283200	0.04399700	-1.55153800
H	-3.14007800	-0.46108400	-1.68304800
N	2.20974200	0.01353000	-0.63858900
C	3.36967000	-0.00011800	0.07127100
C	4.64247300	0.00000000	-0.74805300
H	4.46605400	0.01300700	-1.82399000
H	5.23604300	0.87440400	-0.47300100
H	5.22339100	-0.88872600	-0.49243100
O	3.39584100	-0.01170000	1.30275600
H	2.26748000	0.01905200	-1.64692600
H	-2.91991700	0.01292300	0.34794200

SCRF = (CPCM, solvent= dichloromethane)

C	0.10696300	1.27028200	-0.44040500
C	-1.30121000	1.35589500	0.16460600
C	-1.29418400	-1.35062000	0.16881900
C	0.11602500	-1.25703800	-0.43072300
C	0.88807400	0.01105600	-0.03391200
H	0.03248900	1.31431100	-1.53101700
H	0.65225600	2.16099100	-0.12039000
H	0.66812900	-2.14161000	-0.10553600
H	0.04423900	-1.30621300	-1.52130000
H	1.07677700	0.01524800	1.03949200
C	-2.09802100	-2.47877000	-0.49394200
H	-1.64644200	-3.42706800	-0.20056800
H	-3.13622800	-2.49764700	-0.14958600
H	-2.06827900	-2.43758200	-1.58288100
C	-1.30299800	-1.57944000	1.68753400
H	-2.29586700	-1.42675300	2.11773400
H	-1.03371800	-2.62219000	1.86033200
H	-0.58796600	-0.97042300	2.23101600
C	-1.29586400	1.57628600	1.68463800
H	-1.02784000	2.61968300	1.85534000
H	-2.28452700	1.42161300	2.12341900
H	-0.57386700	0.96962400	2.22160300
C	-2.12176700	2.47948300	-0.48358500
H	-3.14965200	2.48868100	-0.11161800
H	-1.65973000	3.42706300	-0.20210900
H	-2.13725300	2.41215900	-1.56815200
N	-2.03161900	-0.00102900	-0.15724300
O	-2.34098900	0.05847900	-1.54067400
H	-3.03298400	-0.60223800	-1.69908500
N	2.21007200	0.01321000	-0.64488900
C	3.36849600	-0.00309000	0.07191100
C	4.64520800	0.00354300	-0.74100600
H	4.47466200	0.01728700	-1.81801600
H	5.23340700	0.88013000	-0.46148100
H	5.22906100	-0.88245800	-0.48304300
O	3.38545300	-0.02139800	1.30178900
H	2.27097600	0.02586300	-1.65284100
H	-2.91685300	0.01410000	0.36107900

Hydrazine **28**

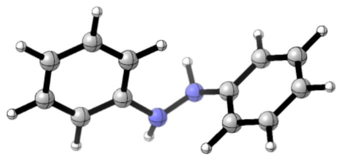

In gas phase

C	3.01374300	0.08810400	1.52711200
C	1.86223600	-0.49289000	1.00088200
C	1.63765900	-0.46654300	-0.37982100
C	2.57987700	0.15018100	-1.21604000
C	3.72506800	0.72604000	-0.67712000
C	3.95303900	0.69990400	0.69903500
H	3.17361200	0.06213200	2.59960500
H	1.13222400	-0.96262500	1.64680900
H	2.41538700	0.17000600	-2.28927600
H	4.44290100	1.19872200	-1.33854100
H	4.84558800	1.15005100	1.11684400
N	0.51982700	-1.08970200	-0.96700700
H	0.26967700	-0.72521300	-1.88114700
N	-0.57290000	-1.41203100	-0.16613700
H	-0.82848400	-2.38531700	-0.26092000
C	-1.66729900	-0.53138200	-0.07819100
C	-1.53127700	0.84272200	-0.31191300
C	-2.92151500	-1.04089800	0.29235900
C	-2.63556100	1.68300000	-0.17722500
H	-0.56655800	1.25585100	-0.57762400
C	-4.01237300	-0.19113000	0.42755000
H	-3.03542600	-2.10526600	0.47451500
C	-3.87972000	1.17842500	0.19217700
H	-2.51383600	2.74514600	-0.35913300
H	-4.97413900	-0.60290200	0.71299200
H	-4.73269600	1.83835200	0.29505600

SCRF = (CPCM, solvent=acetonitrile)

C	3.01792900	-0.02311300	1.53395800
C	1.86603100	-0.56661300	0.96696300
C	1.63532400	-0.43736100	-0.40928000
C	2.57485500	0.24663100	-1.19863000
C	3.72056300	0.78268500	-0.61946900
C	3.95429900	0.65290500	0.75166500
H	3.18084300	-0.13110500	2.60098400
H	1.14249600	-1.08898000	1.57921600
H	2.40342000	0.35370100	-2.26508400
H	4.43376600	1.30809400	-1.24527800
H	4.84697700	1.07289500	1.19983200
N	0.52351800	-1.02154000	-1.03652900
H	0.28595500	-0.62740200	-1.94156200
N	-0.57209100	-1.39841000	-0.26295300
H	-0.82705700	-2.36436000	-0.41972800
C	-1.66585100	-0.52618000	-0.12087500
C	-1.54498200	0.85733200	-0.30945100
C	-2.90845900	-1.05748000	0.26441200
C	-2.65005600	1.68650000	-0.11305000
H	-0.59524300	1.28669400	-0.60177300
C	-4.00020400	-0.21928700	0.45938600
H	-3.01118900	-2.12847000	0.40738900
C	-3.88166700	1.16078800	0.27262800
H	-2.53953100	2.75483500	-0.26368800
H	-4.95178300	-0.64808400	0.75396700
H	-4.73533000	1.81098600	0.42254900

SCRF = (CPCM, solvent= dichloromethane)

C	3.01727000	-0.00978300	1.53369100
C	1.86547200	-0.55798500	0.97134000
C	1.63553900	-0.44098900	-0.40584800
C	2.57538700	0.23523900	-1.20104300
C	3.72102400	0.77623400	-0.62647300
C	3.95401100	0.65880700	0.74569900
H	3.17975100	-0.10799300	2.60170200
H	1.14120000	-1.07419400	1.58788000
H	2.40479000	0.33205700	-2.26866200
H	4.43477800	1.29554100	-1.25671200
H	4.84662500	1.08254300	1.19040900
N	0.52315000	-1.03022100	-1.02859700
H	0.28398700	-0.63922500	-1.93459100
N	-0.57225500	-1.40056400	-0.25156600
H	-0.82736500	-2.36758600	-0.40089500
C	-1.66597000	-0.52703400	-0.11596300
C	-1.54325200	0.85540000	-0.31033600
C	-2.90999400	-1.05546600	0.26808700
C	-2.64815100	1.68624600	-0.12111000
H	-0.59170600	1.28264800	-0.59988400
C	-4.00156100	-0.21555700	0.45607700
H	-3.01405100	-2.12567700	0.41614900
C	-3.88127500	1.16333600	0.26315800
H	-2.53618600	2.75385200	-0.27567700
H	-4.95432700	-0.64210100	0.75005700
H	-4.73476400	1.81494700	0.40769100

Protonated Diazene **29**

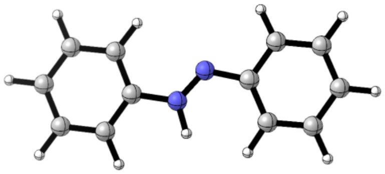

In gas phase

C	3.69393700	1.31811400	-0.00000800
C	2.31954500	1.14231200	-0.00000500
C	1.81706800	-0.16498700	-0.00000100
C	2.66349900	-1.28153800	0.00000100
C	4.03764200	-1.08442200	-0.00000100
C	4.55271400	0.21215000	-0.00000600
H	4.10371800	2.32049900	-0.00001100
H	1.64312000	1.98609500	-0.00000700
H	2.25419300	-2.28693500	0.00000400
H	4.70380300	-1.93778800	0.00000000
H	5.62516300	0.36490100	-0.00000700
N	0.42947500	-0.41730900	0.00000100
N	-0.45374300	0.48577500	-0.00000200
C	-1.79403000	0.16777100	0.00000100
C	-2.34437300	-1.13709700	0.00001000
C	-2.64525600	1.29452000	-0.00000400
C	-3.71612400	-1.29008200	0.00001100
H	-1.72485600	-2.02757400	0.00001500
C	-4.02119300	1.12450600	-0.00000300
H	-2.19500800	2.27930300	-0.00001100
C	-4.55570900	-0.16321900	0.00000500
H	-4.14834800	-2.28298900	0.00001800
H	-4.67495700	1.98746200	-0.00000700
H	-5.63055500	-0.30127700	0.00000700
H	0.16728800	-1.40912100	0.00000500

SCRF = (CPCM, solvent=acetonitrile)

C	3.68371500	1.32138400	0.03121200
C	2.30971800	1.13714100	0.02719200
C	1.81353000	-0.17155500	-0.00480500
C	2.66415700	-1.28264700	-0.03175400
C	4.03842400	-1.07700100	-0.02694600
C	4.54849200	0.22074000	0.00443300
H	4.08698100	2.32599200	0.05616400
H	1.62913800	1.97701500	0.04877300
H	2.25703700	-2.28717600	-0.05579000
H	4.70727900	-1.92792500	-0.04739000
H	5.62006700	0.37888400	0.00866400
N	0.42650200	-0.42796300	-0.00993200
N	-0.44791700	0.47815200	0.00079100
C	-1.79142800	0.16415800	0.00387400
C	-2.34293800	-1.13789800	0.03580400
C	-2.63634400	1.29361900	-0.02552600
C	-3.71646800	-1.28499100	0.03146100
H	-1.72444600	-2.02684100	0.06999100
C	-4.01296100	1.12865600	-0.03149800
H	-2.18300600	2.27645400	-0.04583200
C	-4.55198800	-0.15743500	-0.00354400
H	-4.15019400	-2.27649900	0.05750300
H	-4.66296700	1.99378300	-0.05641000
H	-5.62701200	-0.29156600	-0.00645800
H	0.16156600	-1.41846200	-0.02463900

SCRF = (CPCM, solvent= dichloromethane)

C	3.68497300	1.32087600	0.02946300
C	2.31090500	1.13778800	0.02571800
C	1.81383600	-0.17067600	-0.00451200
C	2.66383300	-1.28250500	-0.03001100
C	4.03811900	-1.07799300	-0.02551800
C	4.54891600	0.21953200	0.00411800
H	4.08913100	2.32514600	0.05300700
H	1.63096500	1.97823400	0.04611000
H	2.25630100	-2.28704100	-0.05274700
H	4.70657800	-1.92924400	-0.04487600
H	5.62058000	0.37693400	0.00805900
N	0.42675900	-0.42647200	-0.00941300
N	-0.44858900	0.47936100	0.00092000
C	-1.79168600	0.16475700	0.00378500
C	-2.34281600	-1.13773600	0.03393600
C	-2.63748200	1.29376300	-0.02405900
C	-3.71610600	-1.28570300	0.02970900
H	-1.72402300	-2.02668800	0.06658900
C	-4.01398800	1.12798300	-0.02986300
H	-2.18470800	2.27690600	-0.04319500
C	-4.55227800	-0.15836300	-0.00350700
H	-4.14955900	-2.27739600	0.05440700
H	-4.66454500	1.99273600	-0.05346000
H	-5.62723000	-0.29311600	-0.00634000
H	0.16196900	-1.41702500	-0.02365800

Azobenzene **7**

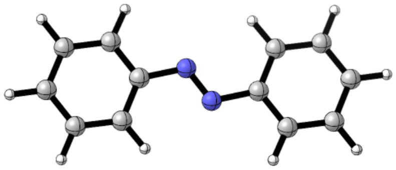

In gas phase

C	-3.67261000	-1.30057600	0.00000200
C	-2.29662200	-1.11850200	0.00000200
C	-1.77057200	0.18280400	0.00000000
C	-2.63313800	1.28418900	-0.00000200
C	-4.01225500	1.09347300	-0.00000200
C	-4.53426400	-0.19848300	0.00000000
H	-4.08212200	-2.30470200	0.00000300
H	-1.61765700	-1.96095500	0.00000300
H	-2.20121300	2.27804900	-0.00000300
H	-4.67701300	1.94966700	-0.00000300
H	-5.60772100	-0.35081600	0.00000000
N	-0.38568500	0.49319000	0.00000000
N	0.38568400	-0.49318800	-0.00000100
C	1.77057200	-0.18280200	0.00000000
C	2.29662300	1.11850300	0.00000300
C	2.63313700	-1.28418800	-0.00000300
C	3.67261100	1.30057500	0.00000300
H	1.61765900	1.96095700	0.00000500
C	4.01225400	-1.09347500	-0.00000300
H	2.20121100	-2.27804900	-0.00000500
C	4.53426500	0.19848100	0.00000000
H	4.08212500	2.30470100	0.00000500
H	4.67701100	-1.94966900	-0.00000500
H	5.60772200	0.35081300	0.00000000

SCRF = (CPCM, solvent=acetonitrile)

C	-3.67900600	-1.30077200	-0.00000100
C	-2.30207500	-1.12064000	-0.00000100
C	-1.77352400	0.18084700	0.00000000
C	-2.63552500	1.28427600	0.00000100
C	-4.01561100	1.09541900	0.00000100
C	-4.53952600	-0.19659000	0.00000000
H	-4.08963300	-2.30411100	-0.00000100
H	-1.63046800	-1.96889700	-0.00000100
H	-2.20734800	2.27989500	0.00000100
H	-4.67848100	1.95272700	0.00000100
H	-5.61288600	-0.34760100	0.00000000
N	-0.38855200	0.49157100	0.00000000
N	0.38855200	-0.49157100	0.00000000
C	1.77352400	-0.18084700	0.00000000
C	2.30207500	1.12064000	0.00000100
C	2.63552500	-1.28427600	-0.00000100
C	3.67900600	1.30077200	0.00000100
H	1.63046800	1.96889700	0.00000100
C	4.01561100	-1.09541900	-0.00000100
H	2.20734900	-2.27989500	-0.00000100
C	4.53952600	0.19659100	0.00000000
H	4.08963200	2.30411100	0.00000100
H	4.67848100	-1.95272700	-0.00000100
H	5.61288600	0.34760100	0.00000000

SCRF = (CPCM, solvent= dichloromethane)

C	-3.67833000	-1.30075600	-0.00000100
C	-2.30148800	-1.12041600	-0.00000100
C	-1.77320900	0.18104700	0.00000000
C	-2.63524700	1.28427100	0.00000100
C	-4.01524400	1.09522600	0.00000100
C	-4.53897400	-0.19678700	0.00000000
H	-4.08881000	-2.30418700	-0.00000100
H	-1.62913500	-1.96808200	-0.00000100
H	-2.20668000	2.27970200	0.00000100
H	-4.67828900	1.95243400	0.00000100
H	-5.61234800	-0.34793200	0.00000000
N	-0.38823300	0.49174100	0.00000000
N	0.38823300	-0.49174200	0.00000000
C	1.77320900	-0.18104800	0.00000000
C	2.30148700	1.12041600	0.00000100
C	2.63524800	-1.28427100	-0.00000100
C	3.67833000	1.30075600	0.00000100
H	1.62913500	1.96808200	0.00000100
C	4.01524400	-1.09522500	-0.00000100
H	2.20668100	-2.27970200	-0.00000100
C	4.53897400	0.19678700	0.00000000
H	4.08881000	2.30418700	0.00000100
H	4.67828900	-1.95243300	-0.00000100
H	5.61234800	0.34793300	0.00000000

Transition state 1 of hydrazine **28** & **1**

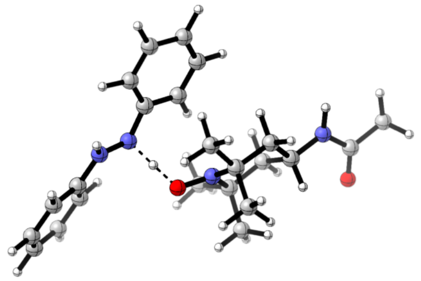

In gas phase

H	-1.35122400	0.06362900	-0.15434800
C	1.40113000	-1.11035800	-0.78586800
C	1.40332600	0.22436600	1.51928000
C	2.81606400	0.71139800	1.14142900
C	3.59043000	-0.27941600	0.27638500
C	2.80872600	-0.53408300	-1.01771000
H	2.73030700	1.66482600	0.60516400
H	3.35869900	0.91191400	2.06906400
H	3.72393100	-1.22494300	0.80548000
H	2.73040300	0.40991500	-1.56955600
H	3.36268100	-1.23424300	-1.64594400
N	0.71646600	-0.31039400	0.29298300
O	-0.55094800	-0.58246200	0.46640000
C	0.58205900	-0.98970500	-2.08041400
H	1.10313200	-1.52861000	-2.87378100
H	-0.40987300	-1.42963900	-1.97338800
H	0.48257200	0.04997500	-2.40008900
C	1.45178700	-2.60038300	-0.37059800
H	0.45422900	-2.95230000	-0.10279600
H	1.81336500	-3.18871100	-1.21662200
H	2.12555200	-2.78221200	0.46504800
C	0.58811800	1.40644900	2.06705300
H	-0.39570900	1.08445600	2.40890100
H	1.11822200	1.83265700	2.92113900
H	0.46752400	2.18785500	1.31483600
C	1.45476700	-0.88141000	2.60284000
H	1.81604200	-0.44531300	3.53679900
H	0.45646100	-1.28605600	2.77531600
H	2.12144700	-1.70014400	2.33641100
N	4.93593900	0.21085700	0.00962300
C	5.96397300	-0.65683000	-0.27136500
C	7.31651200	-0.02730100	-0.53013100
H	7.31069200	1.06324600	-0.48023100
H	8.02574000	-0.41343500	0.20474800
H	7.66648700	-0.33970600	-1.51583500
O	5.78461600	-1.86377300	-0.29902200
H	5.10404500	1.20451200	-0.02513100
C	-2.87809400	4.37625900	0.01387300
C	-3.14276000	3.01664700	0.09227500
C	-2.20757100	2.09409600	-0.41753100
C	-1.02773000	2.56675400	-1.02784400
C	-0.77590500	3.92943000	-1.09254700
C	-1.69474200	4.83919300	-0.56824700
H	-3.60784200	5.08297100	0.39007400
H	-4.09382100	2.69154300	0.49561800
H	-0.33470800	1.85770100	-1.46070200
H	0.12952000	4.28569000	-1.56886100
H	-1.50501600	5.90352500	-0.63441300
N	-2.39750700	0.71155400	-0.41252900
N	-3.39364400	0.23068800	0.31352400
H	-3.72179600	0.76534100	1.11471600
C	-3.95503000	-1.03060500	0.09771200
C	-4.75550000	-1.57884100	1.11237800
C	-3.75307200	-1.71944400	-1.10703200
C	-5.34162800	-2.82168300	0.92149500
H	-4.90698400	-1.03818200	2.04091500
C	-4.33979600	-2.96545200	-1.27610300
H	-3.16573400	-1.26799200	-1.89548000
C	-5.13182300	-3.52199100	-0.26815000
H	-5.95970000	-3.24748600	1.70229900
H	-4.19442100	-3.50076300	-2.20663200
H	-5.59180300	-4.49160100	-0.41403200

Imaginary frequency: -1130.8143 cm^{-1}

SCRF = (CPCM, solvent=acetonitrile)

H	-1.41781800	0.03910500	-0.23431500
C	1.33299200	-1.20576900	-0.96855400
C	1.35188900	-0.03719500	1.41739400
C	2.76082900	0.47657600	1.06625500
C	3.53678200	-0.44314800	0.12163100
C	2.74203700	-0.62020500	-1.17622700
H	2.67033900	1.46390400	0.60003500
H	3.31262000	0.60955900	1.99985900
H	3.71554500	-1.41077600	0.59056300
H	2.65484600	0.35536900	-1.66646600
H	3.28239500	-1.27813500	-1.86099400
N	0.65687300	-0.46965700	0.15738600
O	-0.61626400	-0.72539800	0.30198500
C	0.51347200	-1.01315100	-2.25377200
H	1.03293300	-1.51375100	-3.07303000
H	-0.48013900	-1.45249600	-2.16713900
H	0.41911900	0.04135800	-2.51821000
C	1.38298700	-2.71822300	-0.64372700
H	0.38539800	-3.08147200	-0.39162700
H	1.73332200	-3.25502700	-1.52814600
H	2.05805200	-2.95213400	0.17825200
C	0.54794500	1.10224600	2.06299700
H	-0.44074900	0.76483600	2.37412500
H	1.08443500	1.44623400	2.94952600
H	0.44132600	1.94498600	1.37873500
C	1.40635800	-1.22149700	2.41407700
H	1.77123200	-0.85438100	3.37573200

H	0.40743300	-1.63574100	2.55928000
H	2.07122800	-2.01923100	2.08601000
N	4.86250700	0.10589100	-0.14775800
C	6.01548200	-0.60202000	-0.03597500
C	7.28651100	0.14955300	-0.37346500
H	7.11375500	1.18580200	-0.66602700
H	7.94658100	0.13005900	0.49634700
H	7.79595800	-0.37047700	-1.18757300
O	6.04285200	-1.78211000	0.32058200
H	4.92092200	1.06264900	-0.46630800
C	-2.46491900	4.46149700	0.17047600
C	-2.86773800	3.13529600	0.21709000
C	-2.04908400	2.14117500	-0.35564400
C	-0.84535900	2.50900000	-0.99125800
C	-0.45345500	3.84009500	-1.01976800
C	-1.25722200	4.82034900	-0.43745200
H	-3.10340400	5.22485700	0.59813600
H	-3.82740300	2.89302500	0.65472100
H	-0.23795700	1.75172000	-1.46647700
H	0.47379500	4.11333400	-1.50809000
H	-0.95790300	5.86073200	-0.47290600
N	-2.38364400	0.78773400	-0.38345400
N	-3.38909700	0.38286600	0.37238800
H	-3.63285600	0.92049900	1.20141200
C	-4.06383100	-0.81717700	0.14753300
C	-4.89110900	-1.31021700	1.16963900
C	-3.94269300	-1.50320600	-1.07025800
C	-5.58223200	-2.49774900	0.97234400
H	-4.98129200	-0.76885900	2.10460700
C	-4.63412500	-2.69430000	-1.24465200
H	-3.33087500	-1.09525400	-1.86346200
C	-5.45248000	-3.19785600	-0.22945500
H	-6.21994700	-2.88060700	1.75972600
H	-4.54570200	-3.22696600	-2.18385100
H	-5.99257100	-4.12493600	-0.37827000

Imaginary frequency: - -1184.0159 cm^{-1}

SCRF = (CPCM, solvent= dichloromethane)

H	-1.41355800	0.04101500	-0.23106100
C	1.33725000	-1.19629100	-0.96273800
C	1.35708200	-0.01713900	1.41873700
C	2.76593200	0.49497500	1.06460200
C	3.54114000	-0.42948100	0.12417800
C	2.74605600	-0.61116900	-1.17292600
H	2.67552800	1.48062200	0.59434600
H	3.31814100	0.63191100	1.99739200
H	3.71725900	-1.39587900	0.59684500
H	2.65879400	0.36258100	-1.66719500
H	3.28661000	-1.27164300	-1.85503600
N	0.66146500	-0.45640800	0.16119500
O	-0.61081800	-0.71460300	0.30914200
C	0.51631200	-1.00784700	-2.24766000
H	1.03414900	-1.51165900	-3.06595100
H	-0.47749300	-1.44629000	-2.15827900
H	0.42231800	0.04587900	-2.51578200
C	1.38778600	-2.70748400	-0.63231900
H	0.39057800	-3.07018900	-0.37800900
H	1.73777300	-3.24759100	-1.51483500
H	2.06385000	-2.93810500	0.18971000
C	0.55241700	1.12498900	2.05867100
H	-0.43574600	0.78794900	2.37203300
H	1.08861600	1.47422800	2.94330400
H	0.44434400	1.96414300	1.37013300
C	1.41253400	-1.19668200	2.42099000
H	1.77757400	-0.82497800	3.38081900
H	0.41403000	-1.61107200	2.56850800
H	2.07796400	-1.99534200	2.09649600
N	4.86874400	0.11343000	-0.14524300
C	6.01629400	-0.60856100	-0.04261600
C	7.29413500	0.13259400	-0.37759600
H	7.13164900	1.17203500	-0.66526000
H	7.95450600	0.10232800	0.49163700
H	7.79827000	-0.38889400	-1.19397600
O	6.03001400	-1.78997200	0.30433400
H	4.93450800	1.07112700	-0.45887900
C	-2.49504800	4.45573300	0.17099300
C	-2.88747400	3.12636000	0.21818200
C	-2.06344200	2.13860000	-0.35765400
C	-0.86468000	2.51604500	-0.99692700
C	-0.48330800	3.85014700	-1.02653600
C	-1.29242800	4.82408000	-0.44101100
H	-3.13800500	5.21411500	0.60082100
H	-3.84427400	2.87694700	0.65824600
H	-0.25371500	1.76316300	-1.47479900
H	0.43956500	4.13118400	-1.51878400
H	-1.00148000	5.86678200	-0.47755400
N	-2.38732700	0.78235200	-0.38553300
N	-3.38994700	0.36997700	0.37040000
H	-3.63773500	0.90606300	1.19914500
C	-4.05646600	-0.83512200	0.14633900
C	-4.87664600	-1.33553300	1.17054000
C	-3.93434700	-1.51862000	-1.07271600
C	-5.56011000	-2.52751300	0.97396900
H	-4.96735800	-0.79673700	2.10706100
C	-4.61803000	-2.71417600	-1.24647600
H	-3.32817000	-1.10504400	-1.86736900
C	-5.42946500	-3.22476800	-0.22930100
H	-6.19244000	-2.91614300	1.76282900
H	-4.52941600	-3.24493300	-2.18671400
H	-5.96370300	-4.15525200	-0.37773200

Imaginary frequency: -1178.6887 cm^{-1}

Transition state 2 of protonated diazene **29** & **30**

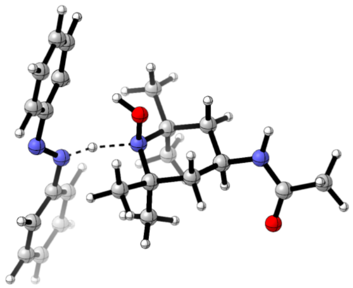

In gas phase

C	2.27977600	-0.24888500	-1.48031200
C	0.85136400	0.28849100	-1.23623200
C	1.26099700	-0.01694300	1.32816000
C	2.66629800	-0.55672800	0.99616200
C	3.23692200	0.01025200	-0.31383300
H	2.22184600	-1.32423100	-1.67514100
H	2.66935400	0.22325500	-2.38650300
H	3.33707100	-0.29770400	1.81948600
H	2.63047500	-1.64712600	0.93111800
H	3.40111000	1.08216100	-0.19610300
C	0.67123000	-0.78983400	2.51924800
H	1.33237400	-0.66627300	3.37879200
H	-0.30824400	-0.39529600	2.80961000
H	0.58946400	-1.85768200	2.32125100
C	1.32868000	1.47003400	1.73081600
H	0.36780200	1.97229900	1.63372800
H	1.62489500	1.52453700	2.77990000
H	2.06517900	2.03991200	1.17150300
C	0.81747400	1.82708000	-1.28724100
H	0.93890000	2.12620500	-2.33028500
H	-0.13729700	2.22942500	-0.94603700
H	1.61257100	2.30791400	-0.72618500
C	-0.07926200	-0.22499300	-2.34930500
H	-1.08313100	0.19924400	-2.26527500
H	0.32238800	0.09892300	-3.31155200
H	-0.14684400	-1.31051500	-2.35969600
N	0.32753900	-0.24463800	0.11606000
N	4.55842900	-0.52944500	-0.60202100
C	5.70047600	0.10595200	-0.17786200
C	7.00721500	-0.57849100	-0.52113100
H	7.55097300	-0.78024500	0.40386900
H	6.88274300	-1.51123100	-1.07460500
H	7.61495800	0.10887600	-1.11278600
O	5.65673000	1.16351200	0.43085600
H	4.63435100	-1.43420700	-1.04146500
C	-2.51231400	3.99702200	1.10755800
C	-2.18572400	2.64510400	1.13723200
C	-2.63538000	1.81270500	0.10938200
C	-3.40779800	2.31370800	-0.94421400
C	-3.70839100	3.66840500	-0.97142300
C	-3.26376500	4.51041900	0.05151000
H	-2.18614800	4.64561500	1.91123100
H	-1.63450300	2.23343900	1.97228200
H	-3.74967500	1.64902100	-1.72661300
H	-4.29010300	4.07126200	-1.79152400
H	-3.50610300	5.56599200	0.02467700
N	-2.31848100	0.41894300	0.18877300
N	-3.18602600	-0.38093400	-0.24532800
C	-3.02207200	-1.75407000	-0.07026900
C	-3.57637200	-2.58225800	-1.06039600
C	-2.47737600	-2.31301700	1.10430100
C	-3.49669000	-3.96109900	-0.92120500
H	-4.04031600	-2.12964500	-1.92789100
C	-2.44798200	-3.69461700	1.24847800
H	-2.16975900	-1.66993600	1.92001700
C	-2.93492500	-4.51850100	0.22938000
H	-3.89605100	-4.60373600	-1.69611900
H	-2.06832900	-4.13144600	2.16434500
H	-2.90658200	-5.59486600	0.34901700
H	-1.16983800	0.13077000	0.34032400
O	0.33117200	-1.68316100	-0.08164700
H	-0.51128000	-2.00342400	0.26774200

Imaginary frequency: -617.9569 cm^{-1}

SCRF = (CPCM, solvent=acetonitrile)

C	2.32763900	-0.29650000	-1.31753500
C	0.88775500	0.23634600	-1.15186400
C	1.18459400	0.00149800	1.43512200
C	2.60868600	-0.53529500	1.18081900
C	3.23833300	-0.00965300	-0.11676700
H	2.28750000	-1.37614500	-1.48665000
H	2.75412300	0.15511500	-2.21708300
H	3.23424300	-0.25353600	2.03211900
H	2.57841400	-1.62743900	1.14778600
H	3.42147800	1.06135000	-0.03478100
C	0.55951100	-0.75020400	2.62180400
H	1.19281900	-0.59826300	3.49792600
H	-0.43138200	-0.36121800	2.86956200
H	0.49405300	-1.82236100	2.44380700
C	1.22002500	1.49876000	1.79873300
H	0.25604500	1.98431200	1.66147500
H	1.48062100	1.58204900	2.85527200
H	1.96350300	2.06461500	1.24406000
C	0.84577900	1.77291400	-1.24448600
H	0.99630000	2.04268800	-2.29155800
H	-0.12048800	2.17810000	-0.94262900
H	1.62087300	2.27367300	-0.67259700
C	0.01435800	-0.31513800	-2.29232200
H	-0.99578100	0.09885100	-2.26670600
H	0.46020600	-0.01060500	-3.24131600
H	-0.04411800	-1.40105600	-2.27626300

N	0.30949500	-0.26789200	0.18956200
N	4.55850200	-0.59795400	-0.32503500
C	5.71713600	0.11211300	-0.31486200
C	6.98290500	-0.68822500	-0.54381400
H	7.64332300	-0.55296000	0.31535800
H	6.80309400	-1.75395200	-0.69045000
H	7.49615100	-0.28912700	-1.42125100
O	5.75214200	1.33017600	-0.13363900
H	4.61353800	-1.59566000	-0.47153800
C	-2.56908300	3.99757400	1.10433200
C	-2.26223400	2.64054700	1.15317000
C	-2.61146700	1.82795300	0.07317000
C	-3.26311000	2.34902800	-1.04894300
C	-3.54547600	3.70851500	-1.09112300
C	-3.20051000	4.53314200	-0.01748600
H	-2.31939800	4.63198200	1.94576700
H	-1.80896700	2.21388500	2.03777200
H	-3.52176600	1.69910700	-1.87471500
H	-4.03252200	4.12633400	-1.96370300
H	-3.42561800	5.59214700	-0.05635200
N	-2.31298100	0.42616200	0.15429100
N	-3.19372100	-0.34997300	-0.28665500
C	-3.04488800	-1.73018700	-0.13068500
C	-3.56019100	-2.53797400	-1.15654800
C	-2.54309100	-2.30595100	1.05319500
C	-3.48369600	-3.91917000	-1.03762100
H	-3.98457500	-2.07073800	-2.03617800
C	-2.51716500	-3.69078100	1.17393300
H	-2.26679900	-1.67650000	1.89016600
C	-2.96466700	-4.49644900	0.12357700
H	-3.84900400	-4.54787600	-1.84005400
H	-2.16432900	-4.14116100	2.09347900
H	-2.93603500	-5.57459500	0.22364100
H	-1.14961900	0.11179800	0.33331000
O	0.32775000	-1.70887800	0.03594600
H	-0.54095500	-2.02277400	0.32593700

Imaginary frequency: -911.3299 cm^{-1}

SCRF = (CPCM, solvent= dichloromethane)

C	2.32763900	-0.29649400	-1.31753400
C	0.88775500	0.23635100	-1.15186400
C	1.18459300	0.00150300	1.43512300
C	2.60868600	-0.53528900	1.18082000
C	3.23833200	-0.00964600	-0.11676600
H	2.28750200	-1.37613900	-1.48665000
H	2.75412300	0.15512200	-2.21708200
H	3.23424300	-0.25352900	2.03212000
H	2.57841500	-1.62743200	1.14778600
H	3.42147600	1.06135700	-0.03478000
C	0.55951100	-0.75019800	2.62180500
H	1.19282000	-0.59825700	3.49792700
H	-0.43138200	-0.36121100	2.86956300
H	0.49405300	-1.82235500	2.44380900
C	1.22002400	1.49876500	1.79873300
H	0.25603900	1.98431100	1.66149100
H	1.48063600	1.58205400	2.85526900
H	1.96349000	2.06462500	1.24405000
C	0.84577700	1.77291800	-1.24448700
H	0.99628600	2.04269100	-2.29156100
H	-0.12048700	2.17810400	-0.94261900
H	1.62087700	2.27367700	-0.67260700
C	0.01435800	-0.31513600	-2.29232100
H	-0.99578100	0.09885500	-2.26670700
H	0.46020800	-0.01060600	-3.24131600
H	-0.04411900	-1.40105400	-2.27625900
N	0.30949500	-0.26788800	0.18956200
N	4.55850200	-0.59794700	-0.32503400
C	5.71713600	0.11212100	-0.31486400
C	6.98290600	-0.68821700	-0.54381000
H	7.64331500	-0.55296200	0.31537000
H	6.80309400	-1.75394300	-0.69045700
H	7.49616100	-0.28911300	-1.42123800
O	5.75214000	1.33018500	-0.13364700
H	4.61353900	-1.59565300	-0.47153100
C	-2.56909800	3.99756800	1.10433200
C	-2.26224300	2.64054200	1.15316800
C	-2.61147300	1.82794700	0.07316900
C	-3.26312000	2.34902000	-1.04894300
C	-3.54549300	3.70850500	-1.09112300
C	-3.20052900	4.53313400	-0.01748600
H	-2.31941500	4.63197700	1.94576600
H	-1.80897300	2.21388300	2.03777000
H	-3.52177500	1.69909700	-1.87471500
H	-4.03254200	4.12632300	-1.96370200
H	-3.42564200	5.59213800	-0.05635100
N	-2.31298100	0.42615800	0.15429000
N	-3.19371800	-0.34998100	-0.28665600
C	-3.04488000	-1.73019400	-0.13068600
C	-3.56017700	-2.53798400	-1.15655000
C	-2.54308300	-2.30595600	1.05319600
C	-3.48367700	-3.91918000	-1.03762200
H	-3.98456100	-2.07075000	-2.03618100
C	-2.51715200	-3.69078600	1.17393500
H	-2.26679600	-1.67650200	1.89016700
C	-2.96464800	-4.49645500	0.12357800
H	-3.84898000	-4.54788800	-1.84005500
H	-2.16431700	-4.14116300	2.09348200
H	-2.93601200	-5.57460200	0.22364300
H	-1.14961700	0.11179800	0.33330800
O	0.32775200	-1.70887400	0.03594700
H	-0.54095300	-2.02277100	0.32593700

Imaginary frequency: -910.8210 cm^{-1}

Hydrazide **6**

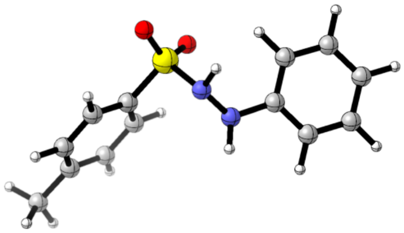

In gas phase

N	-0.61117400	0.64950800	-1.07367000
H	-1.22512900	1.34467500	-1.50125000
N	-1.22171200	-0.55012700	-0.72048800
H	-0.94948400	-1.28068600	-1.36415300
C	-2.58995800	-0.58744200	-0.36499500
C	-3.22365300	0.47886400	0.28064300
C	-3.31781200	-1.75194100	-0.64665600
C	-4.56998300	0.37506900	0.62593500
H	-2.66486700	1.36447500	0.55148600
C	-4.65700600	-1.84656200	-0.28627300
H	-2.83099200	-2.58221400	-1.14946500
C	-5.29540700	-0.78097800	0.34855200
H	-5.04816200	1.20759500	1.12974800
H	-5.20533400	-2.75451900	-0.51135200
H	-6.34068700	-0.85324500	0.62409600
S	0.33834200	1.43260800	0.13656200
O	0.58045600	2.74833700	-0.45396000
O	-0.25893900	1.29600300	1.46258400
C	1.84639900	0.46760200	0.11506400
C	2.78947300	0.69792800	-0.88545300
C	2.05795800	-0.49106900	1.10074200
C	3.95939600	-0.05151000	-0.89193400
H	2.61132600	1.45901500	-1.63489900
C	3.23643400	-1.23326500	1.07610400
H	1.31611100	-0.64155700	1.87444200
C	4.20227300	-1.02665300	0.08584700
H	4.69881900	0.12604400	-1.66578200
H	3.40666200	-1.98193000	1.84236000
C	5.49145100	-1.80950500	0.08429400
H	6.29741100	-1.22780300	0.54442300
H	5.39437600	-2.74009100	0.64673800
H	5.80715400	-2.05510600	-0.93247900

Transition state 1 of Hydrazide **6** & **1**

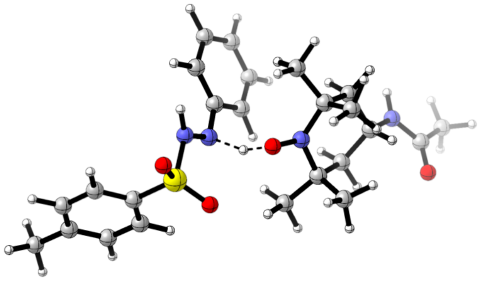

In gas phase

H	-0.14069600	0.23848000	0.37966100
C	2.28296000	-1.18838100	-0.38170000
C	2.87351300	0.80371200	1.30070700
C	4.18205600	0.97181300	0.50433200
C	4.69276200	-0.32885000	-0.10953900
C	3.62738100	-0.88128000	-1.06315400
H	4.01988900	1.70541300	-0.29534300
H	4.92949200	1.39389500	1.18113900
H	4.89434000	-1.06677100	0.66939800
H	3.47071500	-0.15492200	-1.86917900
H	3.98982400	-1.80344400	-1.52110900
N	1.90911400	-0.03053700	0.50765000
O	0.71482200	-0.09326700	1.03654400
C	1.19112900	-1.35396800	-1.45201500
H	1.49469400	-2.15230100	-2.13195400
H	0.23218900	-1.63249900	-1.01500100
H	1.07663800	-0.44261800	-2.04488300
C	2.34681000	-2.47864600	0.47096800
H	1.40964000	-2.62260700	1.00994200
H	2.49728500	-3.32917800	-0.19717400
H	3.17124600	-2.46936600	1.18275700
C	2.24101100	2.18404400	1.54375900
H	1.34865100	2.10780700	2.16478900
H	2.96378100	2.81454000	2.06545900
H	1.98075200	2.66962900	0.60095100
C	3.12632600	0.12138800	2.66831900
H	3.72094900	0.79211400	3.29229700
H	2.18020700	-0.07334800	3.17482400
H	3.66938000	-0.81779200	2.57093700
N	5.96618400	-0.11414700	-0.78321600
C	6.86353400	-1.14208400	-0.94878700
C	8.15076000	-0.79428800	-1.66640100
H	8.20596600	0.24665900	-1.99079400
H	8.98844400	-1.00367400	-0.99812400
H	8.25595500	-1.44715800	-2.53494600
O	6.62747300	-2.26320000	-0.52875600
H	6.16641800	0.78683200	-1.18940600
C	-1.85259500	4.47680200	0.08738500
C	-1.99373800	3.16160200	0.49881400
C	-1.25594000	2.14725500	-0.14672800
C	-0.38578900	2.48514600	-1.20701700
C	-0.25041100	3.80706300	-1.60167900
C	-0.98073000	4.80665200	-0.95684000
H	-2.42814000	5.25387400	0.57550800
H	-2.68998400	2.93852700	1.29797500
H	0.14565700	1.69391100	-1.72030800
H	0.40972700	4.05971300	-2.42248100
H	-0.88474100	5.83847000	-1.27252700
N	-1.33352800	0.79260200	0.15405300
N	-2.10284600	0.38146600	1.13717400
H	-2.52254800	1.02122500	1.80961000
S	-2.74000900	-1.28627700	1.17428200
O	-1.73519200	-2.06297000	0.47576800
O	-3.06115500	-1.43373800	2.58005400
C	-4.22887300	-1.19896900	0.21409800
C	-4.16628300	-1.39590000	-1.16707000
C	-5.43863600	-0.94639900	0.86558500
C	-5.34327000	-1.33122600	-1.90090900
H	-3.22251600	-1.61688000	-1.64876400
C	-6.60136500	-0.88744000	0.10809100
H	-5.46803500	-0.82482600	1.94111700
C	-6.57601500	-1.07738600	-1.28093300
H	-5.30739100	-1.49153300	-2.97260100
H	-7.54656800	-0.70216200	0.60572300
C	-7.84860300	-1.05086500	-2.08401600
H	-8.28060500	-2.05585000	-2.14113200
H	-8.59628900	-0.39935500	-1.62845300
H	-7.67019800	-0.71340300	-3.10653500

Imaginary frequency: -924.7425 cm^{-1}

Hydrazide **S13**

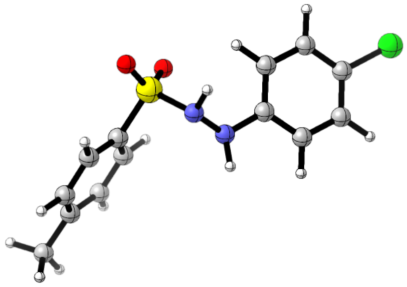

In gas phase

N	0.20232200	0.80277900	-1.13267100
H	-0.35594500	1.54789000	-1.55223400
N	-0.47862900	-0.38146500	-0.86858400
H	-0.21746500	-1.09083400	-1.53985300
C	-1.85641600	-0.37146800	-0.55630400
C	-2.45495300	0.67289300	0.15532300
C	-2.63760300	-1.46784400	-0.94504300
C	-3.81341100	0.62424400	0.45869000
H	-1.86186100	1.50361500	0.51261300
C	-3.99015700	-1.52341000	-0.63147400
H	-2.18608700	-2.28468800	-1.49924700
C	-4.57389000	-0.47068600	0.06659400
H	-4.27181700	1.43311300	1.01316700
H	-4.58753700	-2.37313000	-0.93626800
S	1.13855200	1.47612700	0.15235900
O	1.47655800	2.80054200	-0.36572300
O	0.46973300	1.31482100	1.44128800
C	2.58966500	0.42878400	0.15329500
C	3.58733500	0.64508100	-0.79623900
C	2.70398200	-0.57874800	1.10609900
C	4.71364300	-0.16838800	-0.78438700
H	3.48497800	1.44407900	-1.51996700
C	3.84016800	-1.38415600	1.10030600
H	1.92161300	-0.71764100	1.84105300
C	4.85910300	-1.19334000	0.16124100
H	5.49537300	-0.00244200	-1.51811900
H	3.93514800	-2.17066200	1.84121700
C	6.10255800	-2.04645100	0.18280000
H	6.91100100	-1.53689500	0.71801400
H	5.92464400	-2.99921000	0.68514700
H	6.46138900	-2.25355100	-0.82810600
Cl	-6.28979900	-0.52875600	0.45433600

Transition state of Hydrazide **S13** & **1**

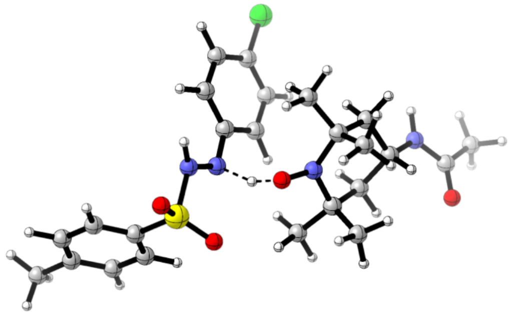

In gas phase

H	-0.08416100	-0.20822300	0.45427600
C	2.38103100	-1.45088900	-0.49196100
C	2.92163700	0.32829000	1.42898000
C	4.21759600	0.63948300	0.65532600
C	4.76439400	-0.55476000	-0.12184300
C	3.70932000	-1.01559800	-1.13392900
H	4.02567500	1.46340600	-0.04344000
H	4.95656800	0.99612100	1.37756900
H	4.99518600	-1.37925200	0.55555700
H	3.52343200	-0.19812800	-1.84035400
H	4.09743500	-1.85961700	-1.70698300
N	1.97752900	-0.42900100	0.53988400
O	0.78980800	-0.59601500	1.06009000
C	1.28587200	-1.51441700	-1.56966800
H	1.60856800	-2.20965500	-2.34701300
H	0.33989400	-1.87687600	-1.16736000
H	1.13800000	-0.53941700	-2.04180400
C	2.49279300	-2.83652000	0.18898000
H	1.56431900	-3.07949600	0.70704500
H	2.66731400	-3.58921200	-0.58266400
H	3.32091700	-2.89014400	0.89440700
C	2.24737200	1.64489300	1.84889800
H	1.36354200	1.46080600	2.45944800
H	2.95401500	2.22776500	2.44296200
H	1.96403700	2.23769400	0.97664200
C	3.20699600	-0.51395800	2.69750900
H	3.78303500	0.09187400	3.40033100
H	2.27162900	-0.80419500	3.17759600
H	3.78097700	-1.41371000	2.47972800
N	6.02448300	-0.21402500	-0.76799000
C	6.94766500	-1.18432400	-1.07735200
C	8.21844600	-0.70640400	-1.74767100
H	8.24265200	0.37011300	-1.92754600
H	9.06747500	-0.98125300	-1.11857300
H	8.33335100	-1.23189600	-2.69757800
O	6.74503100	-2.35828000	-0.81308400
H	6.19708500	0.73952900	-1.04687200
C	-1.94635100	3.97142400	0.69778400
C	-2.03633600	2.61222400	0.93343100
C	-1.26408900	1.70874700	0.17165200
C	-0.40782500	2.21296000	-0.83368000
C	-0.31314600	3.57317900	-1.06264300
C	-1.08057800	4.45230400	-0.29343500
H	-2.54491200	4.66730400	1.27121500
H	-2.72650300	2.27066500	1.69483200
H	0.15416900	1.51635600	-1.44251900
H	0.33399400	3.95870500	-1.83964400
N	-1.29137500	0.33009400	0.30264700
N	-2.04283700	-0.22944300	1.22862100
H	-2.47315300	0.30304600	1.98247400
S	-2.63600900	-1.90091700	1.04513700
O	-1.61280700	-2.55334000	0.25231300
O	-2.95104000	-2.24001800	2.41882500
C	-4.12874100	-1.72978300	0.10177100
C	-4.06432300	-1.74976300	-1.29307800
C	-5.34305600	-1.59126100	0.77850800
C	-5.24411500	-1.62220100	-2.01422800
H	-3.11646300	-1.88580000	-1.79768300
C	-6.50848100	-1.46583000	0.03331900
H	-5.37333000	-1.60751600	1.86075600
C	-6.48141800	-1.47876900	-1.36856800
H	-5.20658800	-1.64584200	-3.09753700
H	-7.45694200	-1.36826900	0.54935000
C	-7.75599600	-1.38311900	-2.16321200
H	-8.16349500	-2.38334100	-2.34590800
H	-8.51840400	-0.81219100	-1.63047000
H	-7.58808000	-0.91623700	-3.13535900
Cl	-0.97703700	6.15846900	-0.57962900

Imaginary frequency: -952.4918 cm^{-1}

Hydrazide **S15**

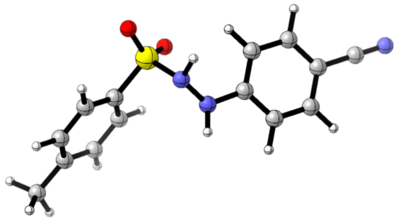

In gas phase

N	0.06353300	0.80339500	-1.13271500
H	-0.46137900	1.56746700	-1.55957700
N	-0.64531500	-0.36863500	-0.92638000
H	-0.35450200	-1.09620600	-1.56410800
C	-2.00376600	-0.37587800	-0.58643700
C	-2.62854200	0.70861000	0.04317300
C	-2.75355100	-1.52933700	-0.87263300
C	-3.97645300	0.64078600	0.36579000
H	-2.06012500	1.58610800	0.31781700
C	-4.09551500	-1.59613700	-0.54358000
H	-2.27677000	-2.37461900	-1.35804900
C	-4.72768100	-0.50684100	0.07770700
H	-4.45157100	1.48117800	0.85657800
H	-4.66523500	-2.48868900	-0.77076400
S	0.97214500	1.43252200	0.19995600
O	1.28596100	2.78615100	-0.25184100
O	0.28172800	1.18692700	1.46260700
C	2.44657400	0.41985900	0.17288600
C	3.44945600	0.69896000	-0.75486900
C	2.57344100	-0.62428800	1.08401300
C	4.59329900	-0.08927900	-0.76425300
H	3.33777500	1.52600000	-1.44491500
C	3.72773400	-1.40319700	1.05739400
H	1.78800800	-0.81056900	1.80520300
C	4.75190000	-1.15016700	0.13899500
H	5.37903200	0.12526100	-1.48083100
H	3.83315600	-2.21726100	1.76635200
C	6.01406900	-1.97528500	0.13862300
H	6.80892300	-1.46420200	0.69231100
H	5.85641600	-2.94697000	0.61034800
H	6.38060600	-2.14233000	-0.87691800
C	-6.11499400	-0.57049600	0.41011100
N	-7.23907500	-0.62533200	0.67618000

Transition state of Hydrazide **S15** & **1**

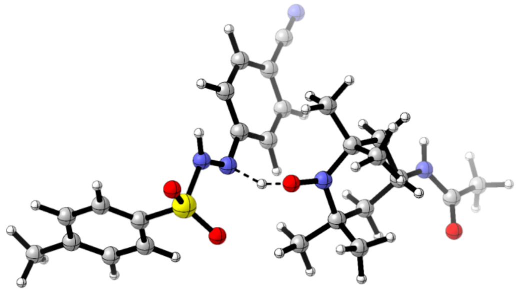

In gas phase

H	-0.07640200	-0.10796200	0.39388300
C	2.35386400	-1.37405600	-0.51205900
C	2.90914000	0.39367000	1.41698000
C	4.22689700	0.66718300	0.66599900
C	4.75411800	-0.54383300	-0.09844200
C	3.70539500	-0.97633800	-1.12948500
H	4.07061400	1.49457000	-0.03735800
H	4.96165200	1.00498000	1.40138000
H	4.94750100	-1.37330100	0.58470600
H	3.55559500	-0.15525100	-1.84012700
H	4.08015600	-1.83196300	-1.69399300
N	1.96271700	-0.34222600	0.51376000
O	0.76099000	-0.47611700	1.01887800
C	1.27727600	-1.40509200	-1.61013900
H	1.59635000	-2.10717700	-2.38271800
H	0.31407000	-1.74397000	-1.22863600
H	1.16565400	-0.42579100	-2.08328300
C	2.41380700	-2.76276200	0.17020200
H	1.46857300	-2.98190900	0.66809200
H	2.58482900	-3.51848100	-0.59922300
H	3.22544000	-2.83838200	0.89250500
C	2.26237200	1.72947300	1.82019700
H	1.36476700	1.57235400	2.41803200
H	2.97488900	2.29646300	2.42239200
H	2.00924400	2.32536900	0.94076000
C	3.14953400	-0.45186900	2.69328300
H	3.73068800	0.14109300	3.40272500
H	2.19914200	-0.71437200	3.15954200
H	3.70208900	-1.36766000	2.48734100
N	6.03509300	-0.24138700	-0.72142000
C	6.92920800	-1.24198900	-1.02086900
C	8.22867800	-0.80578100	-1.66330000
H	8.29325100	0.26983800	-1.83822500
H	9.05482500	-1.11156000	-1.01816400
H	8.34503500	-1.33178900	-2.61271000
O	6.67838700	-2.40936300	-0.76890400
H	6.24664700	0.70792300	-0.98772300
C	-1.93706900	4.10428600	0.61963400
C	-2.05076600	2.74648200	0.84924300
C	-1.26754000	1.83878100	0.10454500
C	-0.38192000	2.33071900	-0.88002900
C	-0.26638000	3.68976000	-1.10194200
C	-1.04004700	4.58985300	-0.35051300
H	-2.54318900	4.80315400	1.18202000
H	-2.76701300	2.40793100	1.58731300
H	0.18480300	1.62690700	-1.47572900
H	0.40569800	4.06589200	-1.86268100
N	-1.31499200	0.45819000	0.23168900
N	-2.06967200	-0.08833200	1.15414500
H	-2.47667100	0.44381300	1.92240800
S	-2.64134400	-1.78104500	1.00607100
O	-1.63123700	-2.41753300	0.18472000
O	-2.90029500	-2.11050900	2.39308500
C	-4.16522500	-1.63348900	0.11464000
C	-4.14770500	-1.66148800	-1.28199300
C	-5.35747100	-1.50529700	0.83250800
C	-5.35273400	-1.55284800	-1.96258200
H	-3.21612900	-1.78873300	-1.81828600
C	-6.54867000	-1.39855400	0.12676000
H	-5.35085800	-1.51491700	1.91523300
C	-6.56922500	-1.42020500	-1.27542500
H	-5.35211500	-1.58274300	-3.04633000
H	-7.48007300	-1.30886600	0.67421600
C	-7.87077500	-1.34373400	-2.02646400
H	-8.27010300	-2.35015900	-2.19276200
H	-8.62252600	-0.78201600	-1.46948500
H	-7.74215000	-0.87770600	-3.00494900
C	-0.92875400	5.99403900	-0.58012000
N	-0.83337400	7.13058900	-0.76435900

Imaginary frequency: -790.6946 cm^{-1}

Hydrazide **S21**

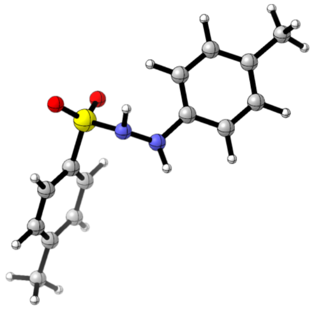

In gas phase

N	-0.18146100	0.72719300	-1.10406100
H	-0.77405800	1.44603800	-1.52300400
N	-0.82789500	-0.46428700	-0.78043200
H	-0.56962700	-1.18334900	-1.44288400
C	-2.20907900	-0.46403100	-0.46393900
C	-2.81861800	0.58463600	0.22893600
C	-2.97960700	-1.57635400	-0.82354100
C	-4.17601600	0.51537800	0.53638900
H	-2.23373400	1.42895800	0.56864500
C	-4.32917600	-1.63371200	-0.49600400
H	-2.51916300	-2.40113900	-1.35952100
C	-4.95993300	-0.58597100	0.18412100
H	-4.62726500	1.33874700	1.08089500
H	-4.90321000	-2.50906100	-0.78331100
S	0.76776900	1.46191800	0.13502600
O	1.06707000	2.77635700	-0.43195300
O	0.14029900	1.33061500	1.44799900
C	2.24216500	0.44577400	0.13144700
C	3.21561500	0.65922000	-0.84331800
C	2.39783800	-0.53526500	1.10562000
C	4.35972500	-0.12930800	-0.83492400
H	3.08067500	1.43743500	-1.58415000
C	3.55098600	-1.31627400	1.09614500
H	1.63296600	-0.67291400	1.85897900
C	4.54656200	-1.12705000	0.13211200
H	5.12267900	0.03493600	-1.58861400
H	3.67774000	-2.08236700	1.85358200
C	5.80863100	-1.95277200	0.14884400
H	6.61694800	-1.41297700	0.65380200
H	5.66171200	-2.89600800	0.67855000
H	6.15087200	-2.17824100	-0.86393700
C	-6.43550300	-0.63435800	0.50028100
H	-7.03420800	-0.26052100	-0.33795500
H	-6.76638200	-1.65531100	0.70693400
H	-6.67524900	-0.02051000	1.37164600

Transition state of Hydrazide **S21** & **1**

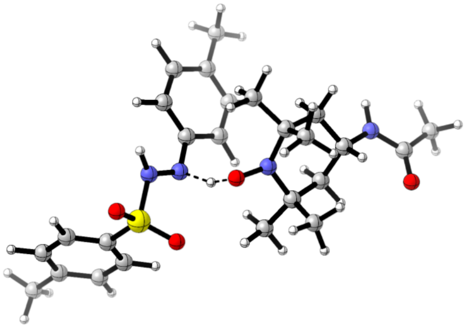

In gas phase

H	-0.12725700	-0.01961100	0.46409400
C	2.33111500	-1.32832100	-0.45159000
C	2.89537700	0.50644900	1.40718300
C	4.18617500	0.78812400	0.61392500
C	4.72132500	-0.43150100	-0.13156300
C	3.65540700	-0.91986700	-1.11906900
H	3.99146800	1.59135200	-0.10788000
H	4.93332100	1.16288600	1.31841700
H	4.95661500	-1.23533600	0.56860700
H	3.46617600	-0.12422100	-1.84921600
H	4.03507800	-1.78313000	-1.66877000
N	1.93932500	-0.26954100	0.54722600
O	0.75419800	-0.41205700	1.07772100
C	1.22682500	-1.42559900	-1.51717200
H	1.53962000	-2.14979800	-2.27186400
H	0.28305800	-1.76853700	-1.09305100
H	1.07799800	-0.46740500	-2.02204500
C	2.44402800	-2.69034900	0.27480800
H	1.52035100	-2.90980900	0.81171200
H	2.60526300	-3.47052800	-0.47206700
H	3.27987500	-2.72490800	0.97228000
C	2.23200500	1.83806300	1.79536400
H	1.35323300	1.67578200	2.41921400
H	2.94736000	2.43405900	2.36568100
H	1.94159800	2.40626200	0.90922400
C	3.18711000	-0.30039100	2.69684900
H	3.76945100	0.32297000	3.37892100
H	2.25388400	-0.57398100	3.19074800
H	3.75697200	-1.20783500	2.50129600
N	5.97662600	-0.11517100	-0.79957200
C	6.89975500	-1.09455800	-1.07803300
C	8.16329400	-0.63993700	-1.77811400
H	8.18285200	0.42879400	-2.00023100
H	9.01870900	-0.88781700	-1.14647900
H	8.27113900	-1.20192700	-2.70775000
O	6.70414800	-2.25782100	-0.76568400
H	6.14386600	0.82633600	-1.11958900
C	-1.89753600	4.19182200	0.53818200
C	-2.01300200	2.84773900	0.83649600
C	-1.25882100	1.90069400	0.10996400
C	-0.39670600	2.35347000	-0.91360100
C	-0.28933800	3.70510000	-1.19060000
C	-1.03545200	4.65366100	-0.47586400
H	-2.48945100	4.90873200	1.09629200
H	-2.70191700	2.54849000	1.61707600
H	0.15644300	1.62492200	-1.49285100
H	0.37064900	4.03464400	-1.98498300
N	-1.30780600	0.52891000	0.29635300
N	-2.07232600	0.01805900	1.24251000
H	-2.51350100	0.59426000	1.95640300
S	-2.70951500	-1.63769300	1.10804500
O	-1.69204300	-2.35077300	0.36109600
O	-3.06342900	-1.91788700	2.48590800
C	-4.17922000	-1.46360500	0.12708100
C	-4.08910900	-1.54648200	-1.26387300
C	-5.40275800	-1.27001800	0.77214400
C	-5.25146500	-1.42298200	-2.01367500
H	-3.13629100	-1.73277800	-1.74236300
C	-6.55069700	-1.14998400	-0.00116100
H	-5.45502700	-1.24597800	1.85341200
C	-6.49656000	-1.21863600	-1.40035300
H	-5.19459400	-1.50276100	-3.09348600
H	-7.50723400	-1.01740600	0.49158900
C	-7.74507900	-1.06878200	-2.22801300
H	-8.63128900	-1.38454000	-1.67518000
H	-7.89098100	-0.02114100	-2.51296900
H	-7.68582000	-1.65239600	-3.14850000
C	-0.95435400	6.11784600	-0.80049400
H	-0.05773700	6.35456000	-1.37467100
H	-1.82079400	6.42484300	-1.39725600
H	-0.95848600	6.72680700	0.10663200

Imaginary frequency: -1007.7415 cm^{-1}

Hydrazide **S16**

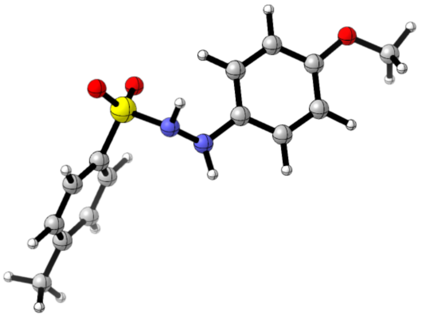

In gas phase

N	0.20625200	0.74703900	-1.10265600
H	-0.37417200	1.46852900	-1.53392800
N	-0.47681300	-0.40928500	-0.70525900
H	-0.26474300	-1.15271400	-1.35806000
C	-1.86814600	-0.32432700	-0.41534100
C	-2.38975300	0.68045200	0.41124700
C	-2.73242100	-1.29167800	-0.92591600
C	-3.74558300	0.71135900	0.69928000
H	-1.72855600	1.41391000	0.85418500
C	-4.09436000	-1.27792300	-0.61772400
H	-2.34684900	-2.07281100	-1.57428000
C	-4.60990400	-0.26645300	0.19359200
H	-4.15482200	1.48436000	1.33853700
H	-4.73275900	-2.04793100	-1.02932500
S	1.20999300	1.49876700	0.07935400
O	1.56227300	2.76613000	-0.55976500
O	0.60332100	1.47049500	1.40937600
C	2.63354000	0.41306500	0.10816900
C	3.60045700	0.53113700	-0.88886300
C	2.75829500	-0.52451100	1.12843900
C	4.70568700	-0.31057600	-0.85679500
H	3.49060400	1.27749600	-1.66573000
C	3.87234200	-1.36022500	1.14215100
H	1.99961900	-0.58790100	1.89772900
C	4.86018200	-1.26729600	0.15618200
H	5.46341600	-0.22074700	-1.62809000
H	3.97445100	-2.09338100	1.93510600
C	6.08156400	-2.15135100	0.19703800
H	6.91840900	-1.63233600	0.67672600
H	5.89391700	-3.06588300	0.76307000
H	6.40505900	-2.43092800	-0.80833500
O	-5.92524800	-0.14515000	0.54802400
C	-6.84456500	-1.11806400	0.07581700
H	-6.58383800	-2.12162600	0.43073900
H	-7.81433700	-0.83419900	0.48149500
H	-6.89813400	-1.12183300	-1.01882100

Transition state of Hydrazide **S16** & **1**

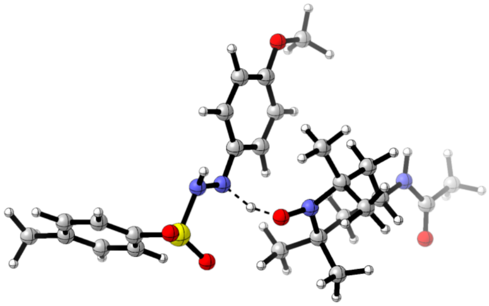

In gas phase

H	-0.09073600	-0.31200000	0.39561700
C	2.30354800	-1.67195800	-0.67564700
C	3.00574000	0.13597200	1.16172400
C	4.24319900	0.40333100	0.28844900
C	4.72186700	-0.82788400	-0.48946500
C	3.60224100	-1.29510400	-1.41480200
H	4.01988500	1.20338000	-0.42691200
H	5.04509400	0.75881400	0.93800700
H	4.98903100	-1.61672100	0.21669400
H	3.38245100	-0.49793000	-2.13624900
H	3.92043300	-2.16908600	-1.98933600
N	1.98788500	-0.61903100	0.35414000
O	0.83259400	-0.75507700	0.94545100
C	1.14337100	-1.73051700	-1.68373600
H	1.39779000	-2.44906900	-2.46565700
H	0.21698900	-2.05941400	-1.21343900
H	0.98817400	-0.76046600	-2.16377800
C	2.42126100	-3.04428100	0.02925800
H	1.52083100	-3.24663900	0.61014600
H	2.52297500	-3.82269900	-0.73038700
H	3.28741700	-3.10156800	0.68772600
C	2.38382200	1.47224100	1.59831200
H	1.53840800	1.31611600	2.26872900
H	3.13946700	2.05150500	2.13246300
H	2.05467400	2.05420200	0.73483300
C	3.36410200	-0.68459900	2.42550600
H	4.00081800	-0.07551400	3.07033400
H	2.45923600	-0.94688500	2.97559900
H	3.91037700	-1.59716800	2.19119800
N	5.93297800	-0.55114400	-1.25125100
C	7.17034300	-0.58743300	-0.65625700
C	8.35245200	-0.29346700	-1.55620900
H	9.01250900	-1.16328900	-1.55778000
H	8.07380200	-0.05667100	-2.58489200
H	8.91519600	0.54180700	-1.13523900
O	7.29657100	-0.85068300	0.52892100
H	5.85843900	-0.29038400	-2.22238000
C	-1.86676200	3.88168200	0.71003500
C	-1.94873800	2.52408200	0.95470100
C	-1.22603500	1.60444400	0.16414600
C	-0.41547400	2.10398000	-0.88928000
C	-0.32392600	3.45285500	-1.12808900
C	-1.04522300	4.36499000	-0.32935900
H	-2.44276900	4.56369300	1.31981500
H	-2.60012600	2.19208600	1.75374100
H	0.11301700	1.39948600	-1.51908200
H	0.28236700	3.84348200	-1.93554800
N	-1.25580100	0.23401700	0.30788700
N	-1.97062900	-0.32075600	1.27970200
H	-2.37725600	0.22860000	2.03298200
S	-2.66723900	-1.93352000	1.07578500
O	-1.70013800	-2.64500600	0.26182400
O	-2.99632800	-2.27960900	2.44542400
C	-4.16005700	-1.66949300	0.14676200
C	-4.11038600	-1.67310300	-1.24813900
C	-5.35925600	-1.47705000	0.83577400
C	-5.28838100	-1.47309800	-1.95680800
H	-3.17554500	-1.85224200	-1.76337800
C	-6.52371800	-1.27984200	0.10389600
H	-5.38029200	-1.50776500	1.91795900
C	-6.51035800	-1.27449700	-1.29789600
H	-5.26154600	-1.48343100	-3.04073500
H	-7.46113600	-1.14067300	0.63065300
C	-7.78601400	-1.10226000	-2.07883200
H	-8.24941100	-2.07662300	-2.26749600
H	-8.51117200	-0.49623000	-1.53281700
H	-7.60259000	-0.63460300	-3.04787900
O	-0.89801400	5.65446900	-0.64312200
C	-1.59555700	6.66607100	0.10121800
H	-1.29424200	6.65042200	1.15139800
H	-1.30091500	7.60927700	-0.35103200
H	-2.67669100	6.53577000	0.01067500

Imaginary frequency: -1083.9305 cm^{-1}

Hydrazide **S12**

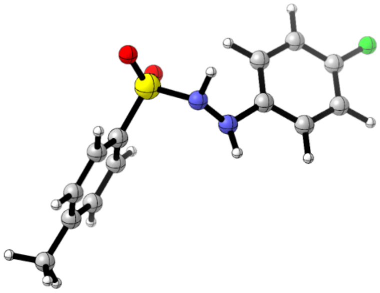

In gas phase

N	-0.20913000	0.73263300	-1.11370800
H	-0.80205600	1.44823800	-1.53741600
N	-0.84958100	-0.46150200	-0.78636700
H	-0.59510700	-1.17782700	-1.45320600
C	-2.22832900	-0.46685900	-0.45796300
C	-2.81752900	0.55587900	0.29279000
C	-3.00963200	-1.55570000	-0.86571400
C	-4.17117500	0.49440700	0.61697400
H	-2.21782100	1.37690400	0.66141200
C	-4.35743400	-1.62894200	-0.52921000
H	-2.56280900	-2.35259000	-1.45163100
C	-4.91812800	-0.59565500	0.20423500
H	-4.63880200	1.27779900	1.20025300
H	-4.96980000	-2.46622000	-0.83970300
S	0.74637500	1.47341500	0.11716900
O	1.04884900	2.78100700	-0.46222700
O	0.11496400	1.35722400	1.43022800
C	2.21460300	0.45022400	0.12380200
C	3.18619800	0.64604700	-0.85665200
C	2.36853700	-0.51738400	1.11170700
C	4.32672900	-0.14736700	-0.84008000
H	3.05300000	1.41443900	-1.60797600
C	3.51828300	-1.30326100	1.11013800
H	1.60546700	-0.64095600	1.86929100
C	4.51193500	-1.13202500	0.14068000
H	5.08836800	0.00294900	-1.59792800
H	3.64403200	-2.05878200	1.87822100
C	5.77054700	-1.96255500	0.16601800
H	6.58129400	-1.41973100	0.66374400
H	5.62051300	-2.89869300	0.70724400
H	6.11093300	-2.20135100	-0.84427900
F	-6.23626400	-0.65593600	0.52593700

Transition state of hydrazide **S12** & **1**

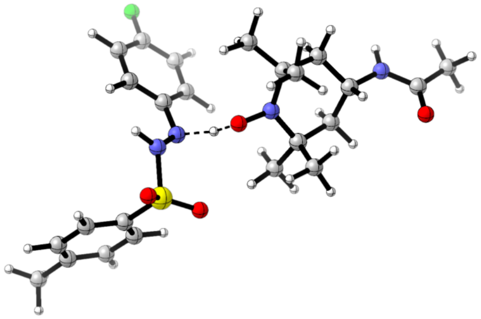

In gas phase

H	-0.11812800	0.01110900	0.43141900
C	2.33120800	-1.31493100	-0.45136000
C	2.89445400	0.53936800	1.39104000
C	4.19651000	0.79683900	0.60791500
C	4.72665400	-0.43836400	-0.11488500
C	3.66669200	-0.92709100	-1.10862000
H	4.01851400	1.59231400	-0.12657300
H	4.93938800	1.17347900	1.31585800
H	4.94341600	-1.23580700	0.59855800
H	3.49392700	-0.13810200	-1.84991900
H	4.04345600	-1.80056000	-1.64385000
N	1.94068200	-0.24300400	0.53387900
O	0.74955800	-0.36819300	1.05604000
C	1.23703200	-1.41045500	-1.52761600
H	1.55010100	-2.14537700	-2.27167000
H	0.28474600	-1.73929000	-1.11148500
H	1.10544700	-0.45587700	-2.04425700
C	2.42195400	-2.67054300	0.29035300
H	1.48927400	-2.87708100	0.81665100
H	2.58684300	-3.45906700	-0.44683900
H	3.24811000	-2.70485500	0.99927700
C	2.23873000	1.88300700	1.75005000
H	1.35096200	1.73927400	2.36570300
H	2.95255900	2.48144600	2.31952200
H	1.96629600	2.44034300	0.85129300
C	3.16468500	-0.25016900	2.69612200
H	3.74815100	0.37754500	3.37314400
H	2.22424500	-0.50546100	3.18607500
H	3.72590900	-1.16686200	2.51957900
N	5.99308600	-0.14548700	-0.77191600
C	6.90139300	-1.14278700	-1.03648200
C	8.18145100	-0.71447800	-1.72238000
H	8.22264900	0.35244800	-1.94994000
H	9.02411400	-0.97370200	-1.07824500
H	8.29152700	-1.28354600	-2.64741200
O	6.67901700	-2.30088800	-0.72277000
H	6.18182900	0.79254700	-1.09018200
C	-1.87669700	4.23509300	0.49629800
C	-1.99444900	2.88950500	0.79279100
C	-1.25234900	1.93624500	0.06156000
C	-0.39590800	2.37177200	-0.97594800
C	-0.27067700	3.71781800	-1.26853800
C	-1.01225300	4.63003100	-0.52453100
H	-2.44375100	4.98432300	1.03398700
H	-2.68049400	2.59662700	1.57761400
H	0.14000500	1.63297700	-1.55766100
H	0.36985900	4.07047400	-2.06672600
N	-1.31031800	0.56384100	0.25360200
N	-2.07383700	0.06168100	1.20149200
H	-2.49352600	0.63481800	1.93110800
S	-2.69908300	-1.60594400	1.09262900
O	-1.68849000	-2.31171500	0.33002500
O	-3.01922400	-1.87647700	2.48023600
C	-4.18874500	-1.44846400	0.14242700
C	-4.12617000	-1.53464500	-1.25000400
C	-5.39933300	-1.25387400	0.81213700
C	-5.30390300	-1.41678500	-1.97617000
H	-3.18187400	-1.71353100	-1.74787600
C	-6.56267000	-1.13942200	0.06202700
H	-5.42892600	-1.21926500	1.89397600
C	-6.53734600	-1.21812300	-1.33778400
H	-5.26801700	-1.49174000	-3.05719000
H	-7.50840400	-0.99860100	0.57304500
C	-7.81059500	-1.13352200	-2.13574000
H	-8.24049600	-2.13224900	-2.26845100
H	-8.55935100	-0.52013500	-1.63158100
H	-7.63383400	-0.71919600	-3.12986800
F	-0.90570800	5.93094600	-0.80586400

Imaginary frequency: -962.8151 cm^{-1}

References

1. Zhao, Y.; Li, S.; Cui, J.; Wang, H.; Kang, X.; Wang, Y.; Tian, L. *Synthesis* **2022**, *54*, 5245.
2. Jiménez-Aberásturi, X.; Palacios, F.; de los Santos, J. M. *J. Org. Chem.* **2022**, *87*, 11583.
3. Dey, S.; Panja, D.; Sau, A.; Thakur, S. D.; Kundu, S. *J. Org. Chem.* **2023**, *88*, 10048.
4. Kim, M. H.; Kim, J. *J. Org. Chem.* **2018**, *83*, 1673.
5. Zhang, Y.; Huang, C.; Lin, X.; Hu, Q.; Hu, B.; Zhou, Y.; Zhu, G. *Org. Lett.* **2019**, *21*, 2261.
6. Hannelore, J.; Hoefling, S. B.; Heinrich, M. *J. Org. Chem.* **2012**, *77*, 1520.
7. Tayama, E.; Kobayashi, Y.; Toma, Y. *Chem. Commun.* **2016**, *52*, 10570.
8. Moloney, G. P.; Martin, G. R.; Mathews, N.; Milne, A.; Hobbs, H.; Dodsworth, S.; Sang, P. Y.; Knight, C.; Williams, M.; Maxwell, M.; Glen, R. C. *J. Med. Chem.* **1999**, *42*, 2504.
9. Liu, J.; Chen, F.; Liu, E.; Li, J.; Qui, G. *New. J. Chem.* **2015**, *39*, 7773.
10. Ito, M.; Tanaka, A.; Higuchi, K.; Sugiyama, S. *Eur. J. Org. Chem*. **2017**, 1272.
11. Anslyn, E. V.; Dougherty, D. A. Modern Physical Organic Chemistry; University Science Books: Mill Valley, CA, 2006; p 446.
12. Gaussian 09, Revision D.01: Frisch, M. J.; Trucks, G. W.; Schlegel, H. B.; Scuseria, G. E.; Robb, M. A.; Cheeseman, J. R.; Scalmani, G.; Barone, V.; Mennucci, B.; Petersson, G. A.; Nakatsuji, H.; Caricato, M.; Li, X.; Hratchian, H. P.; Izmaylov, A. F.; Bloino, J.; Zheng, G.; Sonnenberg, J. L.; Hada, M.; Ehara, M.; Toyota, K.; Fukuda, R.; Hasegawa, J.; Isheda, M.; Nakajima, T.; Honda, Y.; Kitao, O.; Nakai, H.; Vreven, T.; Montgomery, J. A., Jr.; Peralta, J. E.; Ogliaro, F.; Bearpark, M.; Heyd, J. J.; Brothers, E.; Kudin, K. N.; Staroverov, V. N.; Kobayashi, R.; Normand, J.; Raghavachari, K.; Rendell, A.; Burant, J. C.; Iyengar, S. S.; Tomasi, J.; Cossi, M.; Rega, N.; Millam, N. J.; Klene, M.; Knox, J. E.; Cross, J. B.; Bakken, V.; Adamo, C.; Jaramillo, J.; Gomperts, R.; Stratmann, R. E.; Yazyev, O.; Austin, A. J.; Cammi, R.; Pomelli, C.; Ochterski, J. W.; Martin, R. L.; Morokuma, K.; Zakrzewski, V. G.; Voth, G. A.; Salvador, P.; Dannenberg, J. J.; Dapprich, S.; Daniels, A. D.; Farkas, Ő.; Foresman, J. B.; Ortiz, J. V.; Cioslowski, J.; Fox, D. J. Gaussian, Inc.: Wallingford, CT, 2013.
13. Becke, A. D. *J. Chem. Phys.* **1993**, *98*, 5648.
14. Stephens, P. J.; Devlin, F. J.; Chabalowski, C. F.; Frisch, M. J. *J. Phys. Chem.* **1994***, 98,* 11623.
15. Grimme, S.; Antony, J.; Ehrlich, S.; Krieg, H. *J. Chem. Phys.,* **2010**, *132*, 154104.

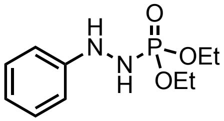

S9

^{1}H NMR

400 MHz, $CDCl_3$

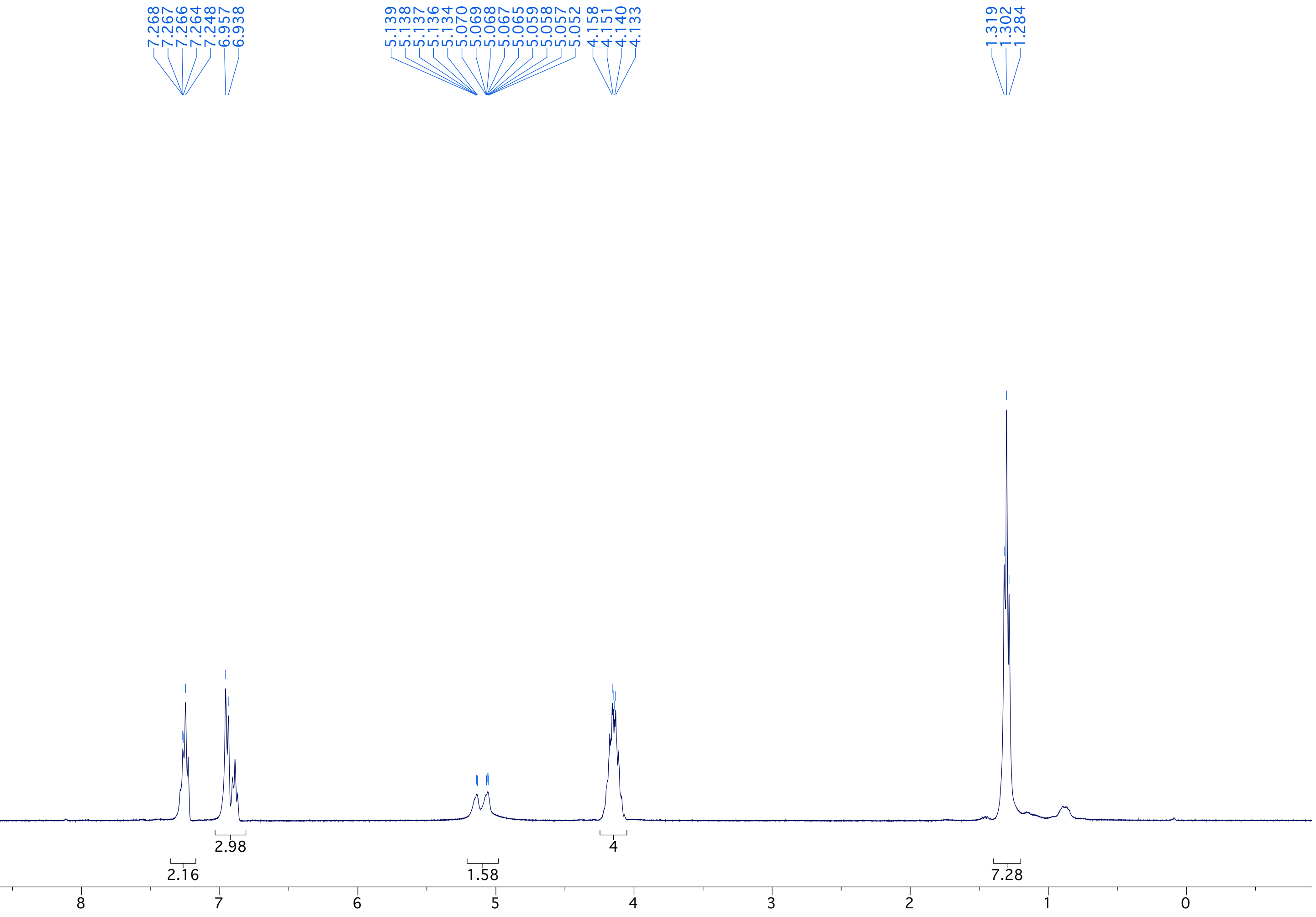

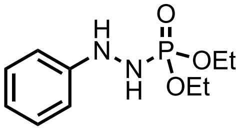

S9

$^{13}C\{^{1}H\}$ NMR

100 MHz, $CDCl_3$

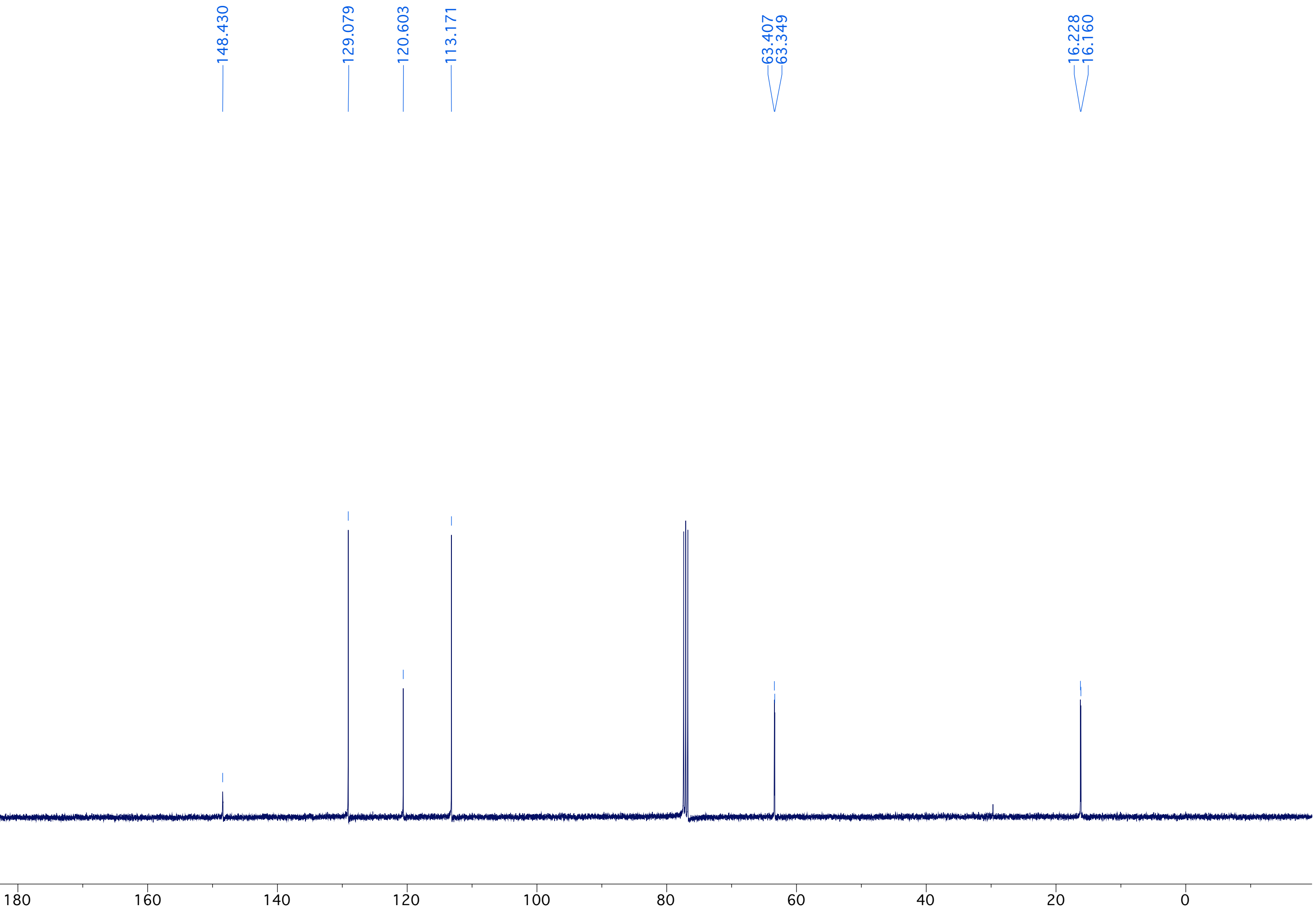

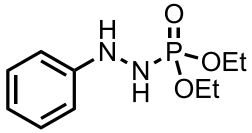

S9

$^{31}P\{^1H\}$ NMR

162 MHz, $CDCl_3$

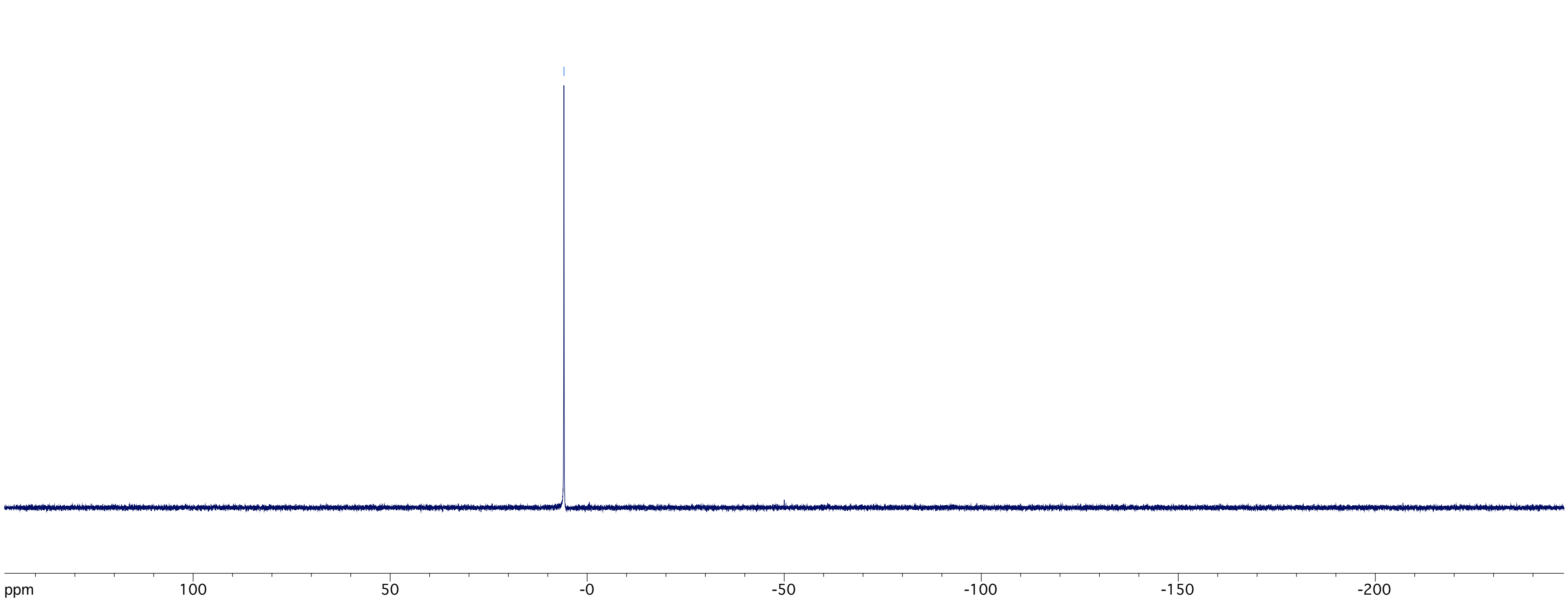

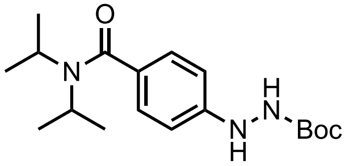

S18

^1H NMR

400 MHz, $CDCl_3$

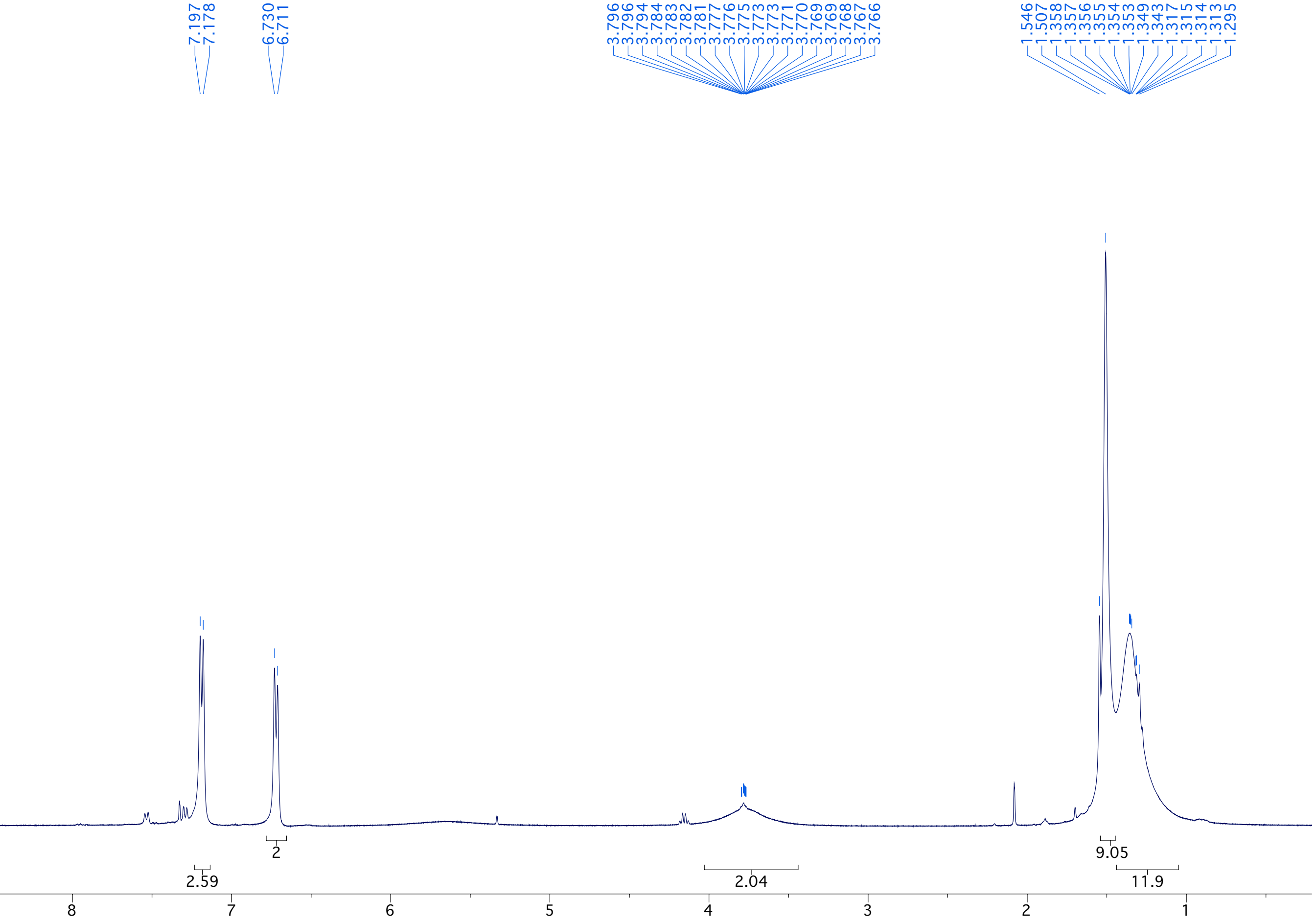

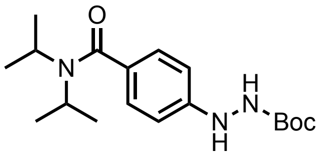

S18
$^{13}C\{^1H\}$ NMR
100 MHz, $CDCl_3$

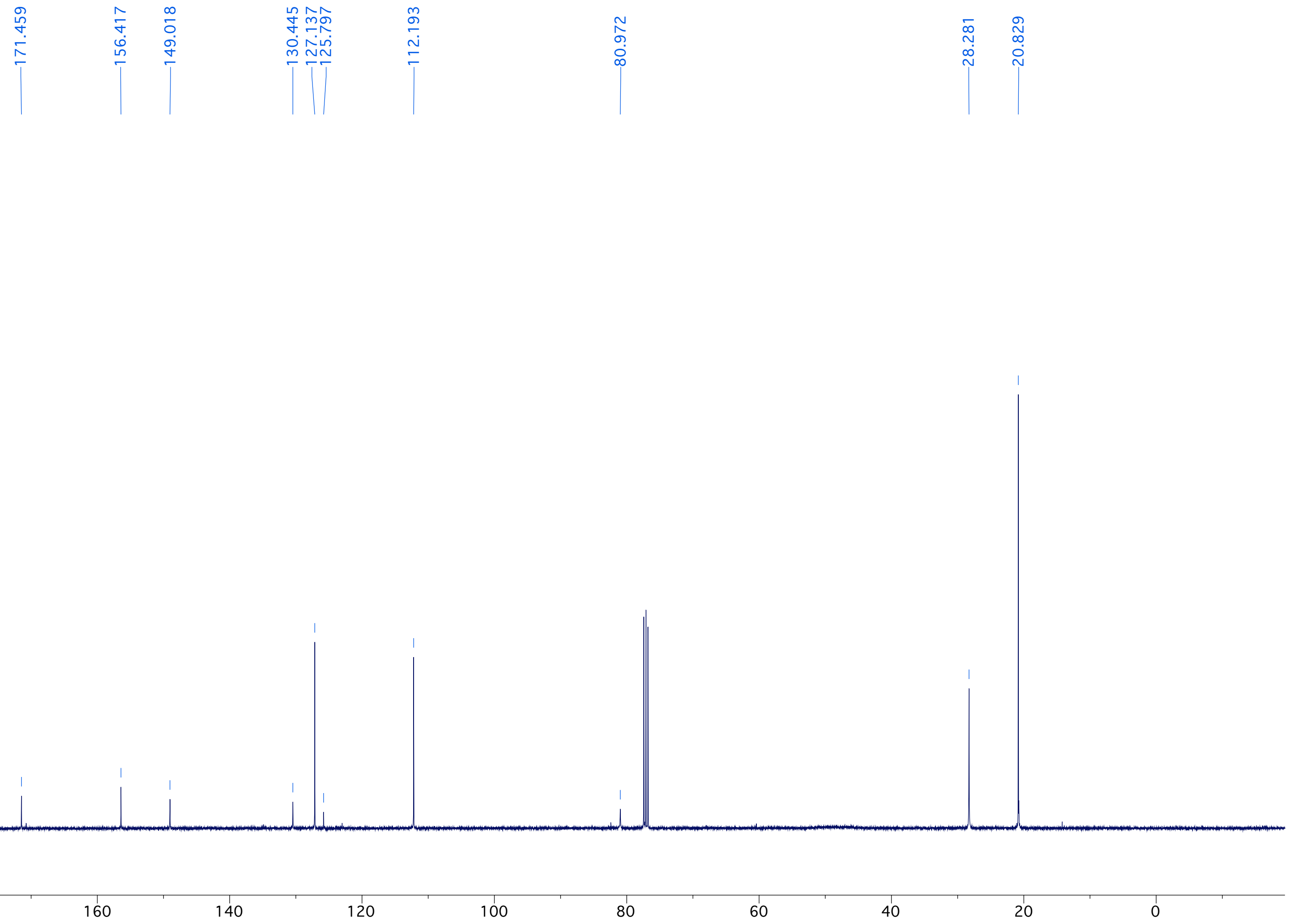

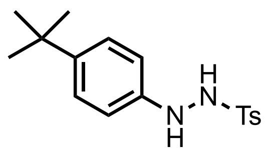

S19

^{1}H NMR

400 MHz, $CDCl_3$

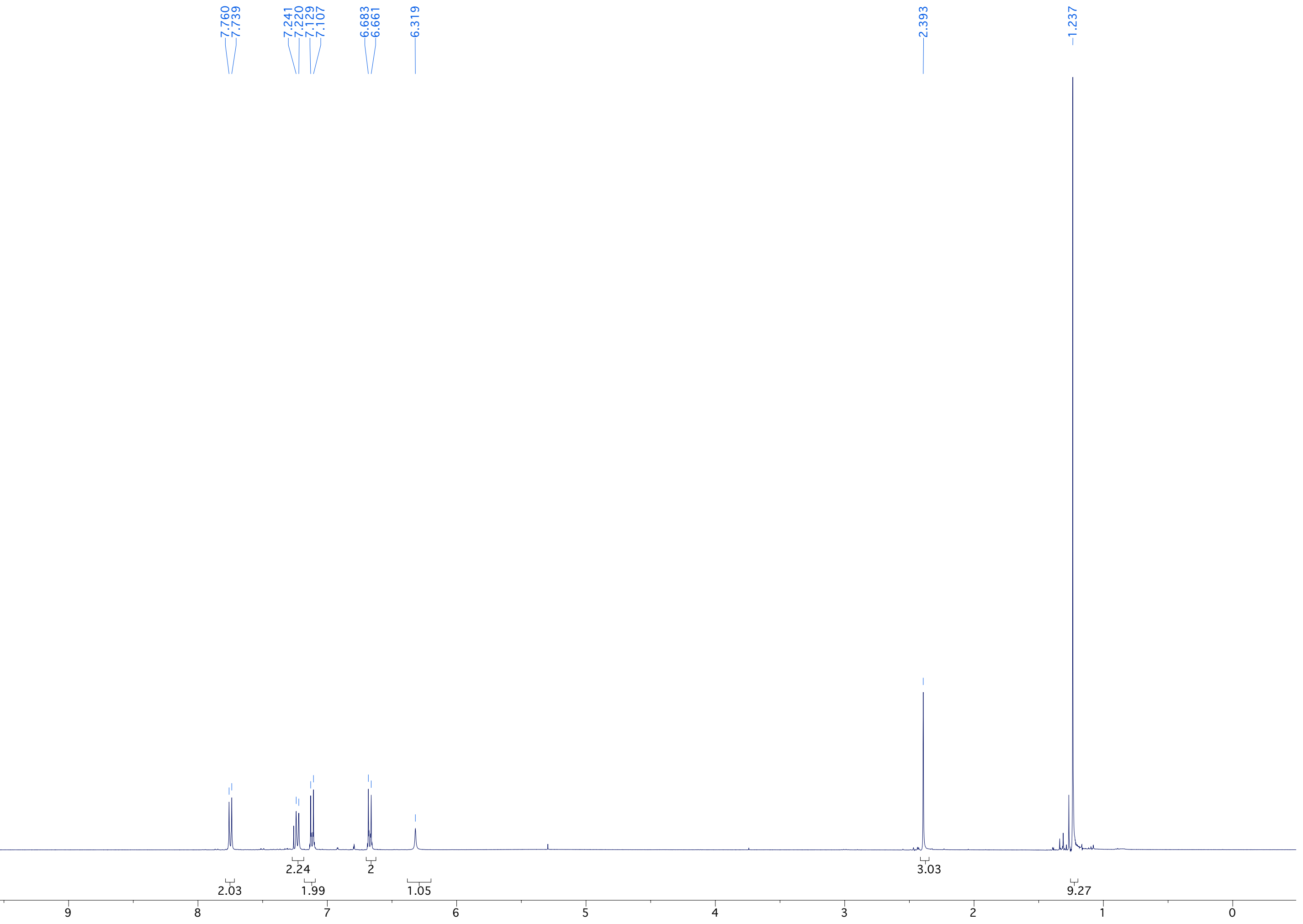

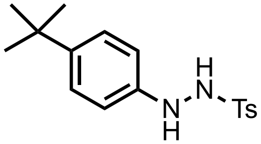

S19

$^{13}C\{^{1}H\}$ NMR

100 MHz, $CDCl_3$

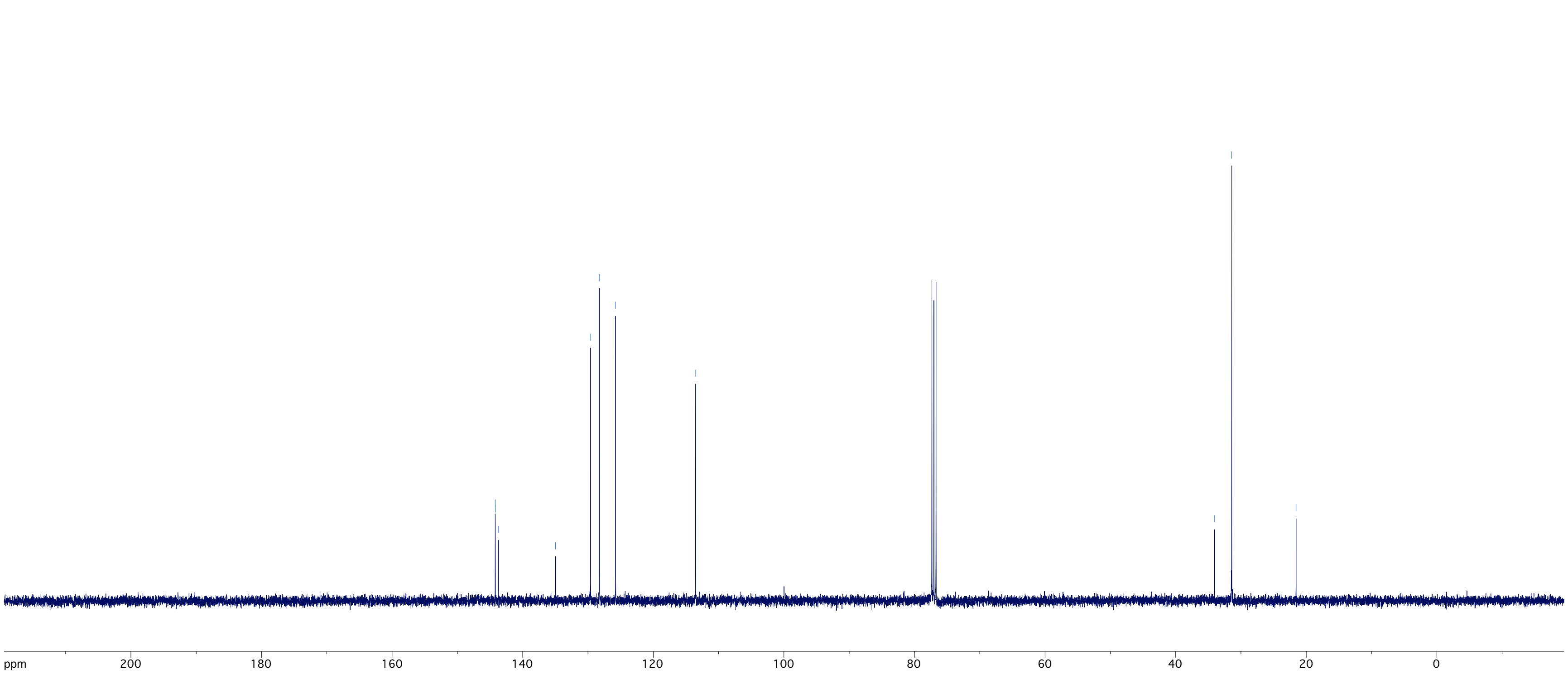

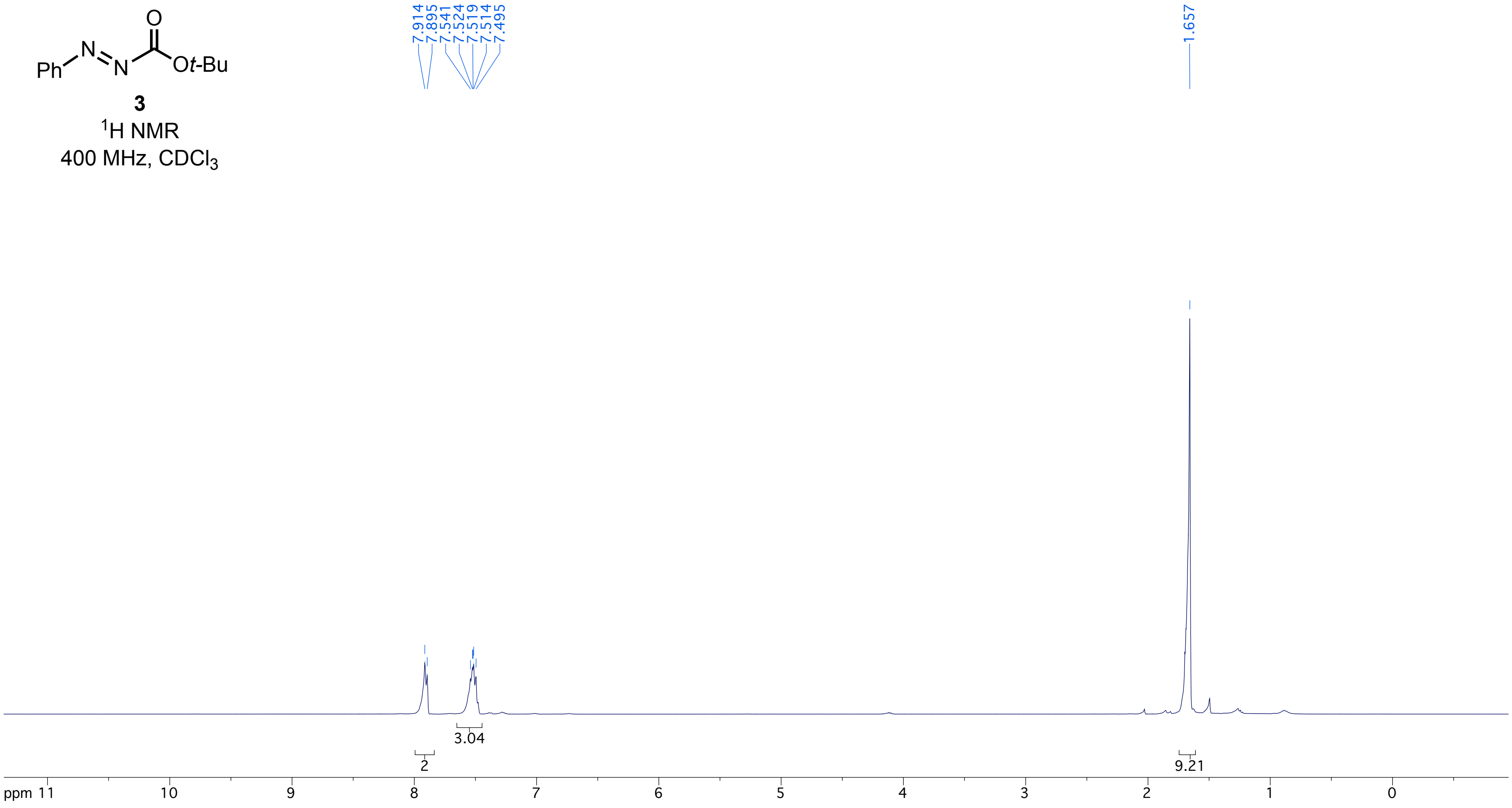
Ph
N
N
O
Ot-Bu
3
^{1}H NMR
400 MHz, CDCl$_3$
7.914
7.895
7.541
7.524
7.519
7.514
7.495
1.657
2
3.04
9.21
ppm 11
10
9
8
7
6
5
4
3
2
1
0

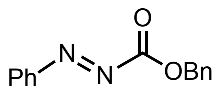

4

^{1}H NMR

400 MHz, $CDCl_3$

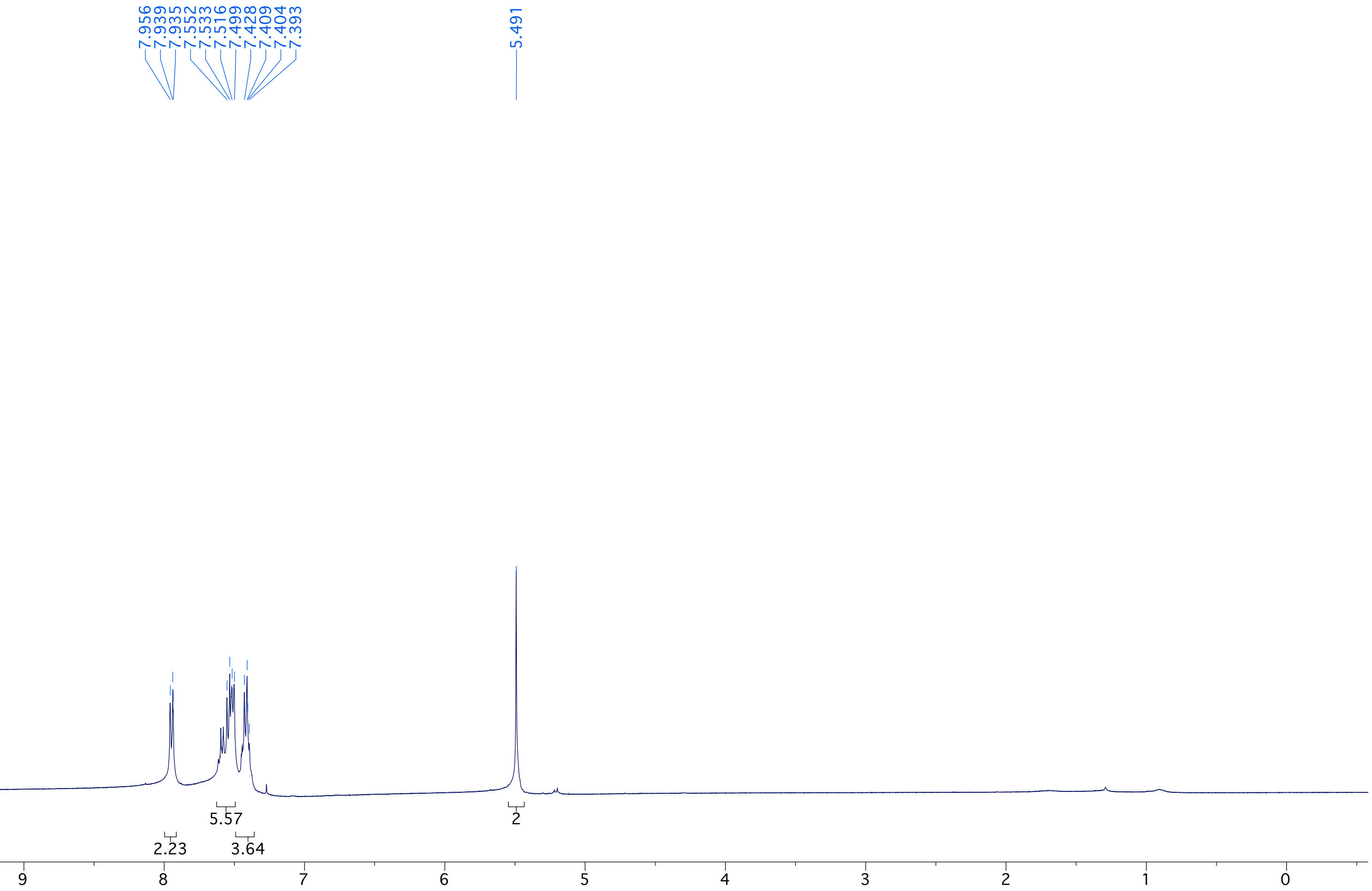

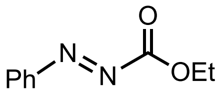

5

^{1}H NMR

400 MHz, $CDCl_3$

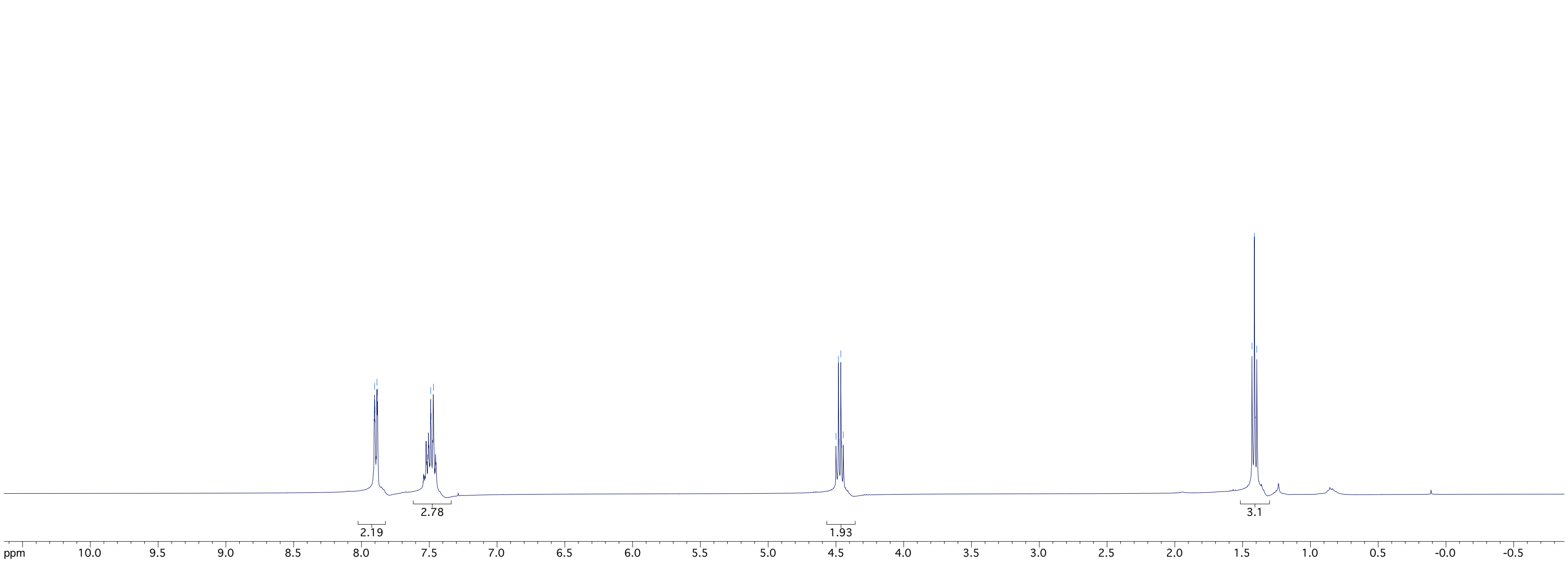

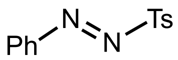

6

^{1}H NMR

400 MHz, $CDCl_3$

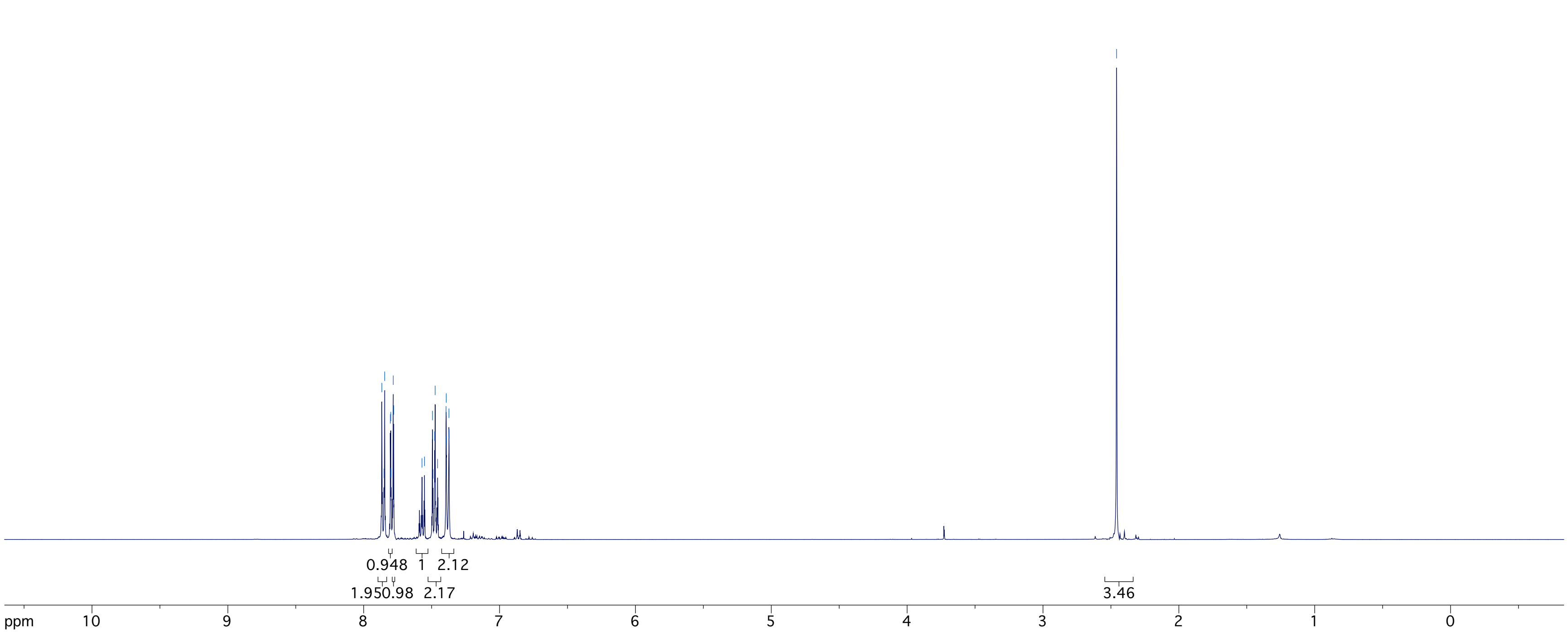

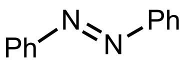

7
^1H NMR
400 MHz, $CDCl_3$

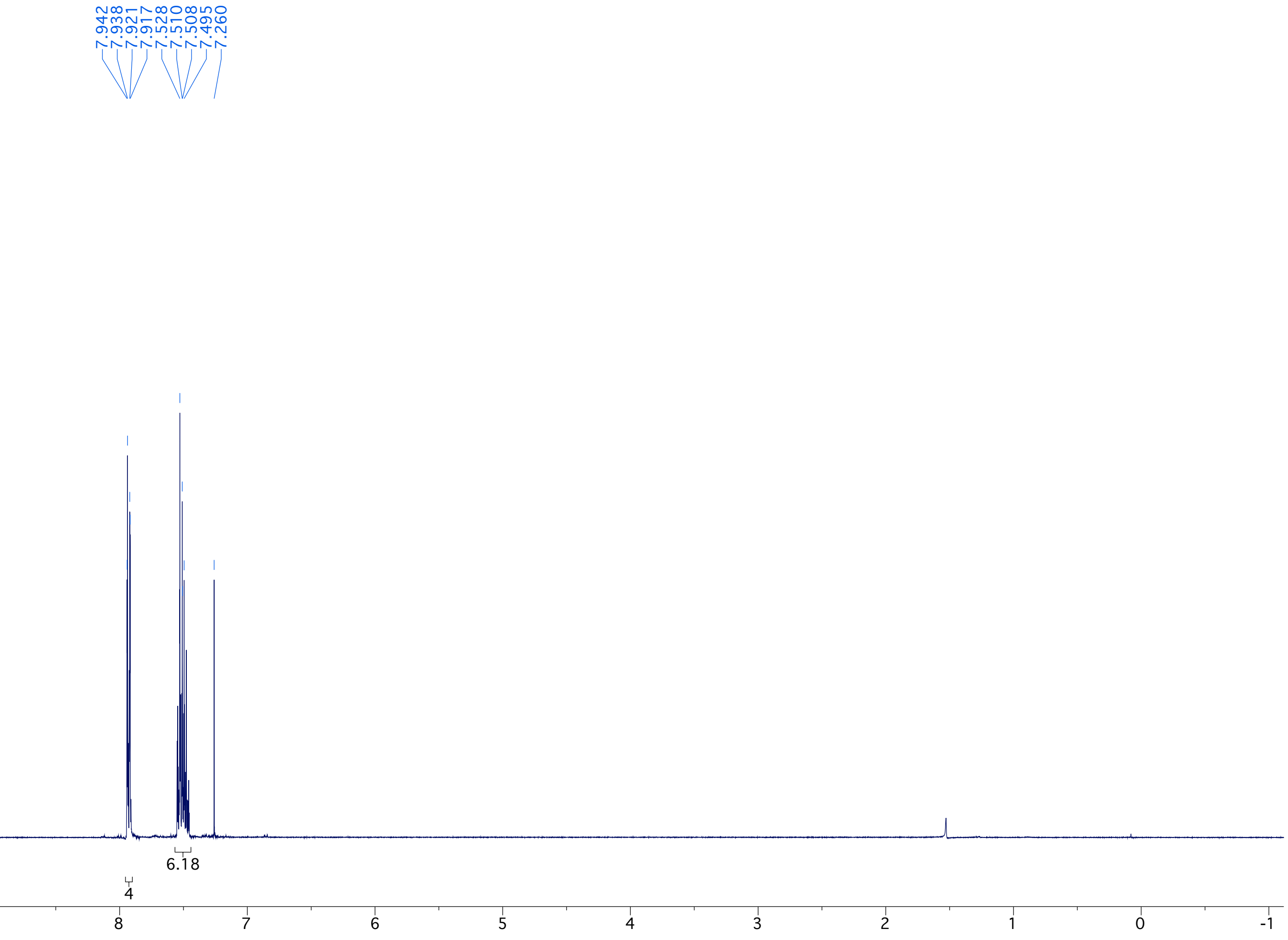

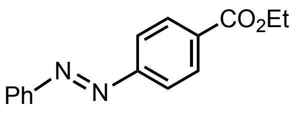

8

^{1}H NMR

400 MHz, $CDCl_3$

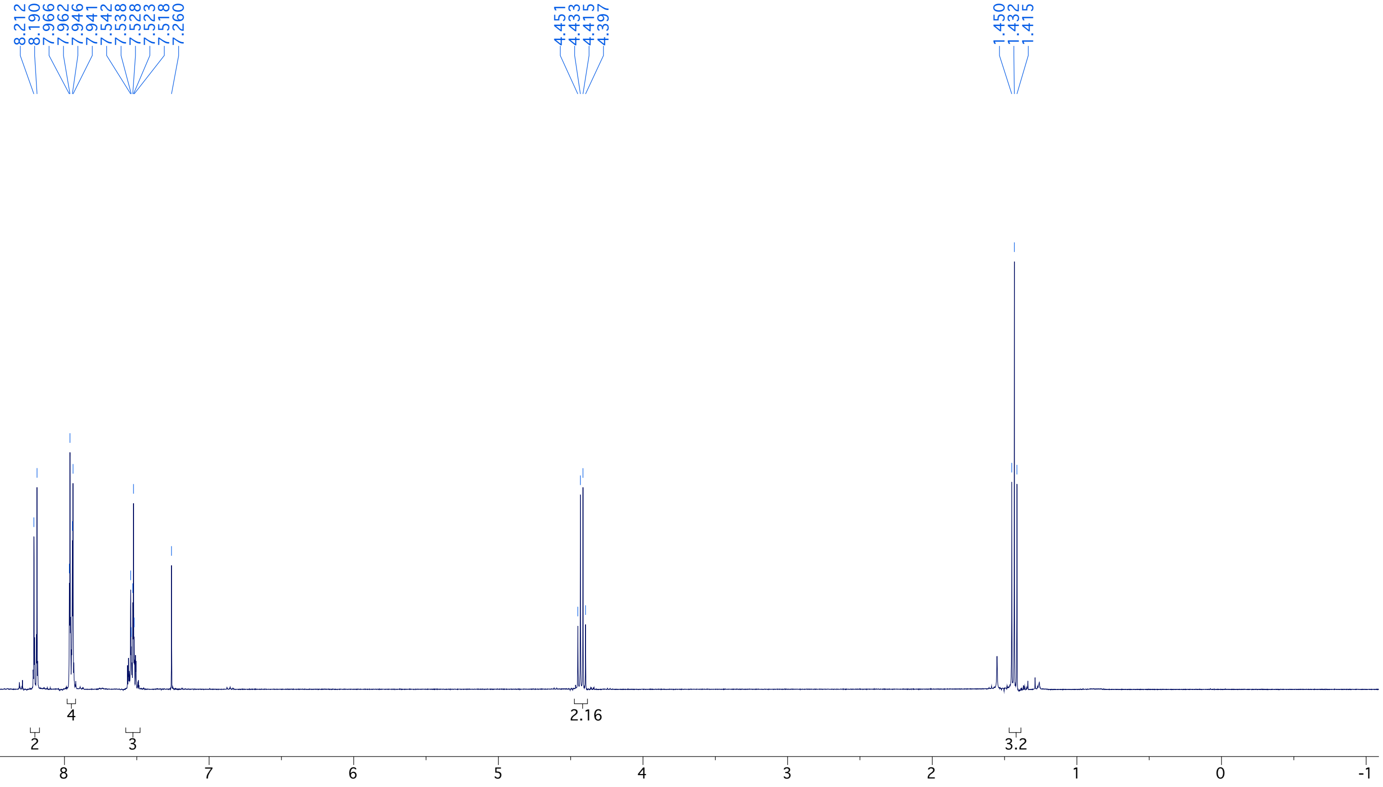

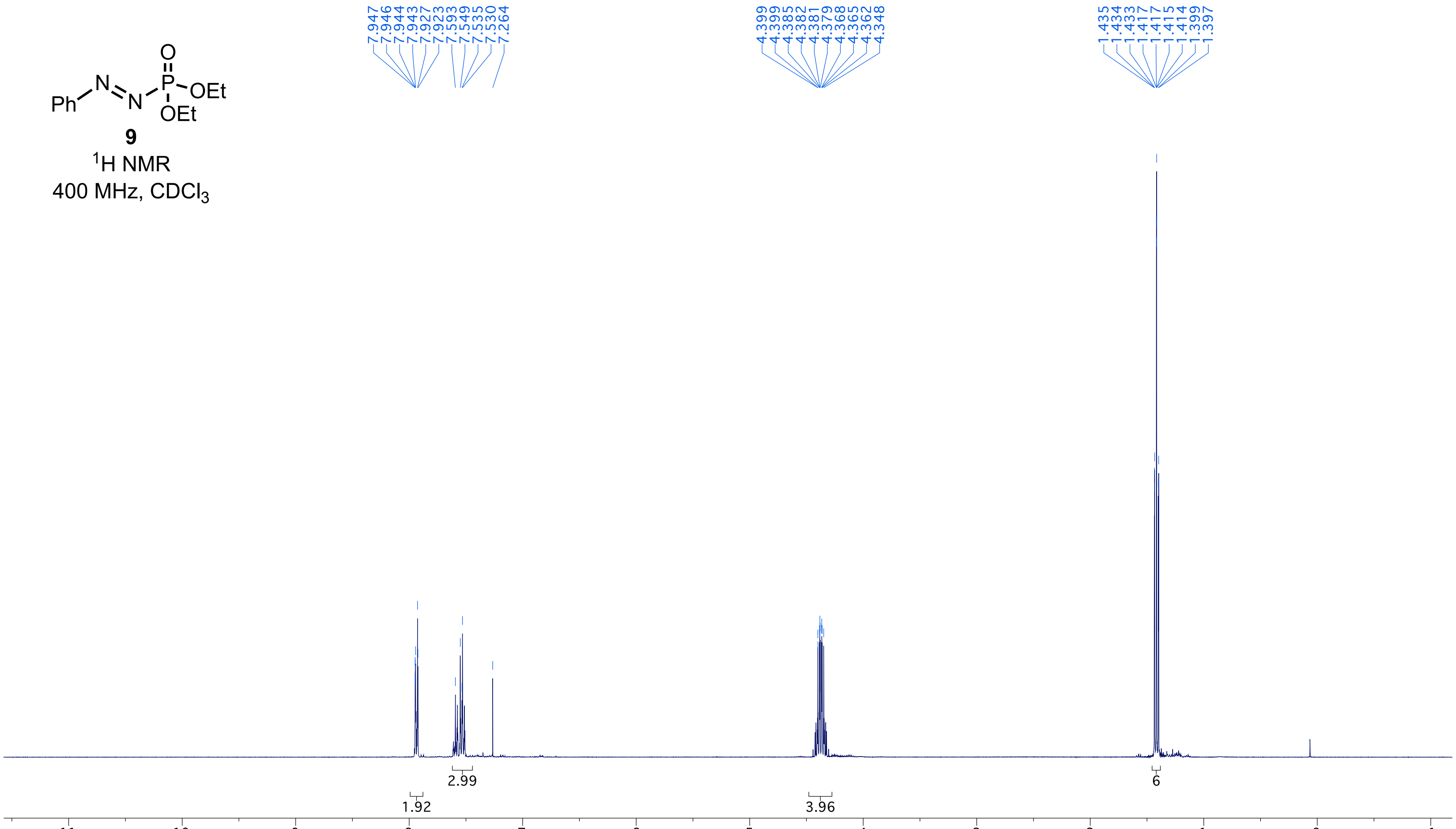

Ph
N
N
P
O
OEt
OEt
9
¹H NMR
400 MHz, CDCl3
7.947
7.946
7.944
7.943
7.927
7.923
7.593
7.549
7.535
7.530
7.264
4.399
4.399
4.385
4.382
4.381
4.379
4.368
4.365
4.362
4.348
1.435
1.434
1.433
1.417
1.417
1.415
1.414
1.399
1.397
2.99
6
1.92
3.96
ppm
11
10
9
8
7
6
5
4
3
2
1
0
-1

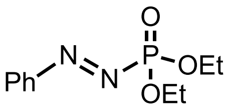

9

$^{13}C\{^1H\}$ NMR
100 MHz, $CDCl_3$

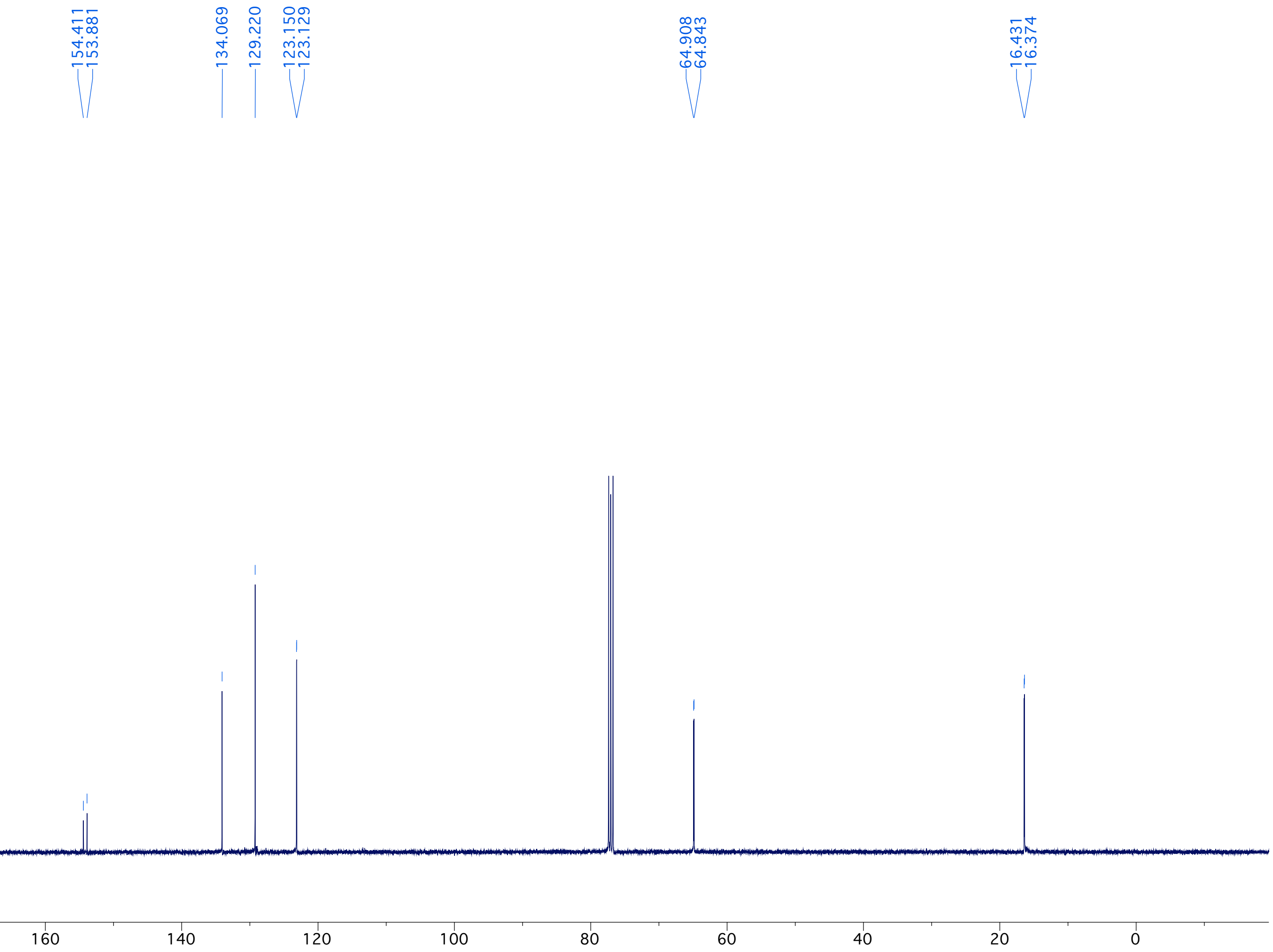

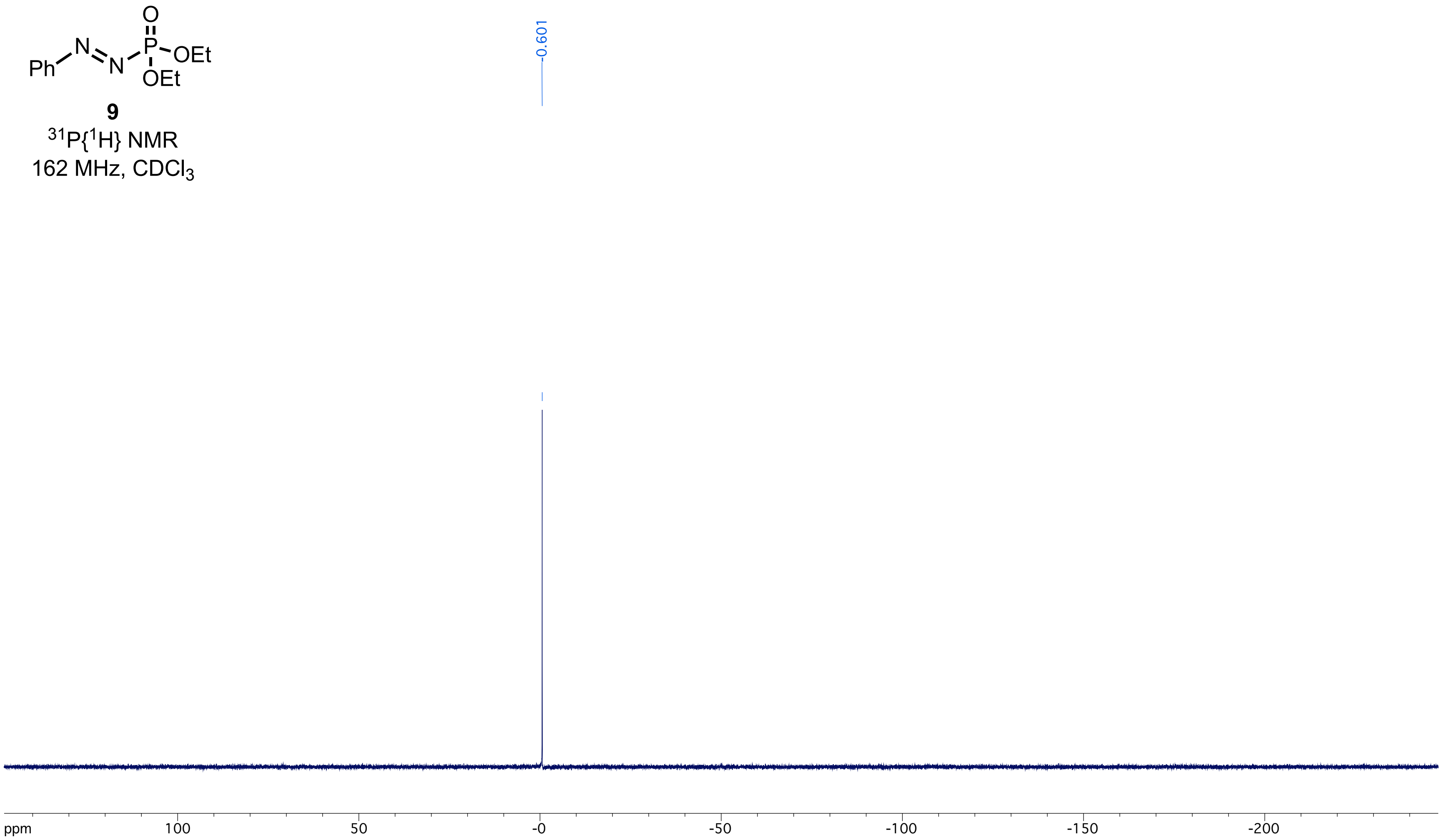

O
Ph
N
N
P
OEt
OEt
9
$^{31}P\{^1H\}$ NMR
162 MHz, $CDCl_3$
-0.601
ppm
100
50
-0
-50
-100
-150
-200

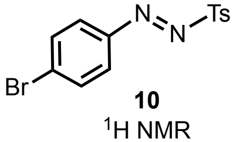

10

^{1}H NMR

400 MHz, $CDCl_3$

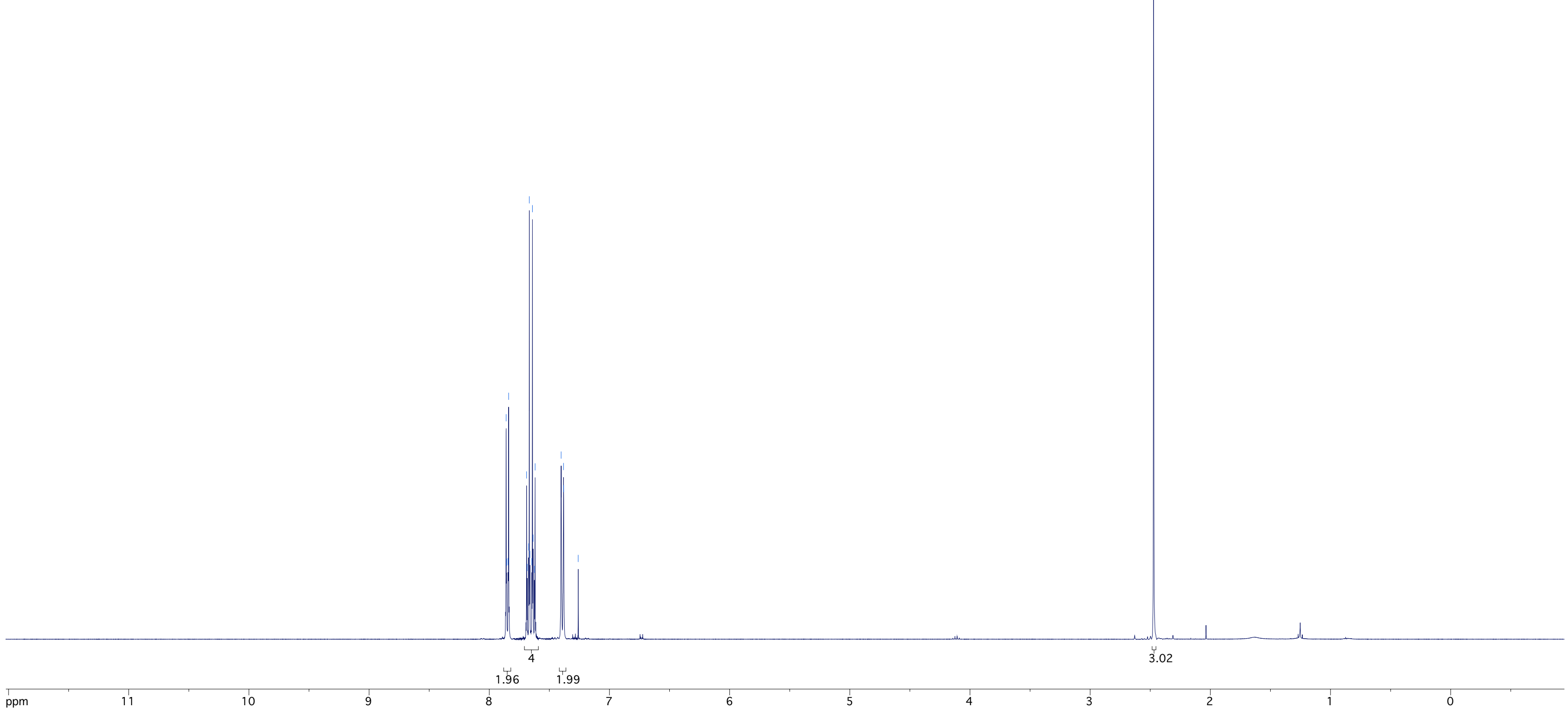

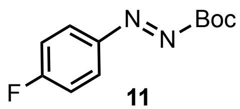

11

^1H NMR

400 MHz, $CDCl_3$

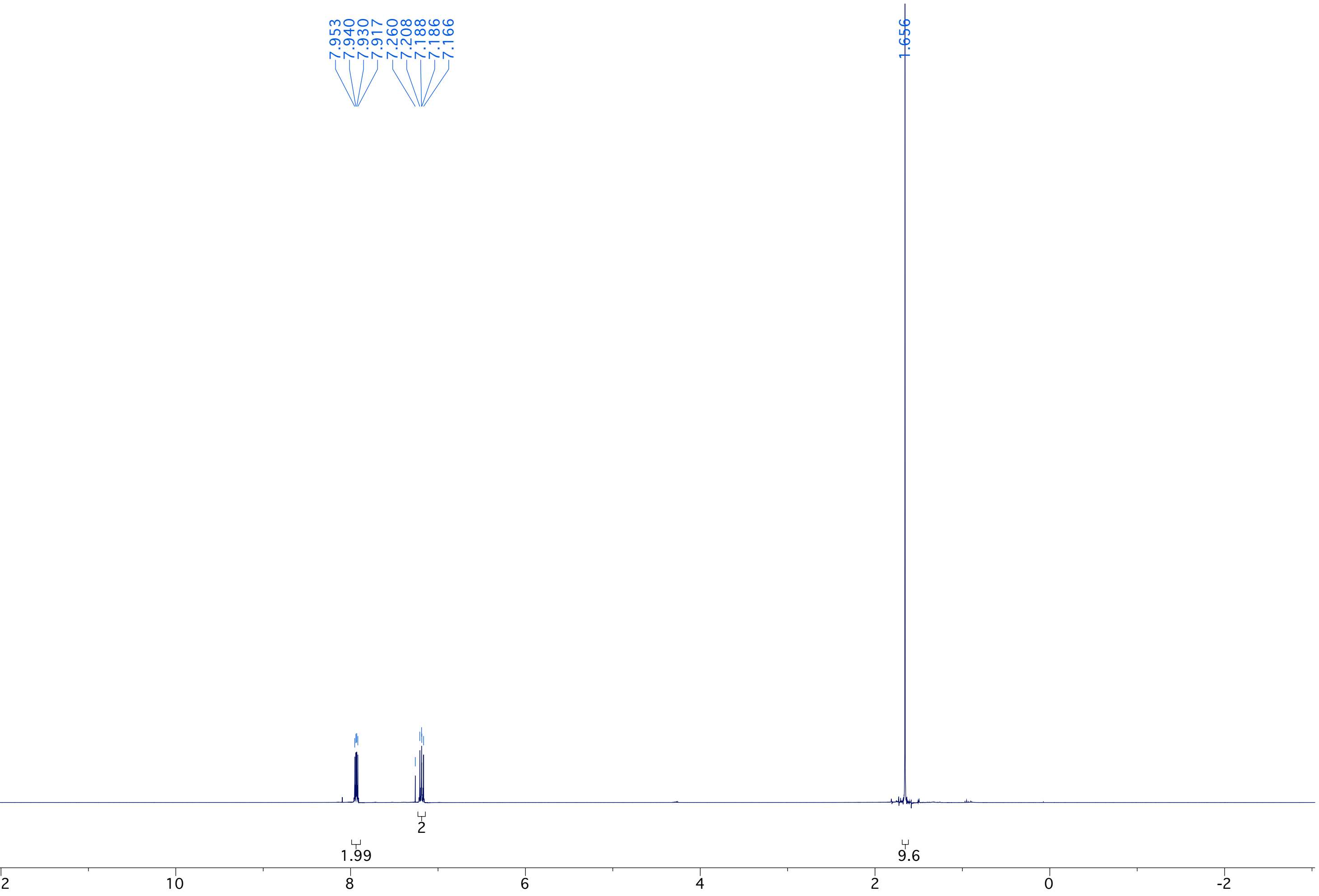

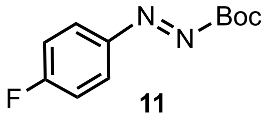

11

$^{19}F\ \{^{1}H\}$ NMR

376 MHz, $CDCl_3$

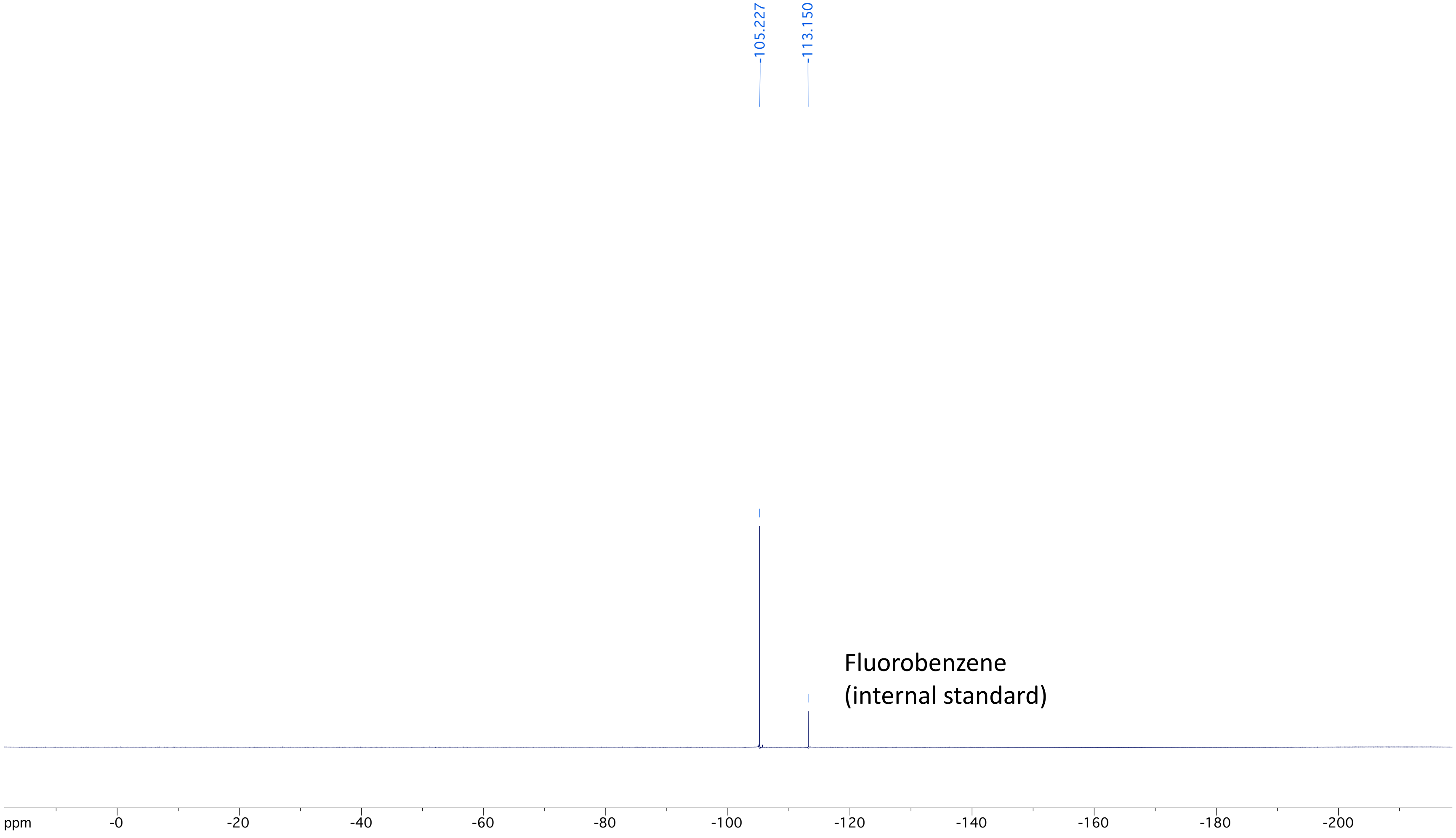

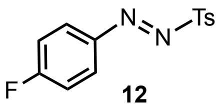

12

^{1}H NMR

376 MHz, $CDCl_3$

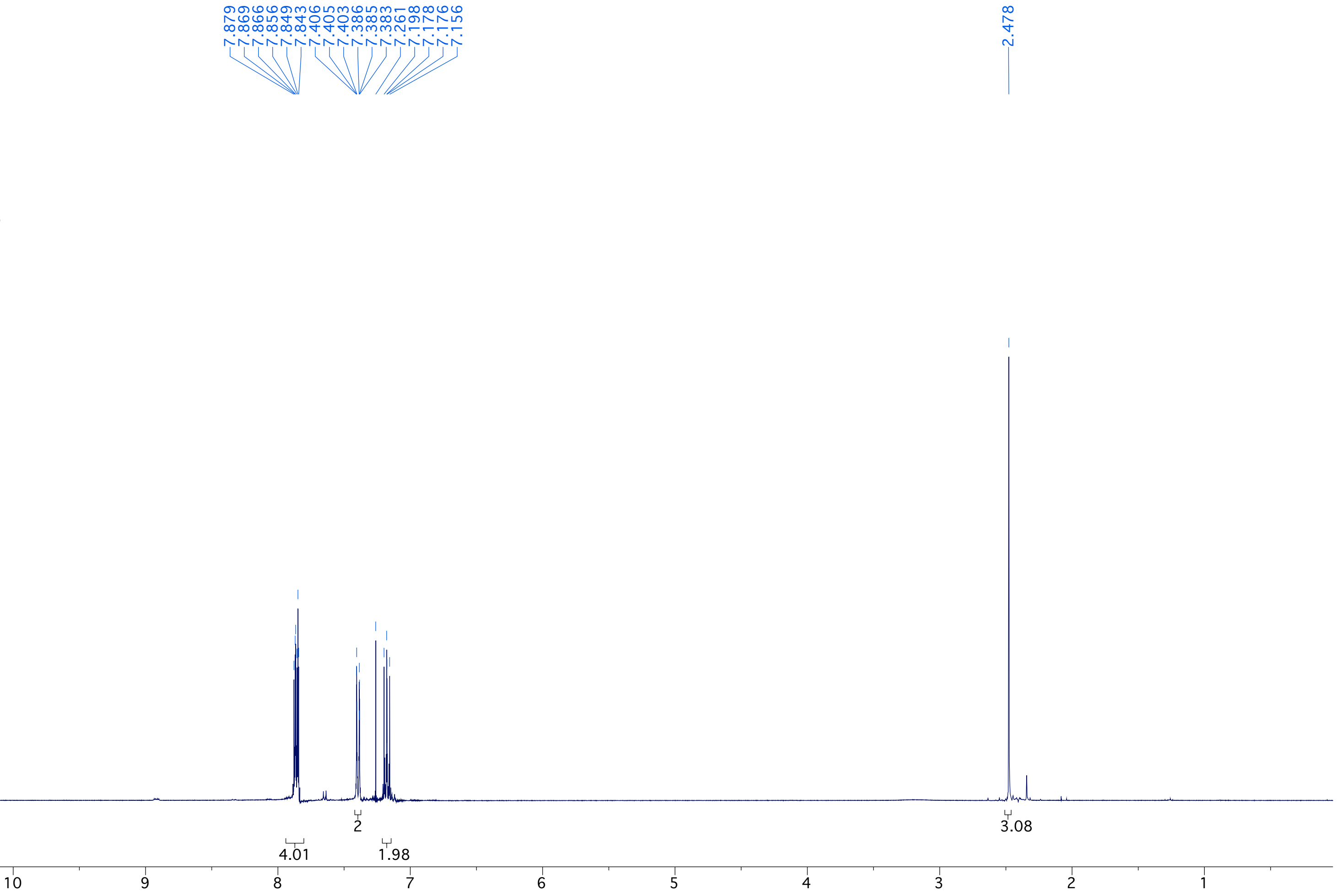

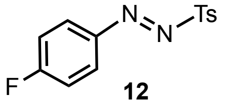

12

$^{19}F\ \{^{1}H\}$ NMR

376 MHz, $CDCl_3$

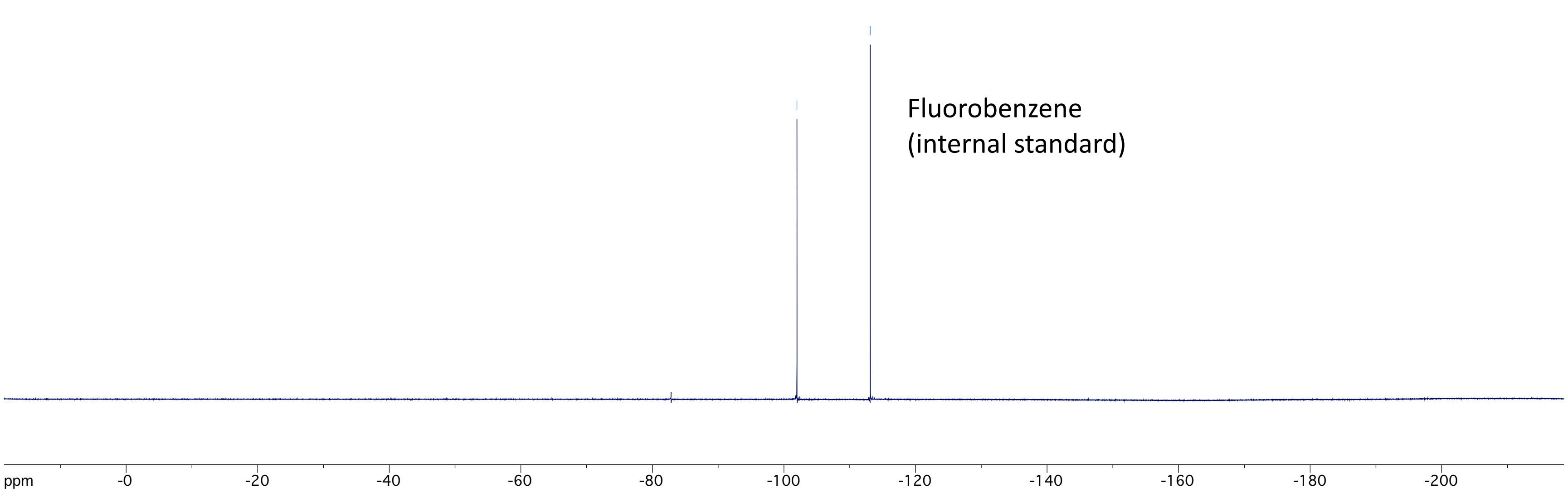

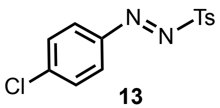

13
^{1}H NMR
400 MHz, $CDCl_3$

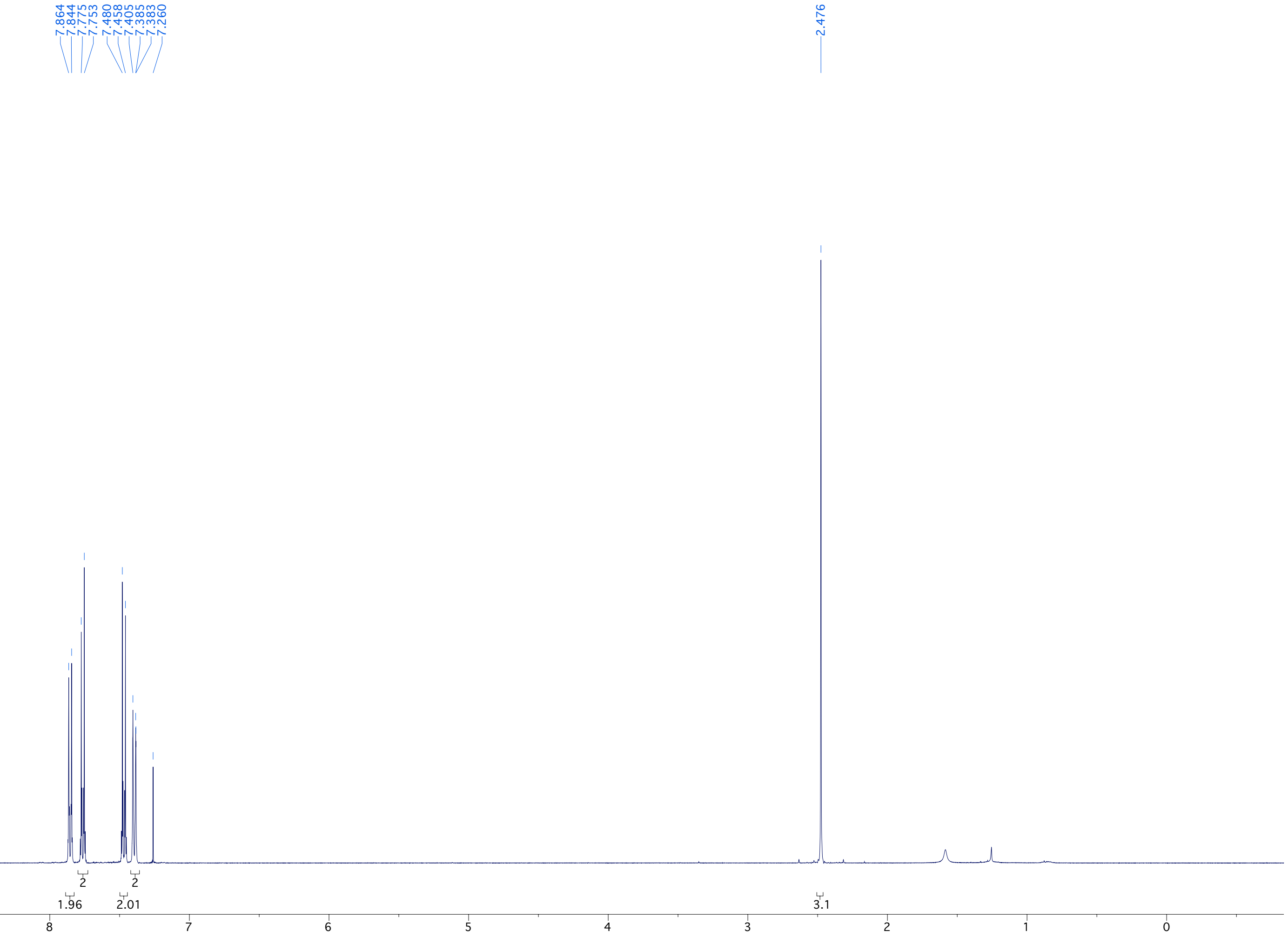

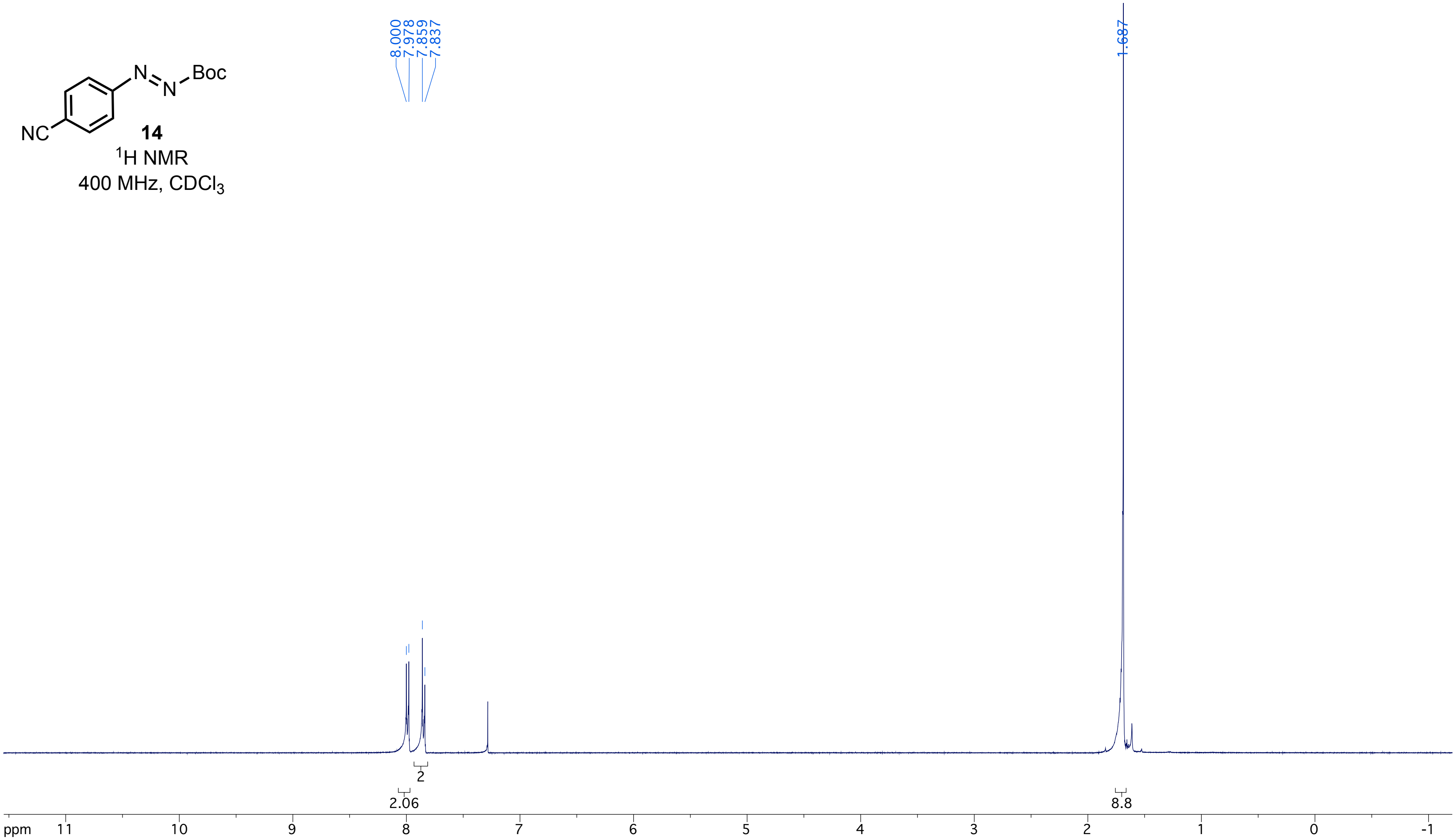
N
N
Boc
NC
14
1H NMR
400 MHz, CDCl3
8.000
7.978
7.859
7.837
1.687
2
2.06
8.8
ppm
11
10
9
8
7
6
5
4
3
2
1
0
-1

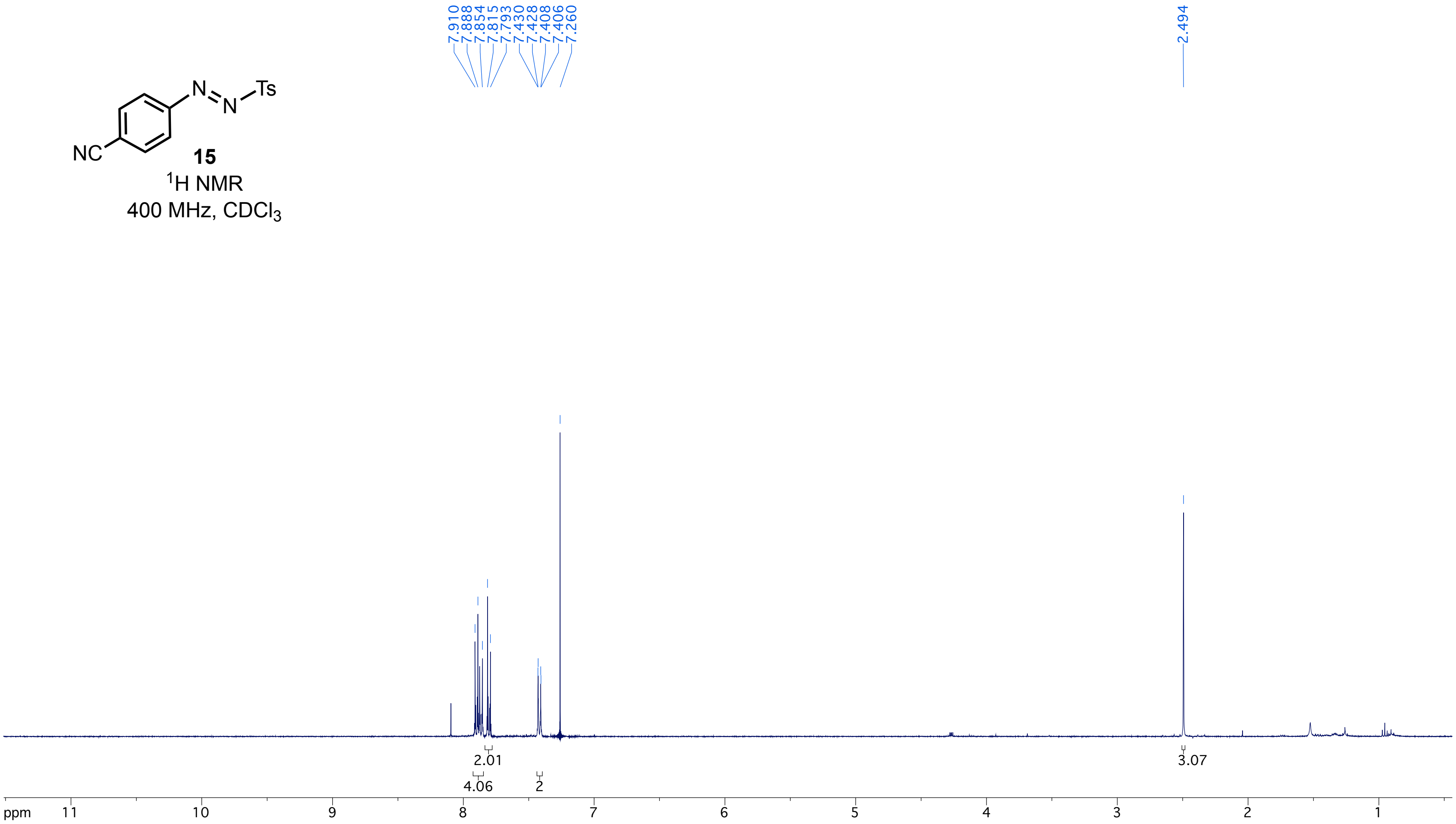
N
N
Ts
NC
15
^{1}H NMR
400 MHz, $CDCl_3$
7.910
7.888
7.854
7.815
7.793
7.430
7.428
7.408
7.406
7.260
2.494
2.01
4.06
2
3.07
ppm
11
10
9
8
7
6
5
4
3
2
1

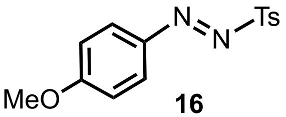

16

^{1}H NMR

400 MHz, $CDCl_3$

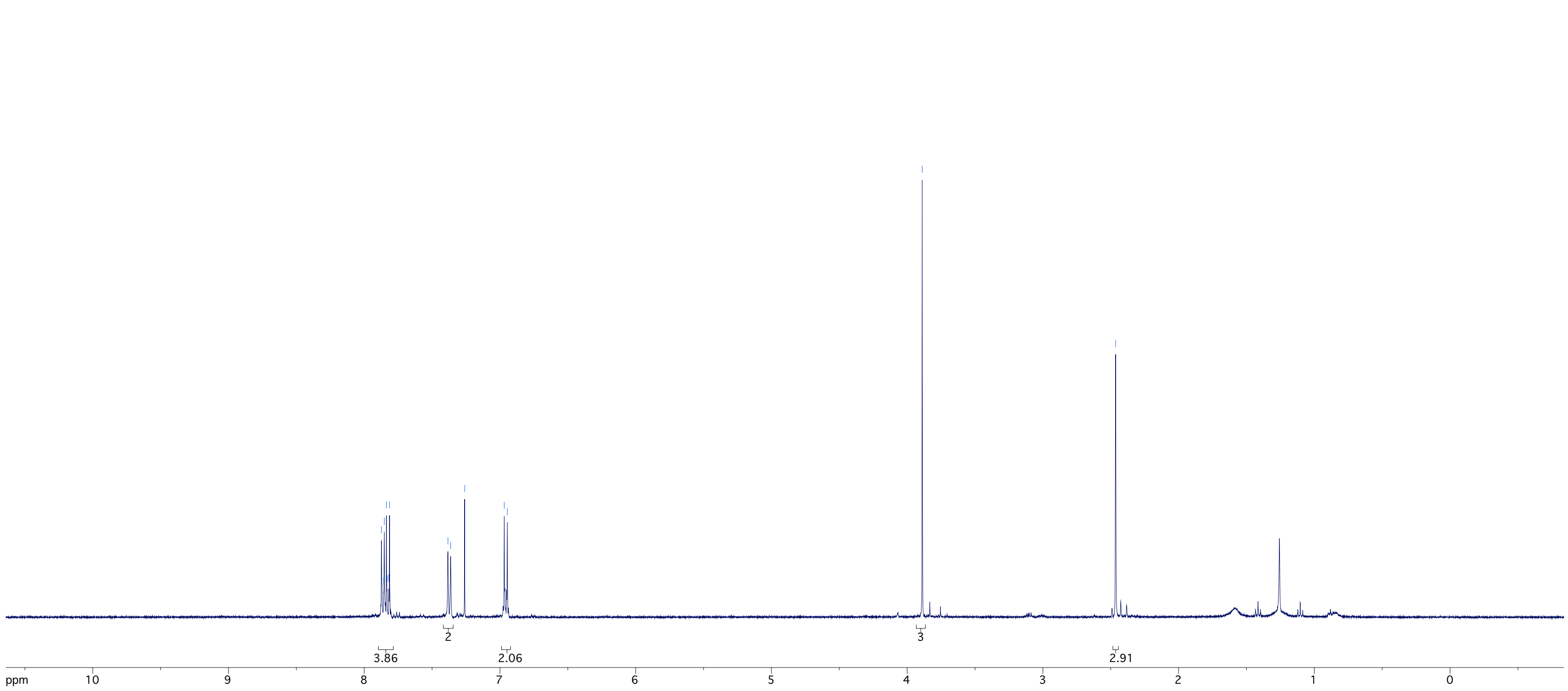

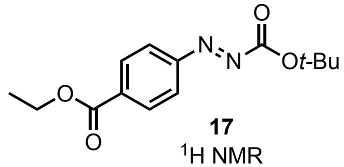

17
^{1}H NMR
400 MHz, $CDCl_3$

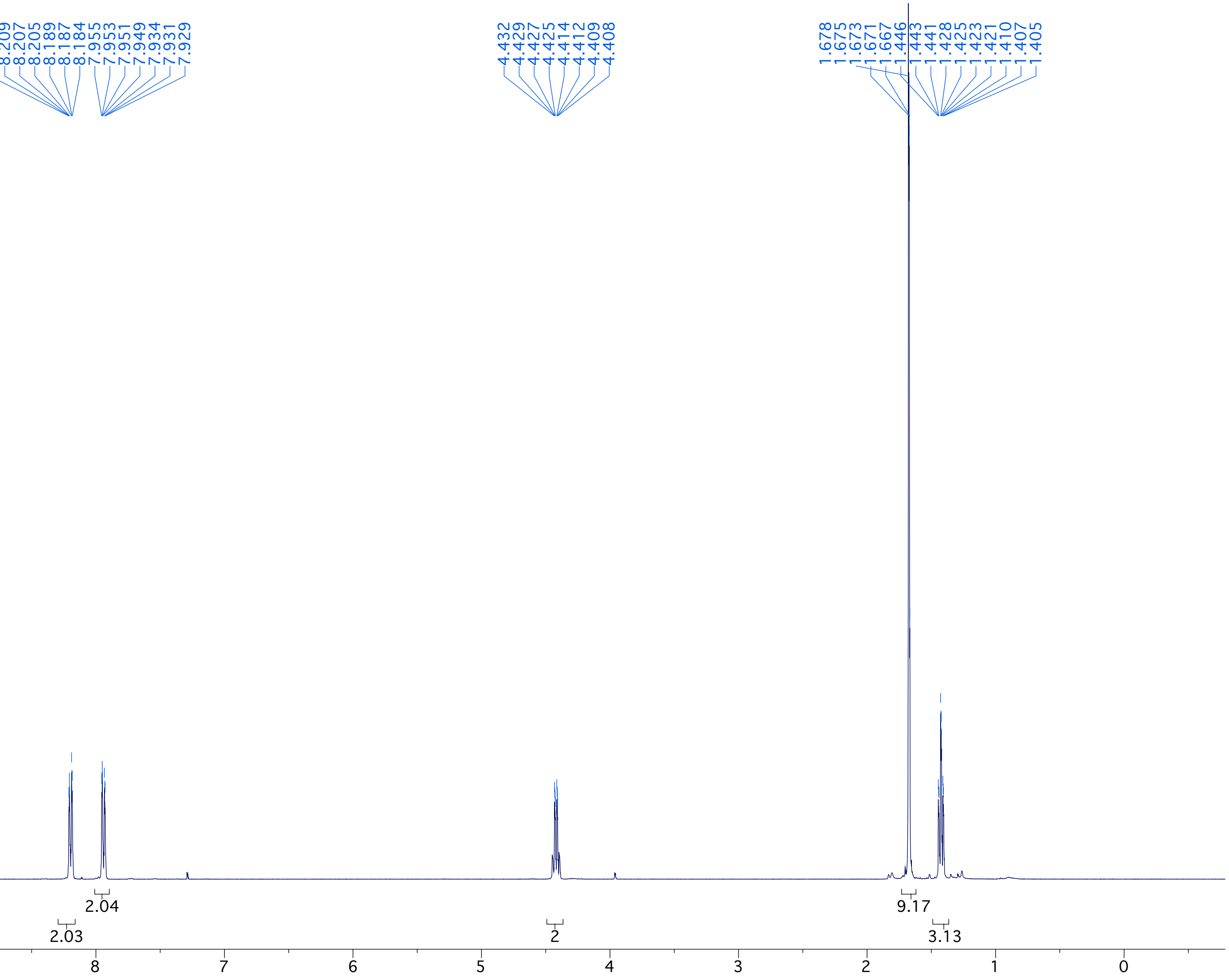

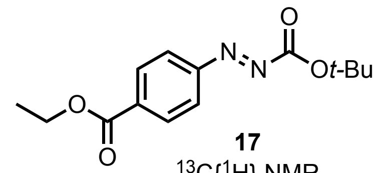

17

$^{13}C\{^1H\}$ NMR

100 MHz, $CDCl_3$

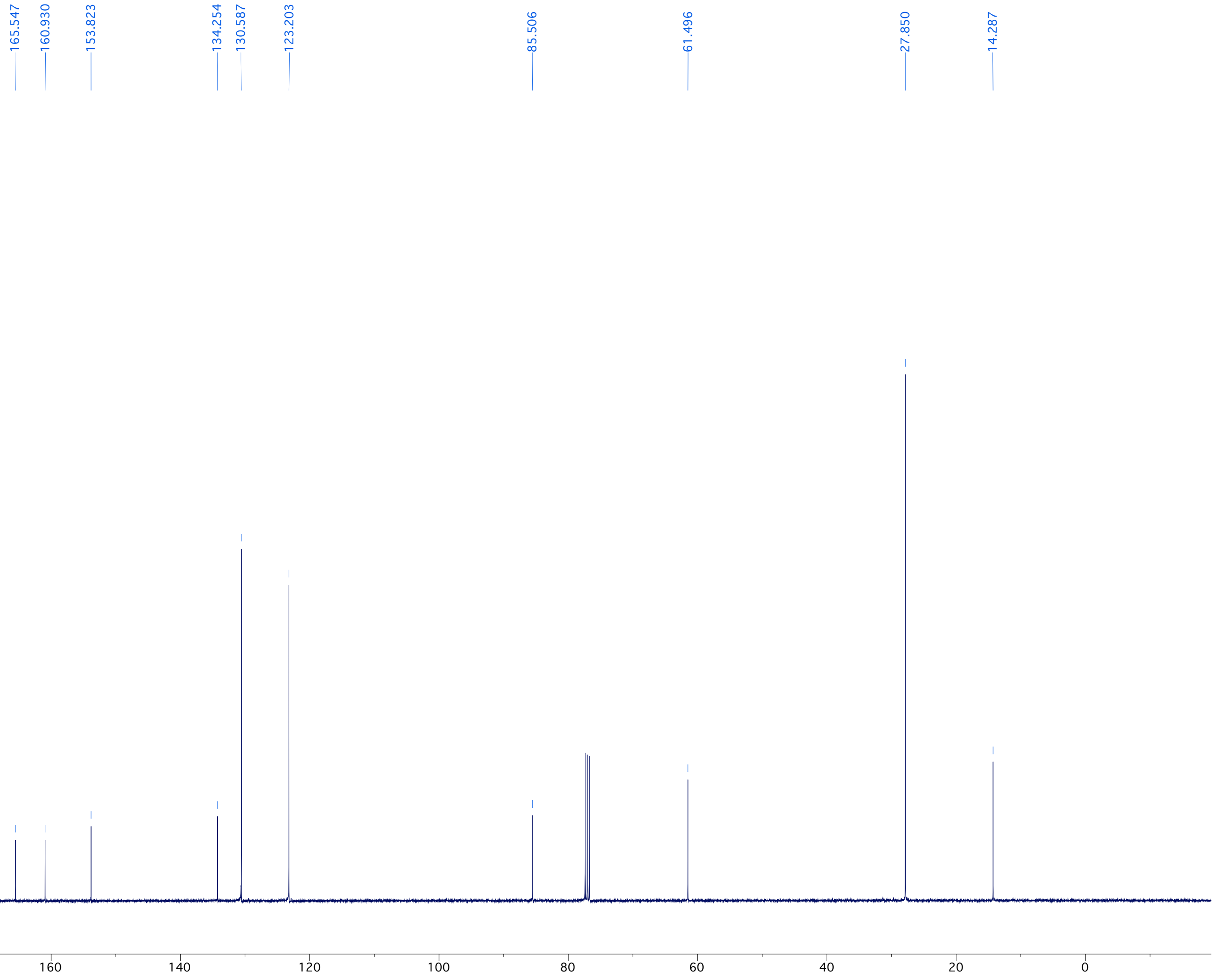

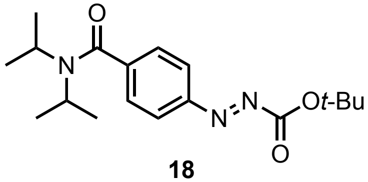

18

^1H NMR

400 MHz, $CDCl_3$

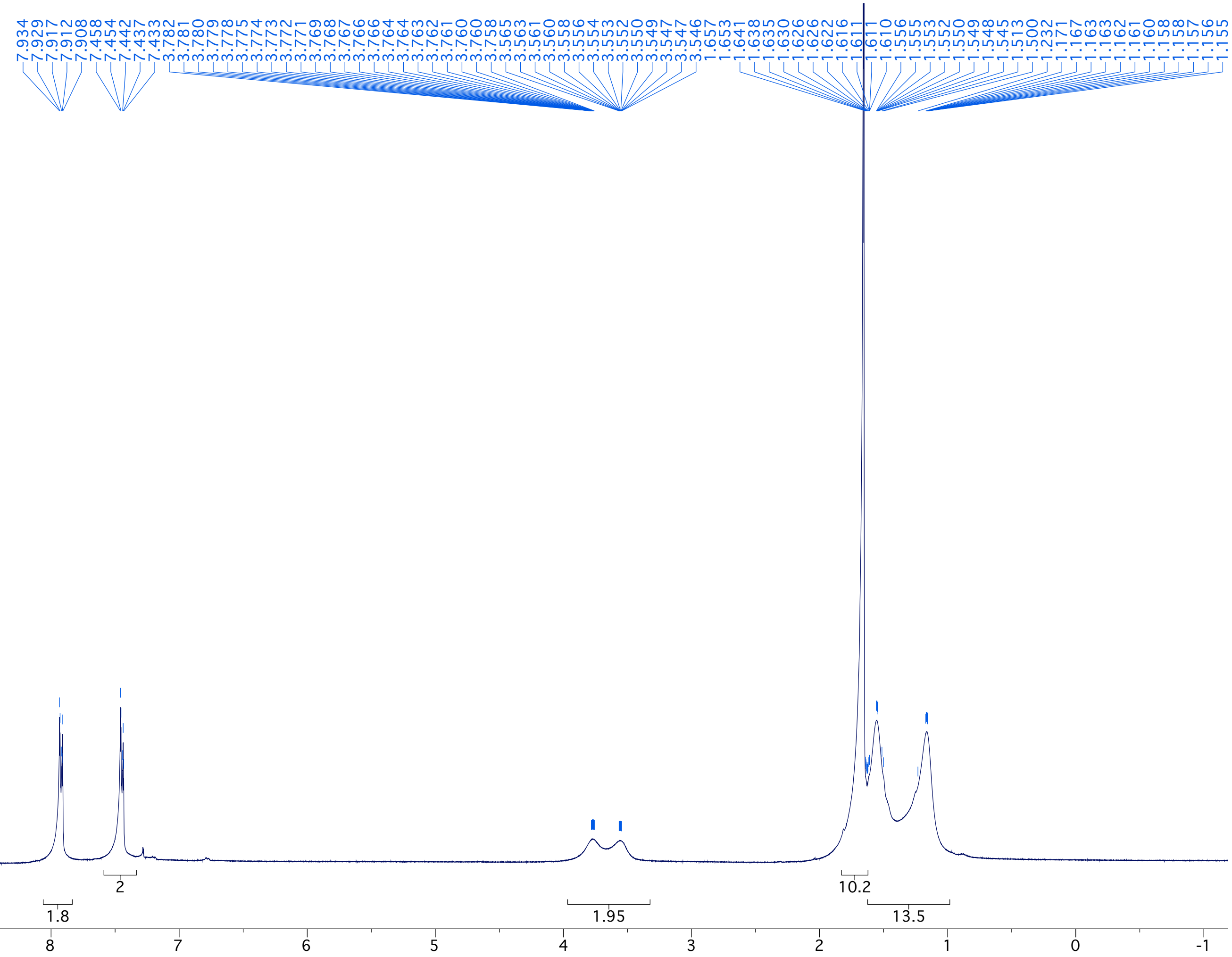

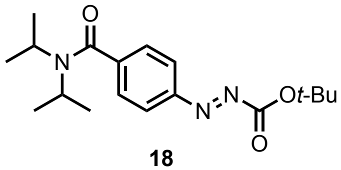

18

$^{13}C\{^1H\}$ NMR

100 MHz, $CDCl_3$

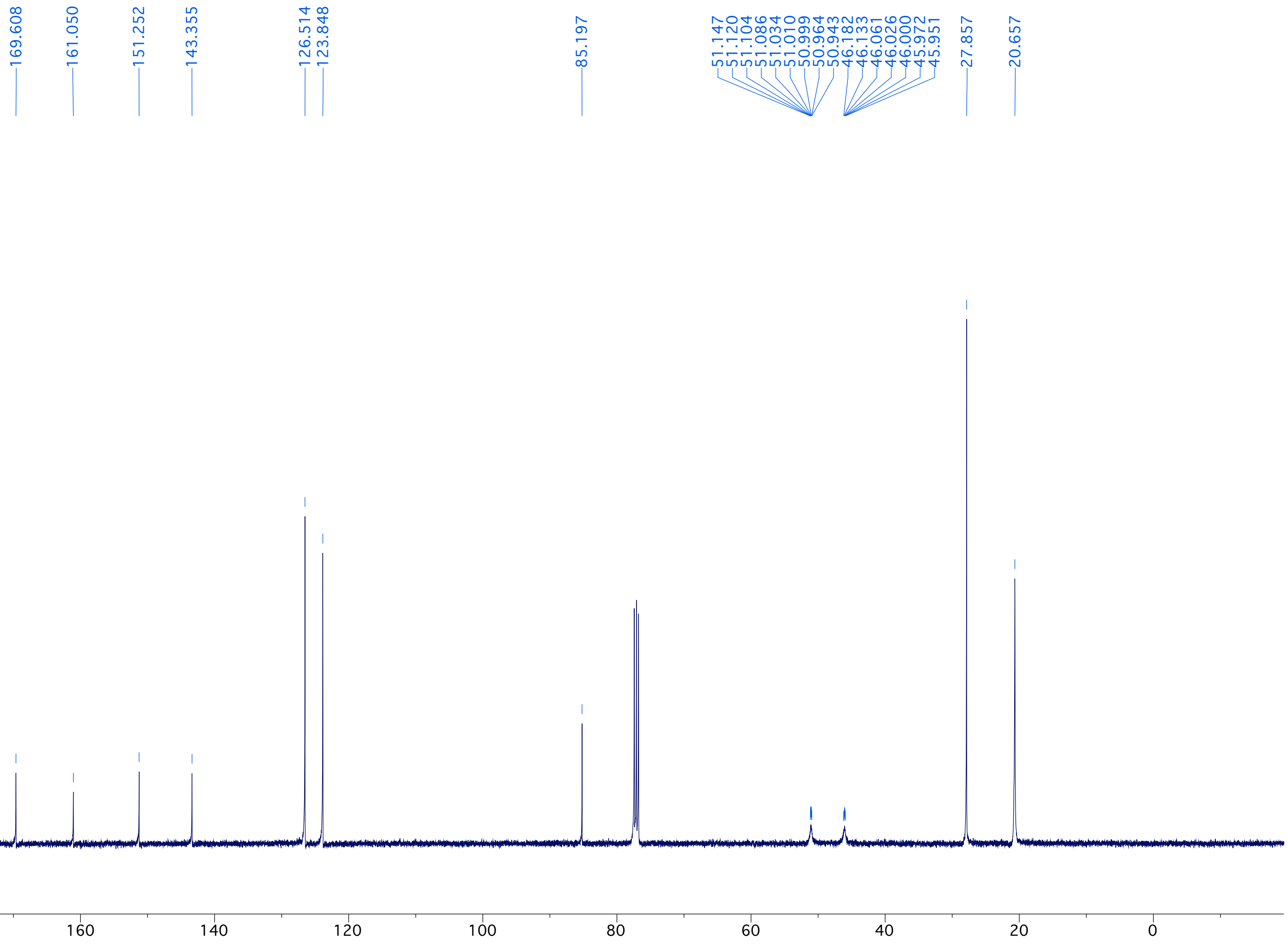

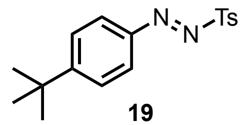

19

^{1}H NMR

400 MHz, $CDCl_3$

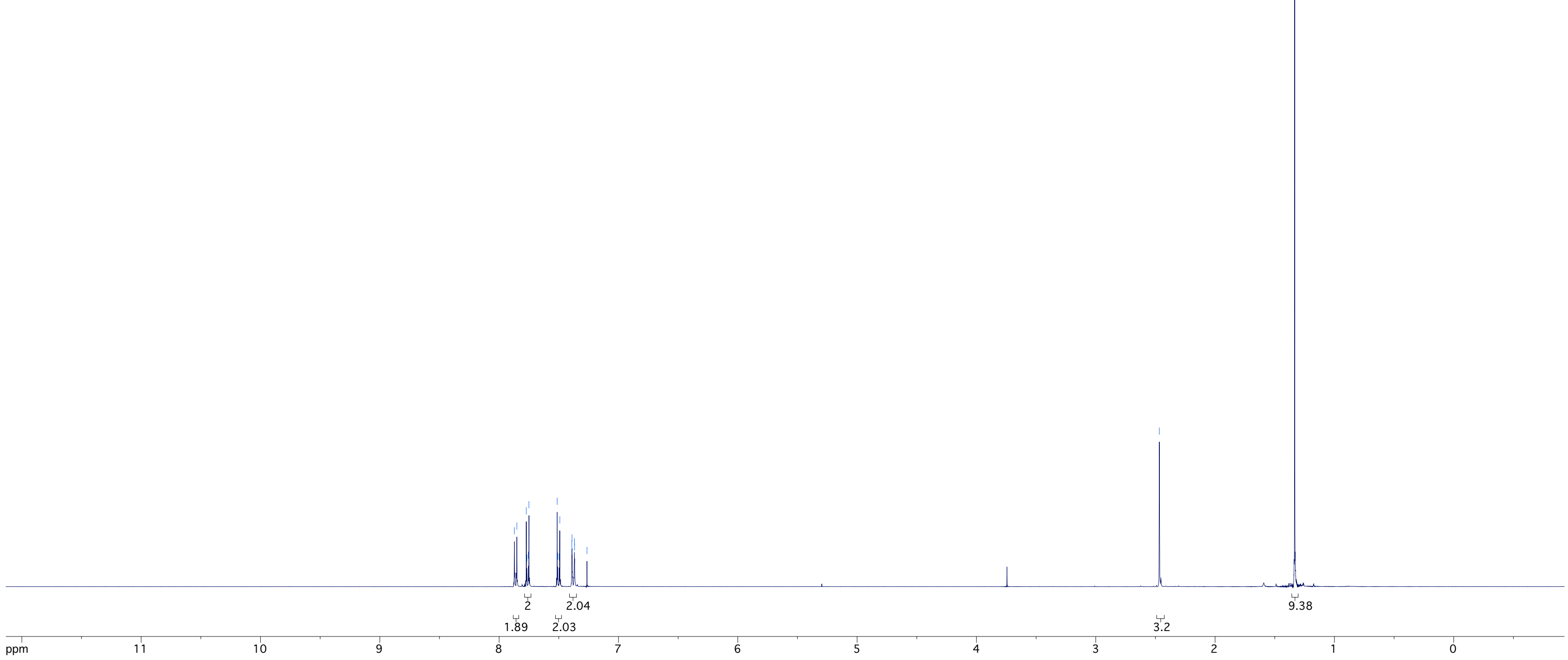

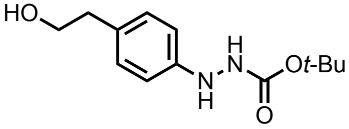

26
^{1}H NMR
400 MHz, $CDCl_3$

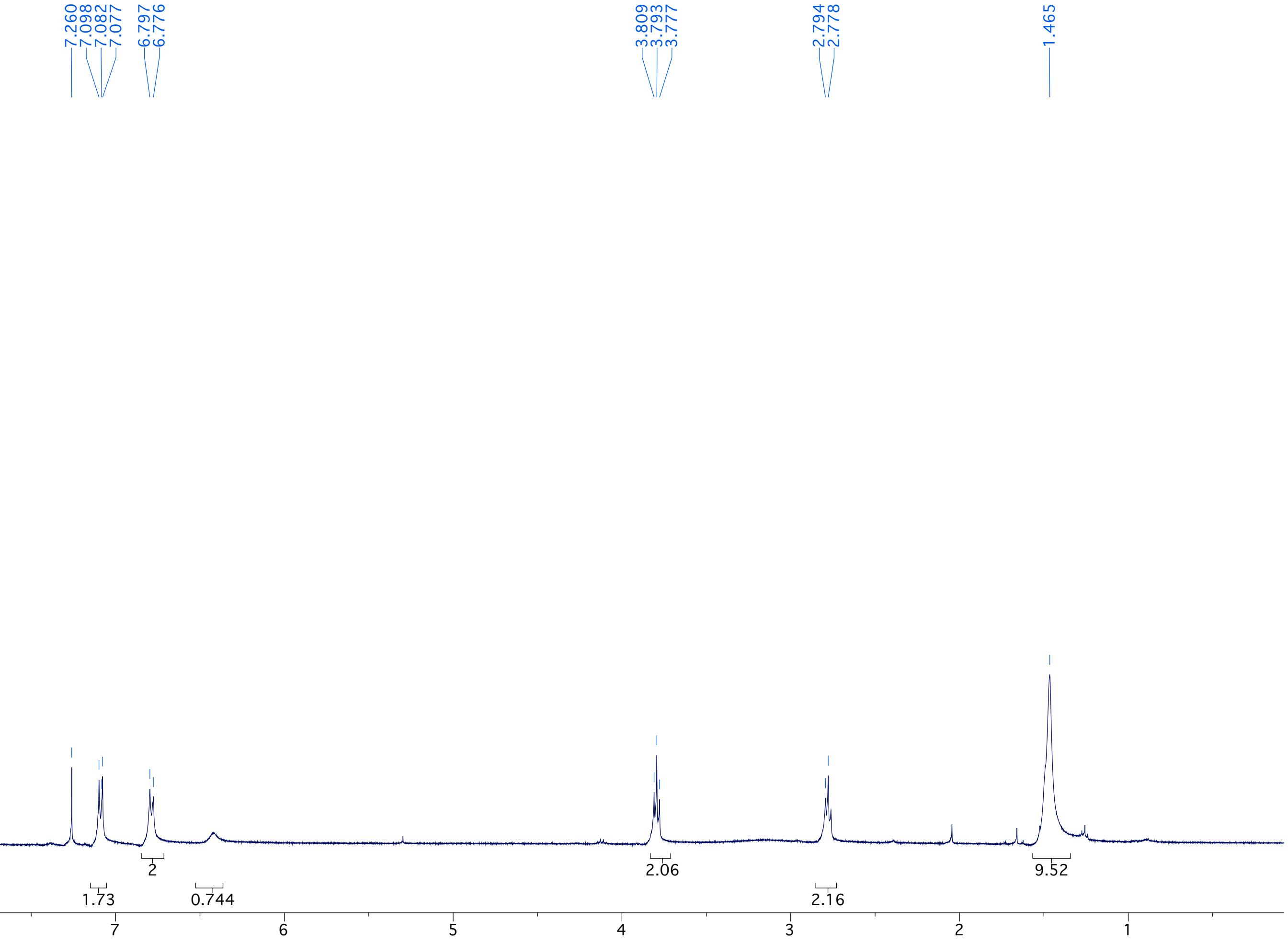

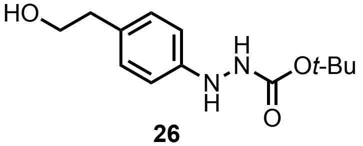

26

$^{13}C\{^1H\}$ NMR

100 MHz, $CDCl_3$

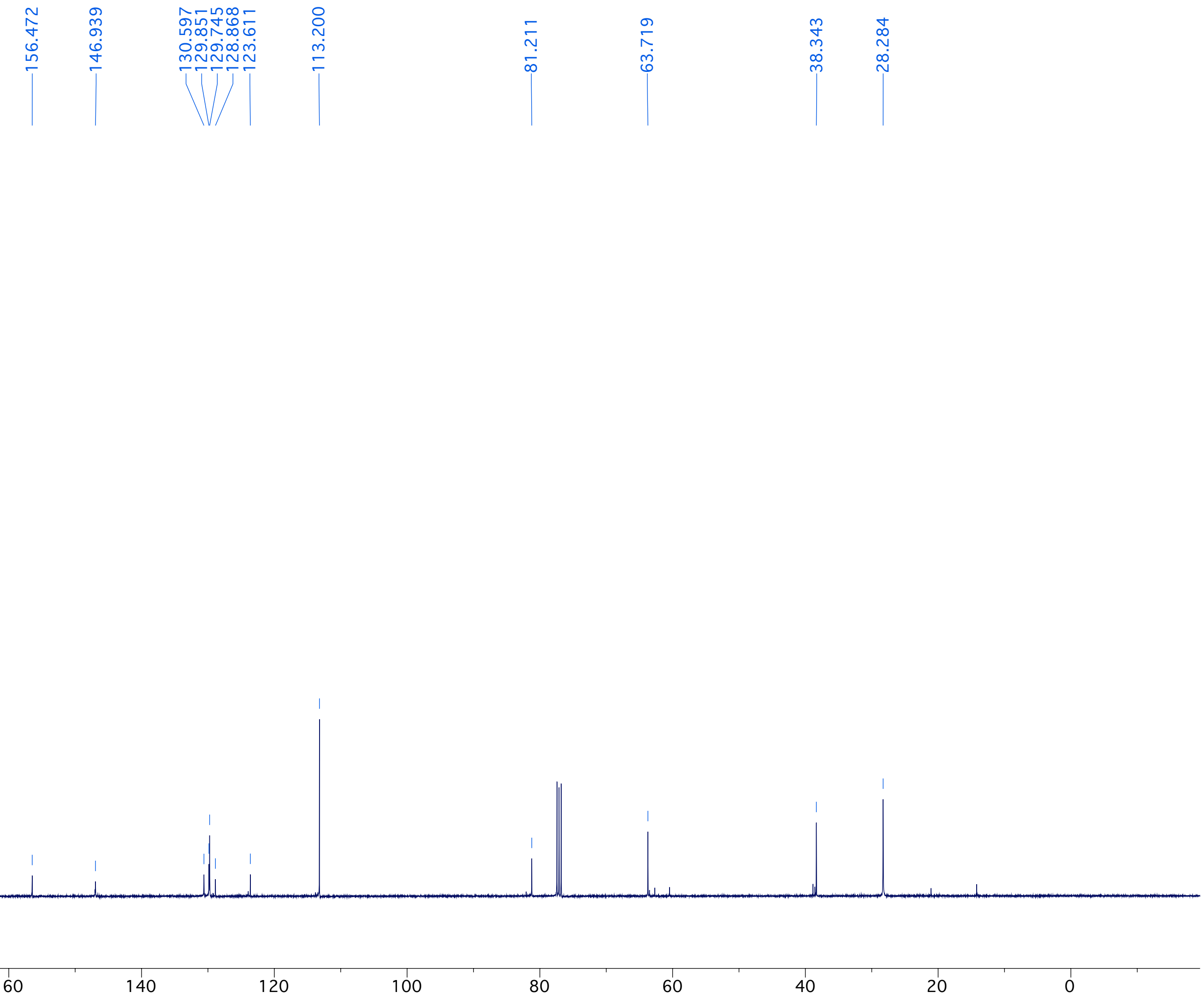

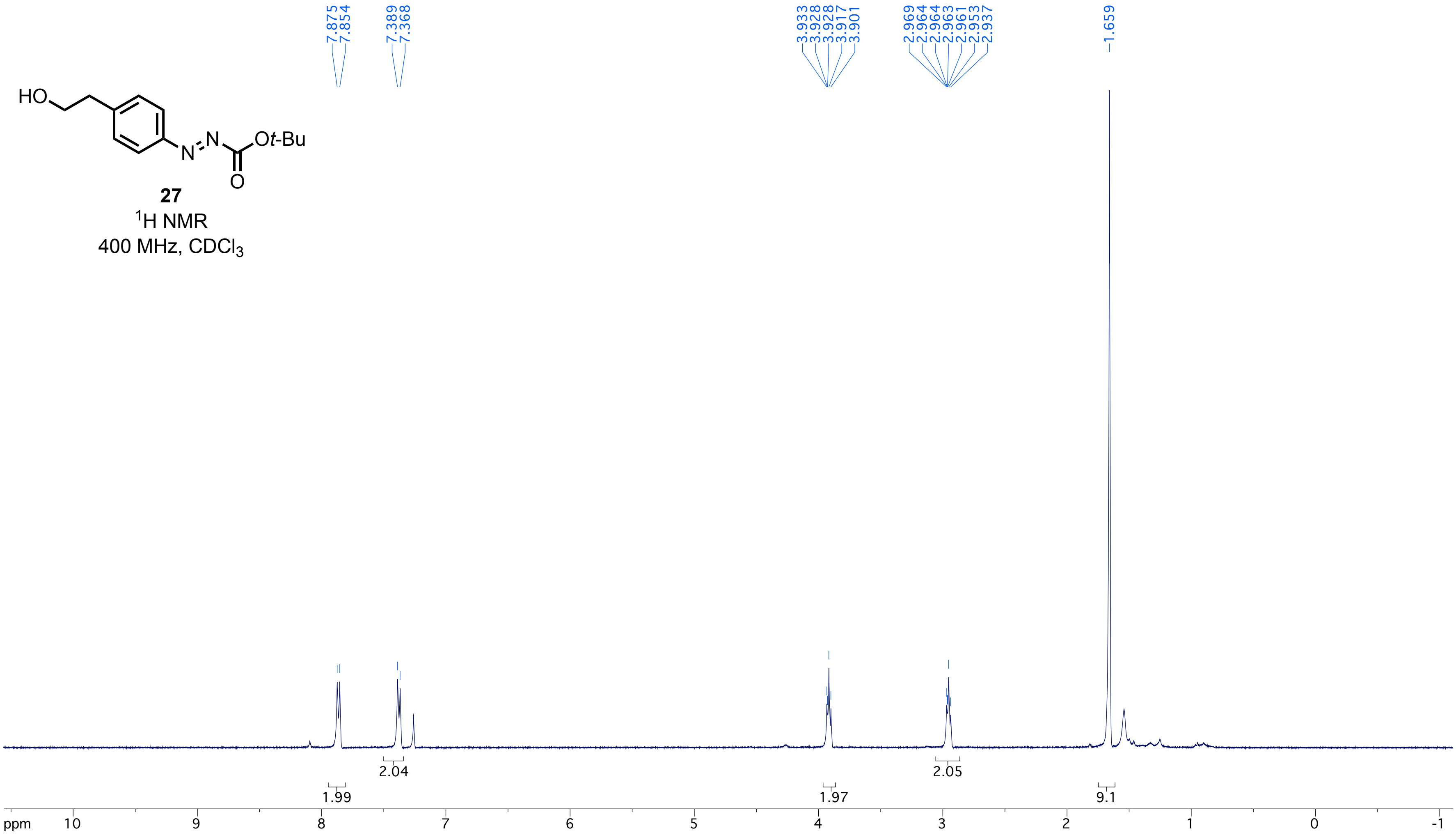

HO
N
N
O*t*-Bu
O
27
¹H NMR
400 MHz, CDCl3
7.875
7.854
7.389
7.368
3.933
3.928
3.928
3.917
3.901
2.969
2.964
2.964
2.963
2.961
2.953
2.937
1.659
2.04
1.99
1.97
2.05
9.1
ppm
10
9
8
7
6
5
4
3
2
1
0
-1

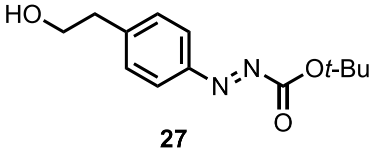

27

$^{13}C\{^{1}H\}$ NMR

100 MHz, $CDCl_3$

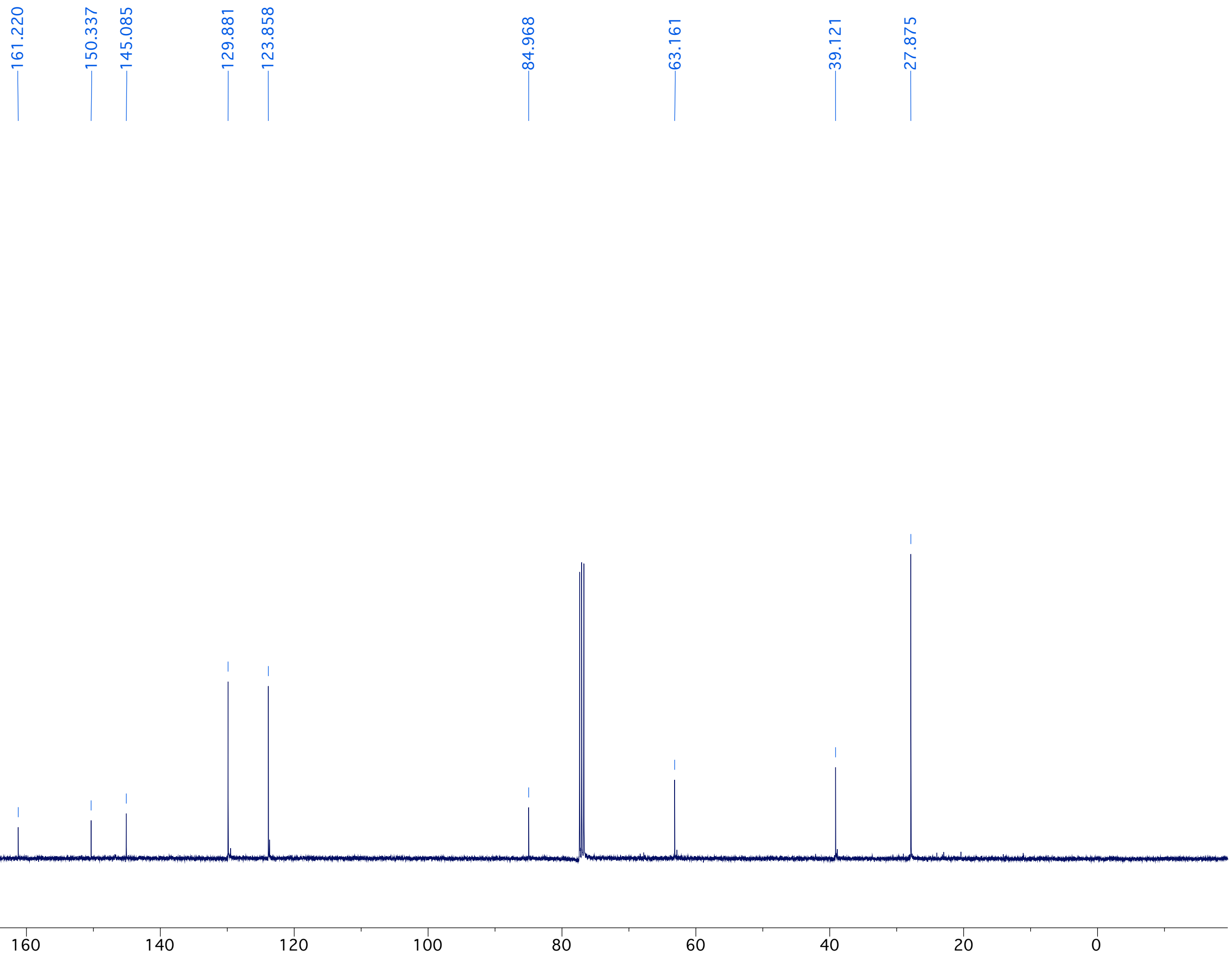

Supplement: Supplementary file 1 — jo3c02752_si_001.pdf [file jo3c02752_si_001.pdf]
